# Multi-resonant thermally activated delayed fluorescence emitters based on tetracoordinate boron-containing PAHs: colour tuning based on the nature of chelates†‡

**DOI:** 10.1039/d1sc05692a

**Published:** 2022-01-04

**Authors:** Guoyun Meng, Lijie Liu, Zhechang He, David Hall, Xiang Wang, Tai Peng, Xiaodong Yin, Pangkuan Chen, David Beljonne, Yoann Olivier, Eli Zysman-Colman, Nan Wang, Suning Wang

**Affiliations:** Key Laboratory of Cluster Science, Ministry of Education of China, Beijing Key Laboratory of Photoelectronic/Electrophotonic Conversion Materials, School of Chemistry and Chemical Engineering, Beijing Institute of Technology Beijing P. R. China nanwang@bit.edu.cn; Intelligent Organic Luminescent Materials Research Center, School of Science, Henan Agricultural University Zhengzhou Henan P. R. China; Department of Chemistry, Queen's University Kingston Ontario K7L 3N6 Canada; Organic Semiconductor Centre, EaStCHEM School of Chemistry, University of St Andrews St Andrews Fife KY16 9ST UK eli.zysman-colman@st-andrews.ac.uk; Laboratory for Chemistry of Novel Materials, University of Mons 7000 Mons Belgium; School of Materials Science & Engineering, Jiamusi University Jiamusi Heilongjiang 154007 P. R. China pt@jmsu.edu.cn; Unité de Chimie Physique Théorique et Structurale, Laboratoire de Physique du Solide, Namur Institute of Structured Matter, Université de Namur Rue de Bruxelles, 61 5000 Namur Belgium

## Abstract

Multi-resonant thermally activated delayed fluorescence (MR-TADF) materials have attracted considerable attention recently. The molecular design frequently incorporates cycloboration. However, to the best of our knowledge MR-TADF compounds containing nitrogen chelated to boron are still unknown. Reported herein is a new class of tetracoordinate boron-containing MR-TADF emitters bearing C^N^C- and N^N^N-chelating ligands. We demonstrate that the replacement of the B–C covalent bond in the C^N^C-chelating ligand by the B–N covalent bond affords an isomer, which dramatically influences the optoelectronic properties of the molecule. The resulting N^N^N-chelating compounds show bathochromically shifted absorption and emission spectra relative to C^N^C-chelating compounds. The incorporation of a *tert*-butylcarbazole group at the 4-position of the pyridine significantly enhances both the thermal stability and the reverse intersystem crossing rate, yet has a negligible effect on emission properties. Consequently, high-performance hyperfluorescent organic light-emitting diodes (HF-OLEDs) that utilize these molecules as green and yellow-green emitters show a maximum external quantum efficiency (*η*_ext_) of 11.5% and 25.1%, and a suppressed efficiency roll-off with an *η*_ext_ of 10.2% and 18.7% at a luminance of 1000 cd m^−2^, respectively.

## Abstract 2

A new class of tetra-coordinate boron-containing MR-TADF emitters and their corresponding high-performance hyperfluorescent organic light-emitting diodes have been successfully achieved.

## Graphical abstract

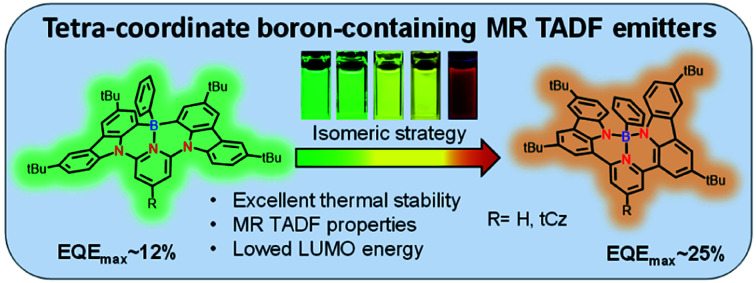

## Introduction

**Chart 1:**
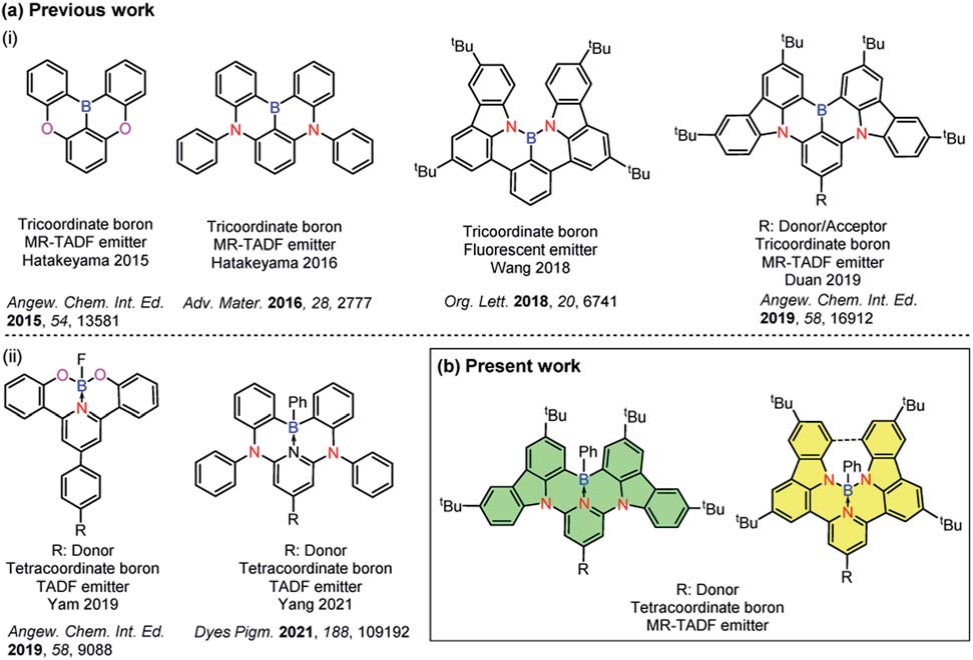
(a) Chemical structures of previously reported (i) tri- and (ii) tetra-coordinate boron compounds and (b) present work.

Incorporation of heteroatoms into π-conjugated systems has been shown to be a powerful method to alter the physical, chemical and optoelectronic properties of polycyclic aromatic hydrocarbons (PAHs) while preserving the same conjugated skeleton.^1^ Owing to the unique electron-deficient character and Lewis acidity of boron atoms,^2–4^ tricoordinate boron containing PAHs have emerged as a promising class of materials for optoelectronic applications.^5–7^ Several examples of photoactive X–B–X (X = O, S, N)-embedded aromatic molecules have been reported^8–14^ [Chart 1a(i)]. In 2011, Adachi and co-workers reported the first purely organic thermally activated delayed fluorescence (TADF) molecule, which opened an avenue for the development of a third generation of highly efficient emitters for organic light-emitting diodes (OLEDs) that offer promise to supplant the state-of-the-art transition metal based phosphorescent emitters.^15^ Recently considerable attention has been devoted to investigate B-doped PAHs that can be used as TADF-OLEDs^14,16,17^ and multi-resonance TADF (MR-TADF) OLEDs,^3,18–22,33^ the latter being a subclass of TADF emitters showing narrowband emission.

Compared with tricoordinate boron containing PAHs, the majority of existing tetracoordinate boron-conjugated systems commonly include the intramolecular B ← N coordination.^23–25^ The incorporation of a B ← N bond in such systems not only provides structural rigidity and π-electron delocalization, but also increases the electron affinity of conjugated systems. However, examples of TADF-OLEDs based on these tetracoordinate boron compounds are still rare,^26–30^ which may be attributed in part to the ligand cleavage that results in the formation of facial isomers.^31^ Utilization of a pincer ligand to construct a tridentate chelate framework can be a practical strategy to improve the stability of these boron-containing materials [Chart 1a(ii)]. In 2019, Yam and co-workers reported the first stable and efficient O^N^O-chelating tetracoordinate boron emitters.^27^ High-performance green-emitting vacuum-deposited OLEDs using these materials showed up to 18.0% maximum external quantum efficiency (EQE_max_). In 2021, Yang and co-workers also reported two distinct white and green emitting TADF-OLEDs that showed over 10% EQE_max_. In that work, C^N^C-chelated tetracoordinate boron compounds were used as emitters.^26^ Despite these examples, tetracoordinated boron-based TADF examples are rare, with no example of this type of emitter undergoing MR-TADF.

Herein, we report a structurally distinct new class of MR-TADF compounds containing a tetracoordinate boron, demonstrating several isomers. They contain either intramolecular C^N^C-chelates or N^N^N-chelates to boron, and were formed as a result of modification of the connecting atoms between the 3,6-di-*tert*-butylcarbazole (TCz) and the pyridine ring in the backbone. Upon changing two of the bonding atoms to the boron centre from carbon to nitrogen, the emission colour was effectively tuned from green to deep red. The introduction of a pendant TCz unit at the 4-position of the central pyridine ring [TCz(4)] significantly enhanced the reverse intersystem crossing (RISC) rate, which is manifested in the shortened delayed fluorescence lifetime, without adversely affecting the photoluminescence quantum yield. Using these excellent MR-TADF molecules, a series of green and yellow-green OLEDs were successfully fabricated. This work demonstrates a novel strategy for constructing MR-TADF emitters using rigid and planar structures whose properties can be modified through isomeric molecular engineering.

## Results and discussion

### Synthesis and characterization

**Scheme 1:**
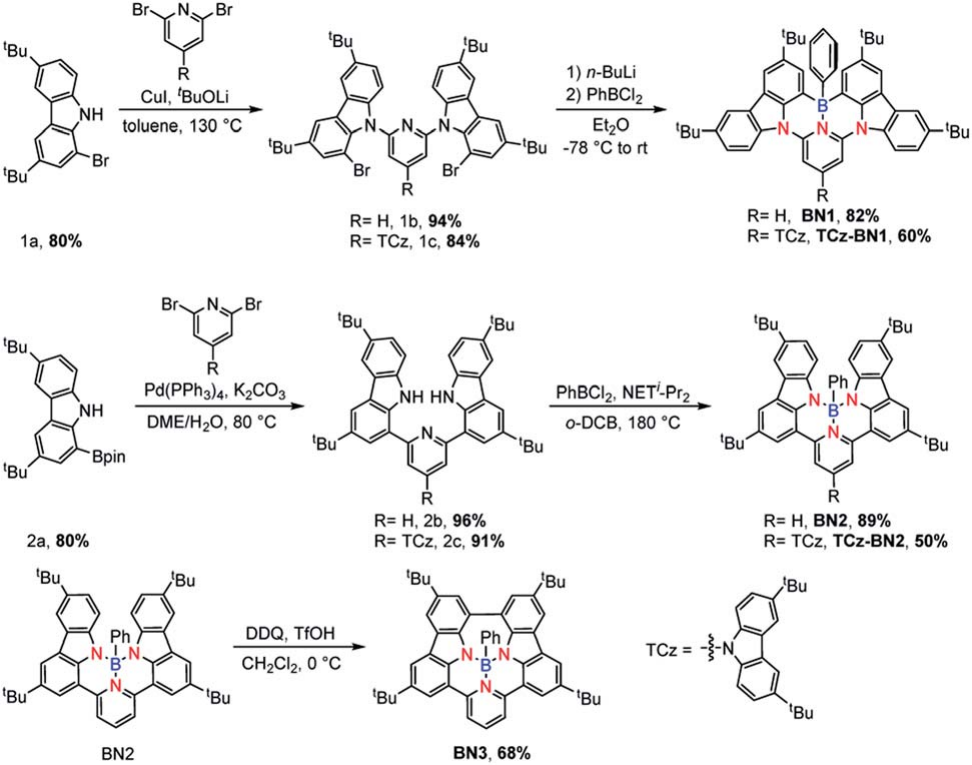
Synthetic route of BN1, TCz-BN1, BN2, TCz-BN2 and BN3.

The isomeric families of compounds were synthesized based on the location where the TCz and pyridine units are attached: C^N^C (1b/1c) and N^N^N (2b/2c). As shown in Scheme 1, the ligands were obtained in two or three steps from commercially available starting materials in good yields (50–89%), involving an Ullmann condensation (1b/1c) or a palladium-catalyzed double Suzuki–Miyaura coupling reaction (2b/2c). The boron centre was then introduced under electrophilic borylation reaction conditions. The fully conjugated molecule BN3 was obtained through oxidative intramolecular C–C coupling of compound BN2 in a yield of 68%. All compounds were fully characterized by NMR spectroscopy and high-resolution mass spectrometry (HRMS). The characterization details are provided in the ESI.‡

**Fig. 1:**
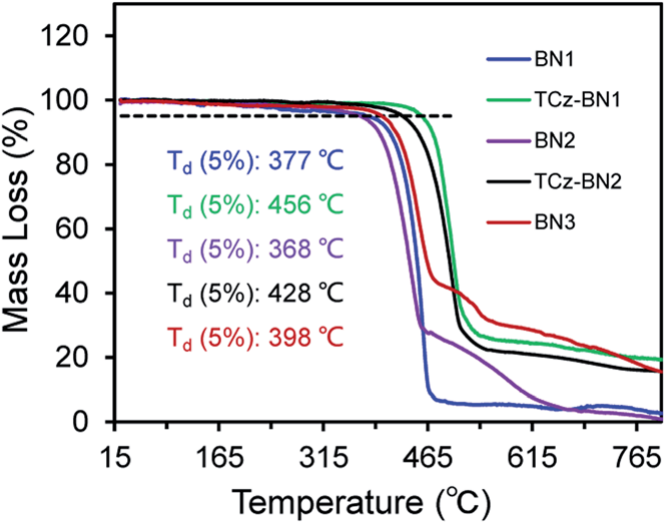
Thermogravimetric curves of all the compounds.

At room temperature, the ^11^B NMR resonances of these compounds in CD_2_Cl_2_ display sharp peaks between 4.10 and 4.96 ppm, indicating the tetracoordinate geometry of the boron centre. Considering that the thermal stability of emitters is a pivotal parameter for OLED device fabrication, thermogravimetric analysis (TGA) was performed for vacuum-sublimed samples. As shown in Fig. 1, all compounds display excellent thermal stability with decomposition temperatures (*T*_d_ defined as the temperature where 5% loss of initial weight is reached) in the range of 377–456 °C, which are much higher than those of other N,C-^34^ and N,N-chelate^30,35^ organoboron compounds and comparable to those of O^N^O tridentate organoboron emitters.^27^ Notably, incorporation of an extra TCz unit at the 4-position of the pyridine moiety results in higher decomposition temperatures (*T*_d_ = 456 °C and 428 °C for TCz-BN1 and TCz-BN2, respectively) than those of the parent compounds (*T*_d_ = 377 °C and 368 °C for BN1 and BN2, respectively). The glass transition temperatures (*T*_g_) of TCz-BN1 and BN2 are 256 °C and 248 °C, respectively. Unfortunately, the *T*_g_ values were undetectable for BN1, TCz-BN2 and BN3 (Fig. S1.1‡). Both the TGA and *T*_g_ data indicate that all these compounds are suitably thermally stable to be considered as candidates for OLED fabrication.

### Crystal structures

**Fig. 2:**
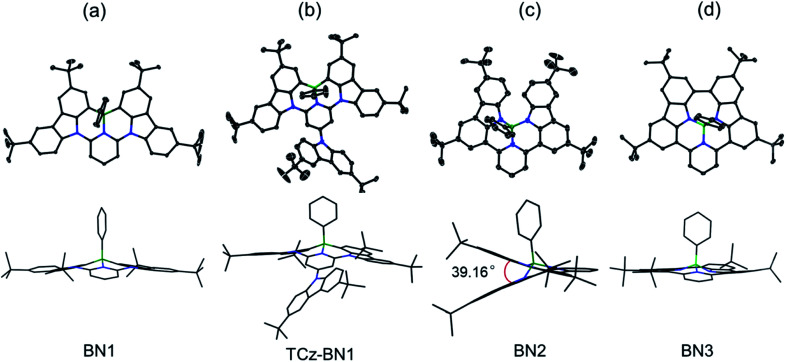
X-ray crystal structures of (a) BN1, (b) TCz-BN1, (c) BN2, and (d) BN3, and the side views (bottom) of the structures in stick style. Hydrogen atoms and solvent molecules are omitted for clarity. The thermal ellipsoids are drawn at the 50% probability level.

The crystal structures of BN1, TCz-BN1, BN2 and BN3 are shown in Fig. 2, which confirm the existence of the intramolecular B ← N interactions (Fig. 2). BN1 and TCz-BN1 crystallize in the monoclinic *P*2_1_/*n* space group with four molecules in a primitive unit cell. This structural feature effectively inhibits intermolecular π⋯π interactions, leading to an irregular packing of molecules in the crystal lattice. A BN1 molecule forms an extended 3D structure in the crystal lattice *via* four C–H⋯π interactions with distances between 2.589 and 2.715 Å (Fig. S2.2‡). Compared with BN1, besides the weak C–H⋯π interactions with distances from 2.820 to 3.022 Å between the peripheral TCz units, TCz-BN1 also displays weak intermolecular π⋯π interactions with an average distance of 3.84 Å between two adjacent molecules, which is confirmed by its crystal packing diagram (Fig. S2.4‡). Additionally, the TCz(4) unit in TCz-BN1 demonstrates a twisted arrangement with respect to the central backbone, as evidenced by the dihedral angle of 43.5° (Fig. S2.5‡). Furthermore, the B ← N bond lengths in BN1 and TCz-BN1 (1.679 Å and 1.698 Å, respectively) are much longer than those of other reported C^N^C (1.572 to 1.617 Å)^26,36^ and O^N^O (1.579 Å)^27^ tridentate boron compounds due to the significant steric congestion introduced by the two TCz units.

As a result of the tetrahedral geometry of the boron centre in both BN2 and BN3, the B–N_TCz_ bond lengths (1.50–1.54 Å) are within the typical range of B–N single bonds, and longer than the analogous bonds in N^B^N-doped PAHs (1.40–1.41 Å).^12^ Due to the great steric repulsion between the two bulky TCz units, BN2 adopts a twisted conformation with a dihedral angle of 39.16° between the TCz units (Fig. 2). A dimeric structure is also observed in the BN2 unit cell, which is formed through the moderate-to-weak intermolecular π⋯π interactions evidenced by short contact distances ranging from 3.147 to 4.383 Å. There is an extended 1D structure in the crystal lattice maintained *via* four CH⋯π interactions between the H on the pyridine and phenyl group, with an average distance of 2.82 Å (Fig. S2.7‡). In contrast, the C–C coupled product BN3 is nearly planar, with a very small dihedral angle of 4.79° between the two TCz units. The separation distances between the conjugated backbone planes are between 3.202 Å and 3.667 Å. Therefore, compared with BN2, stronger π⋯π intermolecular interactions are expected for BN3 molecules in the crystal lattice. There are also CH⋯π interactions between H of the isolated phenyl group and the fused benzene rings of the TCz units with a distance of 2.677 Å.

### Theoretical modelling

Previous investigations by some of us revealed the requirement for employing high level quantum chemical methods such as the Spin-Component Scaling second-order approximate Coupled-Cluster (SCS-CC2) including (partially) double excitations in order to accurately compute the excited states of doped polyaromatic hydrocarbons.^37^ The materials presented here are reminiscent of previously reported MR-TADF emitters,^18^ with a pattern for the difference density between the first singlet (S_1_) or triplet (T_1_) excited states and the ground state (S_0_) displaying alternating increasing and decreasing density on adjacent sites, near the pyridyl ring primarily. The short-range charge transfer (SRCT) observed between adjacent atoms ensures a suitably small Δ*E*_ST_ to enable TADF.^37^ In order to maintain a reasonable computational cost, we omitted the weakly electron-donating ^*t*^Bu groups decorating the peripheral carbazole units in TCz-BN1 and TCz-BN2 (renamed Cz-BN1 and Cz-BN2 respectively). Using SCS-CC2/cc-pVDZ, small Δ*E*_ST_ values of 0.07 eV, 0.05 eV, 0.08 eV, 0.06 eV and 0.03 eV are predicted for BN1, CzBN1, BN2, CzBN2 and BN3, respectively, which are in fairly good agreement with the experimentally determined singlet–triplet energy gaps Δ*E*_ST_ (*vide infra*). We previously identified that, unlike for conventional D–A TADF materials, time-dependent density functional theory (TD-DFT) poorly predicts the Δ*E*_ST_ of MR-TADF compounds.^37^ When we applied common DFT functionals (Tables S3.1–S3.5‡), we observed that these calculations consistently overestimated Δ*E*_ST_, in line with previous studies.^37,38^

**Fig. 3:**
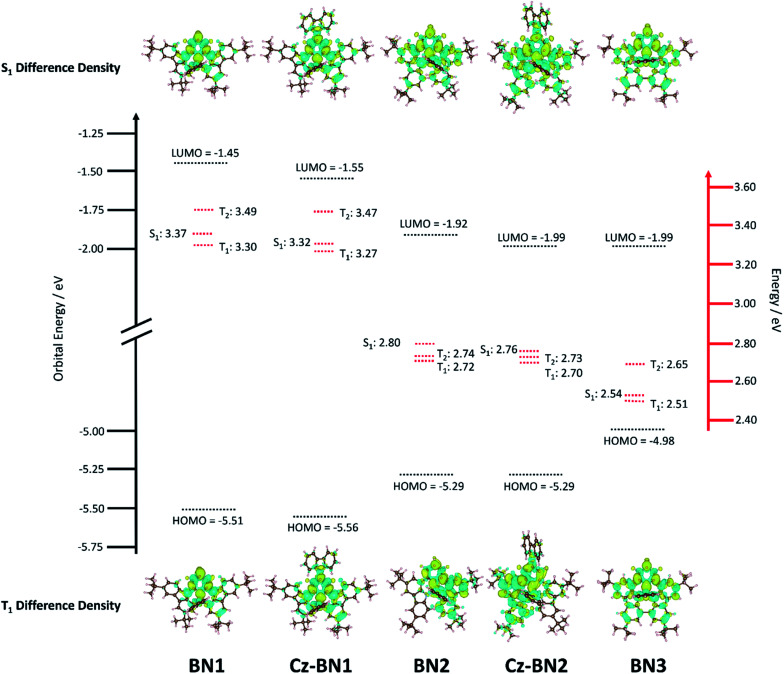
Excited state and ground state energies of the emitters where orbital energy (black) was calculated from PBE0-6-31G(d,p) and excited state energy (red) was calculated from SCS-CC2/cc-pVDZ and difference density plots of each emitter at S_1_ and T_1_.

Upon further analyzing the difference density plots, we observed a decreased density area (blue area, see Fig. 3), which goes from partially to fully delocalized on the fused carbazole units when moving from BN1, to BN2 and BN3, respectively. This corresponds to an increase in the CT distance, *D*_CT_ (Tables S3.6, S3.8 and S3.10‡), from 1.29 Å in BN1, to 2.51 Å in BN2 and 3.12 Å in BN3 that reflects an increase of the CT character of these compounds. The increased CT character of these compounds is also reflected in the stabilization of the S_1_ energies and the decrease in oscillator strength. The inclusion of the pendant Cz group has a minimal impact on the excited state properties, with only a modest predicted stabilization in S_1_, decreasing by 0.05 eV and 0.04 eV for CzBN1 and CzBN2 from their respective parents BN1 and BN2.

### Electrochemistry

**Fig. 4:**
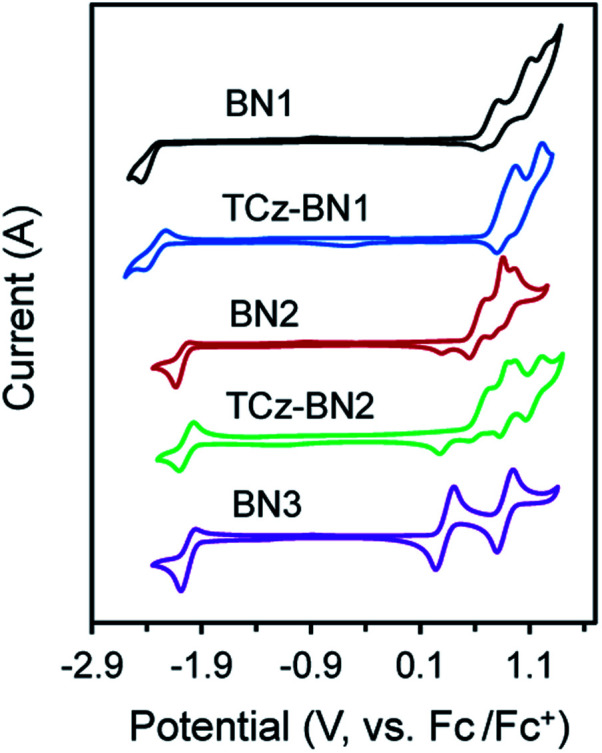
Cyclic voltammograms (CV) for all compounds. The CV curves were measured at room temperature with 0.1 M solution of [*n*-Bu_4_N]PF_6_ in dichloromethane for the oxidation and reduction scan (*vs.* Fc/Fc^+^).

The electrochemical properties of the five compounds were investigated by cyclic voltammetry (CV) and differential pulse voltammetry (DPV, Fig. S1.2‡) in dichloromethane and potentials are reported *vs.* Fc/Fc^+^. As shown in Fig. 4, BN1, TCz-BN1, BN2 and TCz-BN2 exhibit irreversible oxidation waves owing to the oxidation of the pyridine ring. The *E*_ox_ for BN1 is 0.81 V and this oxidation wave is anodically shifted by 0.07 V for TCz-BN1. When the carbazolyl units are coordinated instead to boron, there is a cathodic shift of *E*_ox_ to 0.62 V for BN2 and 0.65 V for TCz-BN2. Irreversible reduction waves were also observed for BN1 and BN2 at −2.37 V and −2.07 V, respectively. Interestingly, with the incorporation of an extra TCz unit on the pyridine ring, the reduction wave becomes quasi-reversible with an *E*_red_ of −2.33 V and −2.04 V for TCz-BN1 and TCz-BN2. These reduction waves are modestly anodically shifted compared to those in the parent compounds. For the boron-fused polycyclic aromatic compound BN3, the electrochemical behavior is distinct, with two reversible redox couples at an *E*_ox_ of 0.33 and 0.89 V and a quasi-reversible reduction wave at −2.02 V.

Based on the electrochemical data, the HOMO and LUMO energies of these compounds were estimated and are listed in Table 1. Notably, introducing an extra TCz unit on the pyridine (TCz-BN1 and TCz-BN2) has a negligible influence on their corresponding HOMO and LUMO energy levels. Additionally, compounds with a N^N^N chelating backbone (BN2, TCz-BN2 and BN3) display lower LUMO and higher HOMO energy levels compared with those with a C^N^C chelating backbone (BN1 and TCz-BN1), and thus exhibit smaller HOMO–LUMO energy gaps. Additionally, due to the enhanced conjugation of the backbone, the HOMO level of BN3 is further destabilized, resulting in a smaller HOMO–LUMO gap of 2.37 eV. The changes in HOMO and LUMO energies between the materials are well captured by DFT calculations.

**Table 1:** Photophysical and electrochemical properties of all compounds

Compounds	*λ*_abs_ [nm] 298 K (*ε*, M^−1^ cm^−1^)^a^	*λ*_PL_ [nm] 298/77 K^a^	*Φ*_PL_ [%] 298 K^b^	*τ*_PF_ [ns]/*τ*_DF_ [μs]^a^	*E*_g_ c [eV]	HOMO/LUMO^d^ [eV]	Δ*E*_ST_^e^ [eV]	FWHM^f^ [nm]
BN1	406 (22 300), 336 (9400), 296 (31 600)	514/443	92	4.7/6.9	2.81	−5.60/−2.47	0.20	82
TCz-BN1	398 (16 600), 343 (22 600), 292 (36 500)	517/464	89	6.7/5.4	2.83	−5.66/−2.52	0.16	89
BN2	465 (17 000), 310 (33 200)	567/570	64	1.2/33.2	2.36	−5.41/−2.74	0.19	97
TCz-BN2	464 (13 600), 377 (13 500), 312 (24 000)	584/571	61	1.2/18.1	2.34	−5.41/−2.79	0.17	108
BN3	531 (4500), 452 (9600), 370 (19 200), 342 (29 000)	694/648	1	1.1/—	2.00	−5.13/−2.76	—	151

a In degassed THF solution (1 × 10^−5^ M).

b Absolute quantum efficiency determined using an integrating sphere.

c Estimated from absorption edges of UV-visible spectra.

d Determined from the first oxidation and reduction peaks in the DPV recorded in THF solutions.

e Singlet (*E*_S_) and triplet (*E*_T_) energies estimated from onsets of the fluorescence and phosphorescence spectra at 77 K in 5 wt% doped PMMA films, respectively; Δ*E*_ST_ = *E*_S_ − *E*_T_.

f Full-width at half-maximum.

### Photophysical properties

**Fig. 5:**
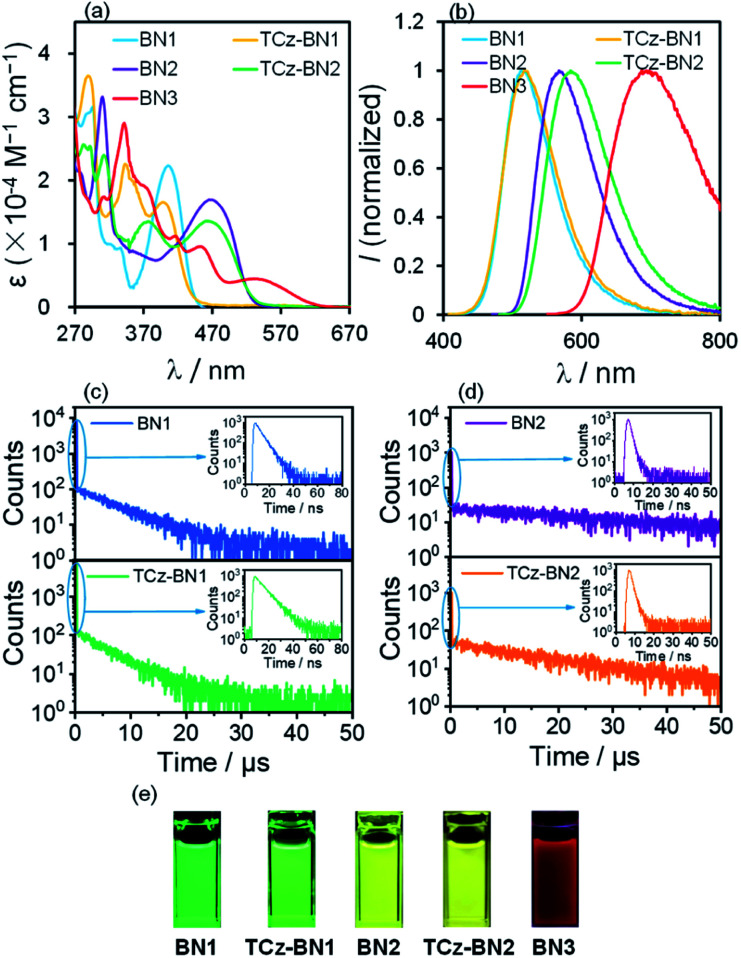
(a) UV-Vis absorption spectra and (b) emission spectra (excitation at 406 nm, 398 nm, 465 nm, 464 nm and 531 nm for BN1, TCz-BN1, BN2, TCz-BN2 and BN3, respectively) of all five compounds; (c and d) transient PL decay curves in THF solution under N_2_ conditions at 298 K; (e) photographs showing the emission colours of all five compounds.

The UV-Vis absorption and photoluminescence (PL) spectra of the five molecules were recorded in tetrahydrofuran (THF) at a concentration of 10^−5^ M. The corresponding spectra are given in Fig. 5 and the data are summarized in Table 1. The degree of overlap (*ϕ*_s_) was evaluated using the attachment detachment formalism in order to decipher the nature of the transitions, where a value of 0 (1) is of purely CT (LE, locally excited) character. A summary of the photophysical data, simulated spectra and high-intensity transitions can be found in Fig. S3.3–S3.9 and Tables S3.11–S3.15.‡

As shown in Fig. 5a, BN1 displays strong absorption bands from 270 to 470 nm. The absorption band at around 296 nm results from mainly the contribution of two excited states and can be ascribed to π → π* transitions involving a small degree of CT character from the two TCz units to the central pyridine unit (Fig. S3.5‡). The band around 406 nm is the SRCT band that is characteristic of MR-TADF emitters, involving both the TCz and the pyridine units. For TCz-BN1, three intense absorption bands are observed, with the lowest energy band at 398 nm assigned to a SRCT band. The second band at 343 nm is assigned to a similar transition; however, now there is also a larger contribution from the coupled carbazole. At 293 nm, π–π* from the fused Cz units and partial overlapping with the pyridine are observed in a similar fashion to BN1, and again this band is the result of transitions to two singlet states (Fig. S3.6‡). BN2 has two notable bands at 465 nm and 310 nm, with the former being a SRCT centred on the TCz and pyridine units. The higher energy band is made up of transitions to two nearly degenerate singlet states, with transitions occurring on separate TCz units, assigned to π–π* transitions of LE character (Fig. S3.7‡). The absorption spectrum of TCz-BN2 has three bands at 464 nm, 377 nm and 312 nm, with the lowest energy band assigned to TCz pyridine SRCT. The second band is associated with a similar transition primarily between the fused carbazole and pyridine; the peripheral carbazole had little influence on these two bands. The third and most intense band is composed of a transition from the peripheral and one fused TCz unit to the centre of the system around pyridine (Fig. S3.8‡). Four clear absorption bands of increasing *ε* at 531 nm, 452 nm, 370 nm and 342 nm are present in BN3. The nature of each of these bands was primarily assigned to mixed CT/LE transitions between the TCz-fused units and the pyridine (Fig. S3.9‡). We observe a consistent decrease in *ε* values going from BN1, BN2 and BN3, indicative of a progressive increase in CT character.

The decreasing LE character is accompanied by a red-shift in the emission, captured by both M062X and SCS-CC2 calculations. For BN3, the increase in CT character is reflected in a red shift of the low energy absorption band, whose tail extends to 603 nm. This observation is in line with the predicted stabilization of the S_1_ state from SCS-CC2 calculations (Fig. 3).

The PL properties of the compounds were investigated in deaerated THF at room temperature. In THF, BN1, TCz-BN1, BN2, and TCz-BN2 are bright emitters, reflected by their high PL quantum yields (*Φ*_PL_) (92% for BN1, 89% for TCz-BN1, 64% for BN2, and 61% for TCz-BN2). The change in *Φ*_PL_ is in line with the evolution of the oscillator strength through the series of compounds (see Tables S3.6–S3.10‡). BN1 and TCz-BN1 exhibit structureless emission profiles with *λ*_PL_ = 514 nm and 517 nm, respectively. The N^N^N chelating compounds BN2 and TCz-BN2 display broad emission bands with *λ*_PL_ = 567 nm and 584 nm, respectively. In contrast, BN3 shows very weak red emission with *λ*_PL_ = 694 nm and *Φ*_PL_ = 1%, which may be explained by the high nonradiative decay rate of S_1_ excitations due to the small band gap that most red emitters suffer from.^39^ The trend in emission energies is corroborated by SCS-CC2 calculations and is explained by the increase in CT character in the lowest singlet excited state, which is associated with a stabilized S_1_. It is clear that the chelating backbone modification not only leads to an effective emission tuning, but also significantly influences the PL quantum yield. A slight degree of positive solvatochromism was observed for these compounds (Fig. S4.1–S4.5‡), which is a hallmark of the SRCT character associated with MR-TADF compounds.^18,40^ Importantly, the evolution in solvatochromism is well captured by the calculation of *D*_CT_. Unlike most previously reported MR-TADF emitters, the emission spectra here are broad with the FWHM between 82 nm and 151 nm, much larger than those of conventional MR-TADF emitters where the FWHM is usually <50 nm.^18^ Large structural changes between the ground and excited states are responsible for the broad emission.^41^ We exclude excimer or aggregate emission as the origin of the broad emission as the emission remains broad in dilute solution. We also note that the orthogonal arrangement of the phenyl substituent on the boron centre should effectively inhibit excimer or aggregate formation (see Fig. S4.6‡). The singlet and triplet energies of BN1, TCz-BN1, BN2, and TCz-BN2 were inferred from the onsets of the fluorescence and phosphorescence spectra at 77 K (doped in the PMMA film at 5 wt%) (Fig. S4.7 and S4.8‡), respectively. These four compounds have small singlet–triplet energy gaps, Δ*E*_ST_, ranging from 0.16–0.20 eV, which are sufficiently small to ensure that the RISC from T_1_ to S_1_ would be operational at ambient temperatures. Thus, these compounds are expected to be potential TADF emitters. Due to the instrument detection limitation, the 77 K phosphorescence spectrum for BN3 was not obtained.

Variable temperature time-resolved photoluminescence spectra were recorded in THF to investigate the nature of the delayed fluorescence behavior of these compounds. As shown in Fig. 5, the PL decay curves of BN1, BN2 and their corresponding derivatives consist of a prompt fluorescence component (nanosecond-scale, *τ*_PF_) and a delayed emission component (microsecond-scale, *τ*_DF_), which is characteristic of TADF emitters. The *τ*_PF_ values are 4.7 ns, 6.7 ns, 1.2 ns and 1.2 ns, while the *τ*_DF_ values are 6.9 μs, 5.4 μs, 33.2 μs, and 18.1 μs for BN1, TCz-BN1, BN2 and TCz-BN2, respectively. With an increase in temperature, there is a clear increase in the emission intensity for all four compounds (Fig. S4.9–S.12‡). Both the emission intensity and delayed emission component of these compounds significantly decreased in an air-saturated solution (Fig. S4.13–S4.16‡), indicating that triplet excited states are involved in overall emission, a key characteristic of TADF. Notably, BN1 and TCz-BN1 display not only the shortest *τ*_DF_ but also the higher *Φ*_PL_ among these compounds, making them attractive candidates for high-performance OLEDs. However, no delayed fluorescence emission was observed for BN3, likely due to the large amount of non-radiative decay exemplified by the low *Φ*_PL_.

To further elucidate the TADF behavior of the four emitters, the photophysical rate constant of radiative decay (*k*_r_), intersystem crossing (*k*_ISC_), and reverse intersystem crossing (*k*_RISC_) in the doped films were estimated from the *Φ*_PL_ and the lifetimes of the prompt/delayed components (*τ*_PF_/*τ*_DF_).^42^ As shown in Tables 2 and S4.2,‡ the *k*_r_ and *k*_RISC_ of the emitters with a C^N^C chelating backbone (BN1 and TCz-BN1) are larger than those of molecules that contain a N^N^N chelating backbone (BN2 and TCz-BN2). More importantly, a significant enhancement of *k*_RISC_ was also observed with the introduction of a third TCz unit at the 4-position of the pyridine. The *k*_RISC_ values of TCz-BN1 and TCz-BN2 (4.67 × 10^5^ s^−1^ and 2.44 × 10^5^ s^−1^, respectively) are larger than those of BN1 and BN2 (2.90 × 10^5^ s^−1^ and 2.10 × 10^5^ s^−1^, respectively). Increased *k*_RISC_ with carbazole substitution has been reported for other MR-TADF emitters (Fig. S8.1 and Table S8.1‡). It is not clear at this stage why this substitution improves their triplet harvesting properties.

**Table 2:** TADF properties of BN1, TCz-BN1, BN2 and TCz-BN2 in the doped film at ambient temperature^a^

Compounds	*τ*_PF_ [ns]/*τ*_DF_ [μs]^b^	*Φ*_PF_/*Φ*_DF_^c^ [%]	*k*_r_ d [10^7^ s^−1^]	*k*_ISC_ e [10^7^ s^−1^]	*k*_RISC_ f [10^5^ s^−1^]
BN1	45.9/4.5	43/32	0.94	1.24	2.90
TCz-BN1	46.9/3.0	36/35	0.81	1.32	4.67
BN2	85.3/20.4	11/42	0.13	1.04	2.10
TCz-BN2	98.2/15.1	15/47	0.15	0.87	2.44

a Measured as 2 wt% for BN1 and TCz-BN1 and 5 wt% for BN2 and TCz-BN2-doped thin films in a mCBP host matrix.

b Emission lifetime for prompt (*τ*_PF_) and delayed (*τ*_DF_) fluorescence.

c Quantum yields for prompt (*Φ*_PF_) and delayed fluorescence (*Φ*_DF_), *Φ*_PF_ + *Φ*_DF_ = *Φ*_PL_.

d Rate constant of fluorescence radiative decay (S_1_ → S_0_): *k*_r_ = *Φ*_PF_/*τ*_PF_.

e Rate constant of ISC (S_1_ → T_1_): *k*_ISC_ = (1 − *Φ*_PF_)/*τ*_PF_.

f Rate constant of RISC (T_1_ → S_1_): *k*_RISC_ = *Φ*_DF_/*k*_ISC_*τ*_PF_*τ*_DF_*Φ*_PF_.

### Electroluminescence (EL) performance

Based on their promising photophysical properties and high thermal stability, C^N^C-chelating boron compounds (BN1 and TCz-BN1) and N^N^N-chelating boron compounds (BN2 and TCz-BN2) were employed as terminal emitters in hyperfluorescent OLED devices.^43^

**Fig. 6:**
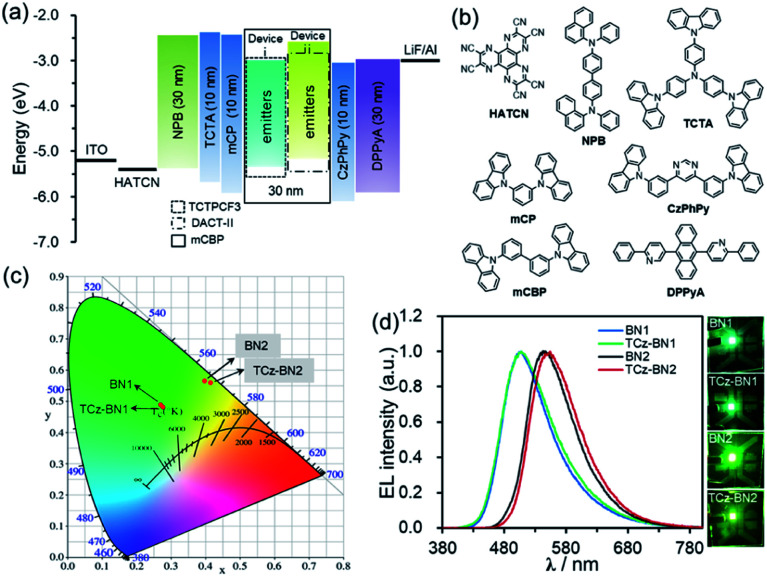
(a) Device structures and energy level diagrams of devices i and ii. (b) Molecular structures of the functional materials used in OLED devices. (c) The CIE 1931 chromaticity diagram and (d) EL spectra of devices recorded at 1000 cd m^−2^. Photographs showing the emission colours of the device at a luminance ≈1000 cd m^−2^.

The typical device structure used in our investigation is ITO/1,4,5,8,9,11-hexaazatriphenylene hexacarbonitrile (HATCN) (4.2 nm)/4,4′-*N*,*N*′-bis[*N*-(1-naphthyl)-*N*-phenylamino]biphenyl (NPB) (30 nm)/4,4′,4′′-tris(carbazol-9-yl)triphenylamine (TCTA) (10 nm)/9,9′-(1,3-phenylene)bis-9*H*-carbazole (mCP) (10 nm)/3,3′-bis(*N*-carbazolyl)-1,1′-biphenyl (mCBP) : 20% sensitizer : 2% or 5% terminal emitter (30 nm)/4,6-bis(3-(9*H*-carbazol-9-yl)phenyl)pyrimidine (CzPhPy) (10 nm)/9,10-bis(6-phenylpyridin-3-yl)anthracene (DPPyA) (30 nm)/LiF (1 nm)/Al (100 nm). The host material mCBP was selected because of its relatively wide HOMO–LUMO gap and suitably high triplet energy, which could be effective in facilitating the confinement of the exciton within the emitting layer. To evaluate the potential for using tridentate pincer-type organoboron compounds as an efficient emitter, the TADF materials, TCTPCF3 (ref. 44) and DACT-II,^45^ were selected as sensitizer assistant dopants as they provide good spectral overlap of their emission spectra with the absorption spectra of BN1 or TCz-BN1 and BN2 or TCz-BN2, respectively (Fig. S4.18–S4.21‡). Recent developments in hyperfluorescent OLEDs have shown that TADF-sensitized devices with conventional fluorescent,^46–48^ phosphorescent,^49^ TADF^2,50,51^ and MR-TADF^32,43,52–54^ OLEDs show remarkably improved performance. The detailed device configurations, energy diagrams, and molecular structures of the employed materials are shown in Fig. 6 and Table S4.1.‡

The optimized concentrations of the assistant dopant and terminal emitter are 20 wt% and either 2 wt% or 5 wt%, respectively. At the 2 wt% doping level, both BN1 and TCz-BN1-based OLEDs demonstrate structureless EL spectra peaking at 507 nm, producing green colour emission. The EL spectra of the devices based on BN2 and TCz-BN2 are red-shifted to yellow-green regions peaking at 547 and 554 nm, respectively. These EL spectra are in good agreement with the PL spectra of the corresponding doped host films (Fig. 6c, S4.18–S4.22 and Table S4.2‡) and point to an efficient and complete energy transfer from the sensitizing assistant dopant to the terminal emitter. Additionally, the EL spectra of the OLEDs measured at gradually increased operation voltages are nearly identical, which can be attributed to good charge balance within the device and effective exciton confinement.

**Fig. 7:**
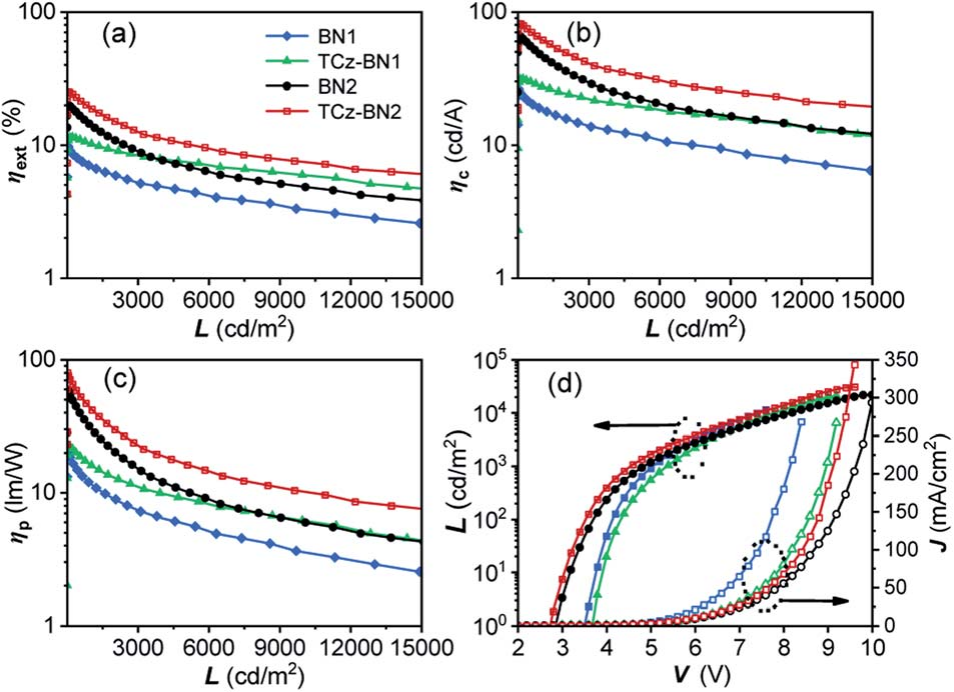
EL characteristics of OLED devices based on BN1, TCz-BN1, BN2 and TCz-BN2. (a) The external quantum efficiency (*η*_ext_), (b) current efficiency (*η*_c_) and (c) power efficiency (*η*_p_) *versus* luminance (*L*) curves for the devices; (d) luminance (*L*)–voltage (*V*)–current density (*J*) characteristics for the devices.

The current density–voltage–luminance (*J*–*V*–*L*) and external quantum efficiency (*η*_ext_) *versus* luminance plots are shown in Fig. 7, and the EL data are summarized in Table 3. The maximum external quantum efficiency and the maximum brightness were measured to be 9.9% and 18 180 cd m^−2^ (at 8.4 V) for 2 wt% of the BN1 doped device, and 11.5% and 20 952 cd m^−2^ (at 9.2 V) for 2 wt% of the TCz-BN1 doped device, respectively.

**Table 3:** Summary of the device performances

Emitter (doping concentration)	*λ*_EL_ a [nm]	*V*_on_ b [V]	*L*_max_/*V*^c^ [cd m^−2^/V]	*η*_c_ d [cd A^−1^]	*η*_p_ e [lm W^−1^]	*η*_ext_ f [%]	CIE (*x*, *y*)^a^
BN1 (2%)	507	3.5	18 180/8.4	26.6/21.6/19.1	22.0/14.8/11.9	9.9/8.0/7.0	(0.27, 0.49)
TCz-BN1 (2%)	507	3.6	20 952/9.2	31.3/29.8/27.5	22.2/18.7/16.0	11.5/11.0/10.2	(0.28, 0.48)
BN2 (5%)	547	2.9	21 576/10	66.1/55.7/44.9	59.0/39.7/28.2	19.9/16.7/13.5	(0.40, 0.57)
TCz-BN2 (5%)	554	2.7	30 708/9.6	81.8/70.1/61.2	79.7/52.4/41.8	25.1/21.4/18.7	(0.41, 0.56)

a Value taken at *L* = *ca.* 1000 cd m^−2^.

b Turn-on voltage at 1 cd m^−2^.

c Maximum luminescence and corresponding voltage.

d Maximum efficiency value, value at 500 and 1000 cd m^−2^ for current efficiency (*η*_c_).

e Maximum efficiency value, value at 500 and 1000 cd m^−2^ for power efficiency (*η*_p_).

f Maximum efficiency value, value at 500 and 1000 cd m^−2^ for external quantum efficiency (*η*_ext_).

The low turn-on voltages of 2.9 and 2.7 V and high maximum *η*_ext_ of 19.9% and 25.1% were achieved for the 5 wt% BN2 or TCz-BN2 based OLEDs, respectively, illustrating balanced hole/electron transport in the emitting layer. Notably, compared with the BN1 and BN2 doped devices, low efficiency roll-offs were observed for the TCz-BN1 and TCz-BN2 based devices, with efficiencies of 10.2% and 18.7% (*η*_ext_) at 1000 cd m^−2^, respectively. Considering the high PL quantum yield of the emitters, the relatively moderate device *η*_ext_ may be explained by the slower RISC rate from T_1_ to S_1_ and the charge carrier imbalance. The TCz-BN2 based OLED device also exhibited a high *η*_c_ and *η*_p_ of 81.8 cd A^−1^ and 79.7 lm W^−1^. Compared with BN1/BN2 based devices, the inclusion of the pendant TCz group at the 4-position of the pyridine significantly improved the efficiency of the TCz-BN1/TCz-BN2 based devices, revealing the great impact of the enhancement of *k*_RISC_ and the reduced delayed fluorescence lifetime of the emitters on the final performance of the devices. For comparison, the devices without the use of the assistant dopants TCTPCF3 or DACT-II in 2 wt% of BN1 and TCz-BN1 based devices or 5 wt% of BN2 and TCz-BN2 based devices were prepared. As shown in Fig. S5.7 and Table S5.3,‡ the dramatically decreased maximum *η*_ext_ values between 5.5% and 7.8% reveal that the sensitizers play critical roles in the efficient triplet excition utilization in the doped devices. It should be noted that the device based on TCz-BN2 exhibited not only the highest *η*_ext_ but also the lowest efficiency roll-off compared with the devices based on the published tetracoordinate boron TADF compounds (summarized in Table S5.4‡).

## Conclusions

In summary, we reported a novel class of tetracoordinate boron-containing MR-TADF emitters based on a C^N^C- or N^N^N-chelating pincer ligand. The emission colour of these materials can be effectively tuned from green to deep red *via* either replacement of the B–C covalent bonds with B–N covalent bonds or enlarging the π-conjugation in the tridentate chelate skeleton. These tetracoordinated boron-containing compounds showed small Δ*E*_ST_ and pronounced TADF properties. The introduction of a *tert*-butyl carbazole at the 4-position of the pyridine significantly enhanced the thermal stability and the reverse intersystem crossing rate. High-performance hyperfluorescent OLEDs have been achieved using TCz-BN1 and TCz-BN2 as the terminal emitters with a maximum *η*_ext_ of 11.5% for a green device and 25.1% for a yellow-green device, respectively.

## Data availability

The research data supporting this publication can be accessed at https://doi.org/10.17630/09321c5a-1c5c-47bd-9993-e23958081313.

## Author contributions

Guoyun Meng, Lijie Liu and Zhechang He conducted the compounds' synthesis work. Guoyun Meng also conducted the OLED fabrication and wrote the original draft of the manuscript. Xiang Wang conducted the single crystal analysis in this work. Tai Peng co-supervised Guoyun Meng and co-wrote the manuscript. Xiaodong Yin and Pangkuan Chen participated in the discussion of the photo-physical properties. Nan Wang co-supervised Guoyun Meng and Lijie Liu and co-wrote the manuscript. Suning Wang co-supervised Guoyun Meng and Lijie Liu and supervised Xiang Wang and Zhechang He. David Hall conducted the coupled cluster calculations and co-wrote the manuscript. Yoann Olivier co-supervised David Hall and co-wrote the manuscript. David Beljonne co-wrote the manuscript. Eli Zysman-Colman co-supervised David Hall and co-wrote the manuscript.

## Conflicts of interest

There are no conflicts to declare.

## Supplementary Material

SC-013-D1SC05692A-s001

SC-013-D1SC05692A-s002
