# Supplementary material for: Multi-resonant thermally activated delayed fluorescence emitters based on tetracoordinate boron-containing PAHs: colour tuning based on the nature of chelates
Source: Chem Sci. 2022 Jan 4;13(6):1665–74. doi: 10.1039/d1sc05692a (PMC8827120; doi:10.1039/d1sc05692a)
Supplement: SC-013-D1SC05692A-s001 [file SC-013-D1SC05692A-s001.pdf]

Electronic Supplementary Material (ESI) for Chemical Science.
This journal is © The Royal Society of Chemistry 2022

Multi-resonant Thermally Activated Delayed Fluorescence Emitters based on Tetracoordinate Boron-containing PAHs: Colour Tuning Based on the Nature of Chelate

Guoyun Meng, Lijie Liu, Zhechang He, David Hall, Xiang Wang, Tai Peng,* Xiaodong Yin, Pangkuan Chen, David Beljonne, Yoann Olivier, Eli Zysman-Colman,* Nan Wang* and Suning Wang

Table of contents

S1 Experimental Section

S2 Differential Scanning Calorimetry (DSC) and Electrochemical Analysis

S3 Crystal Structure

S4 Theoretical Calculation

S5 Photophysical Properties

S6 Organic Light Emitting Diode (OLED) Device Data

S7 ^{1}H NMR, ^{13}C NMR and ^{11}B NMR Data

S8 Literature study

S9 References

S1 Experimental Section

General

All reactions and manipulations were carried out in oven-dried flasks under an inert N_2 atmosphere using Schlenk techniques. Anhydrous solvents were used under an inert N_2 atmosphere. Materials were purchased from either Energy Chemical Co. or J&K Scientific Ltd. and used without further purification. Organic compounds for OLED-device fabrication were purchased from Jilin OLED Material Tech Co., Ltd, and used without further purification. Proton nuclear magnetic resonance (^{1}H NMR) and carbon nuclear magnetic resonance (^{13}C NMR) spectra were recorded on a Bruker Avance 400 MHz or 700 MHz spectrometers. Thermogravimetric analysis (TGA) and differential scanning calorimetry (DSC) measurements were performed on a Shimadzu DTG-60AH and Shimadzu DSC-60, respectively, under N_2 atmosphere with a heating rate of 10 °C min^{-1}. High-resolution mass spectra (HRMS) spectra were obtained using the Electrospray ionization (ESI) method with an Autoflex III mass spectrometer and a solariX spectrometer. MALDI-TOF-MS data was performed on a Shimadzu AXIMA Performance MALDI-TOF instrument in positive detection modes. Elemental analysis was obtained with a CE-440 Elemental Analyzer.

Photophysical property measurement

UV-visible absorption spectra were recorded at room temperature using a Cary 300 UV-Vis spectrophotometer. Photoluminescence (PL) spectra were recorded on an Edinburgh Instruments FLS980 spectrophotometer. PL decay curves were recorded on an Edinburgh Instruments FLS980 spectrophotometer equipped with 365 nm picosecond pulsed LEDs and analyzed using the Edinburgh Instruments F980 software. Temperature-dependent PL and time-resolved decay spectra were recorded under N_2 atmosphere using Edinburgh Instruments FLS980 spectrophotometer equipped with an Oxford Instruments liquid nitrogen cryostat. PL quantum yields (Φ_{PL}) of the samples were measured under N_2 atmosphere utilizing an integrating sphere of Hamamatsu QY spectrometer C11347-11.

Electrochemical analysis

Electrochemical measurements were conducted on an AUTOLAB-CV-75W analyzer with a scan rate of 100 mV s^{-1}. The electrochemical cell was a standard three-compartment cell composed of a Pt working electrode, a Pt auxiliary electrode, and a Pt wire reference electrode. All measurements were performed in anhydrous and nitrogen-saturated CH_2Cl_2 solutions with 0.10 M of *n*-Bu_4NPF_6 as the supporting electrolyte and 2.0 mM investigated compounds. The potentials are reported relative to the ferrocene/ferrocenium couple. The HOMO and LUMO levels were estimated from the peak maxima obtained from the differential pulse voltammetry (DPV). $E_{HOMO} = -[E_{ox} + 4.8]$ eV, $E_{LUMO} = -[E_{red} + 4.8]$ eV, $\Delta E = E_{LUMO} - E_{HOMO}$. Oxidation and reduction potentials are based on DPV measurements and are reported against the Fc/Fc^+ redox couple as internal standard.

Theoretical calculations

The calculations were performed with the Gaussian 16 suite[1] for the density functional theory (DFT) and with the Turbomole/7.4 package[2] or SCS-CC2 calculations. Ground state optimized structures were obtained using B3LYP [3], PBE0[4], LC-ωPBE[5], CAM-B3LYP[6], M062X[7], functionals each employing the 6-31G(d,p) basis set[8]. Excited state calculations were performed for each functionals at the dependent DFT (TD-DFT)[9] level as well as within the Tamm-Dancoff approximation (TDA-DFT)[9]. The attachment/detachment formalism was employed to calculate ϕs values for each of the excited states using the NANCY package[10]. Molecular orbitals were visualised using Gaussview 6.0 software[11]. Besides DFT calculations, we have investigated all compounds using Spin-component scaling coupled-cluster singles-and-doubles model (SCS-CC2)[12]. We first optimized the ground state using SCS-CC2/cc-pVDZ[13]. Vertical excited states of singlets[14] and triplets[15] were performed using the ground state optimized structure at the same level of theory. Difference density plots were used to visualize change in electronic density between the ground and excited state using the VESTA package[16].

Single Crystal Analysis

Single-crystal X-ray diffraction data were recorded on a Bruker D8 Venture X-ray single-crystal diffractometer using Mo Kα radiation (λ = 0.71073 Å) at 180 K for all compounds. Data were processed on a PC with the aid of the Bruker SHELXTL software package and corrected for absorption effects. All non-hydrogen atoms were refined anisotropically. The positions of hydrogen atoms were calculated and refined isotropically. Full crystallographic information in CIF format has been deposited at the Cambridge Crystallographic Data Center (CCDC) under deposition number 2048205 (**BN2**) and 2084998 (**BN3**). Due to the weak reflections and easily solvent lost, the structures of crystal **BN1** and **TCz-BN1** were not completed because of the limited amount of data. The crystal data are given here for information.

Device Fabrication and Measurement

All OLEDs were fabricated on glass substrates coated with a patterned transparent ITO (indium tin oxide) conductive layer. The ITO glasses were cleaned by successively sonicating in a detergent solution, distilled water, acetone, and isopropanol in an ultrasonic bath. Prior to use, each ITO glass was cleaned by rinsing with acetone and isopropanol. Subsequently, the surface of the ITO substrate was dried for 5 min in an oven at 393 K. After 10 min ozone plasma treatment, using a Harrick Plasma PDC-32G-2, 100W Plasma Cleaner, the substrates were loaded into a deposition chamber with a vacuum better than $< 5.0 \times 10^{-4}$ Pa during the evaporation process. All organic materials were thermally evaporated at a deposition rate of 0.2−0.3 Å s^{-1} using a multisource organic molecular vapor deposition system JD400C from Jiuda Vacuum Technology Co., Ltd. A LiF layer was deposited at a rate of 0.05 Å s^{-1} and the Al cathode at a rate of 3−5 Å s^{-1}. The thickness of each layer was characterized in advance on a Bruker Dektak XT surface profiler. The active area of the diode segments was 2×2 mm^2. The OLED characteristics of all fabricated devices were evaluated at room

temperature under nitrogen atmosphere inside a glovebox using a spectrophotometer PR-655, Photo Research with a computer-controlled programmable direct-current power supply Keithley model 2400 voltage-current source.

Synthesis and Characterization

Synthesis of compound 3,6-di-tert-butyl-9-(2,6-dibromopyridin-4-yl)-9H-carbazole

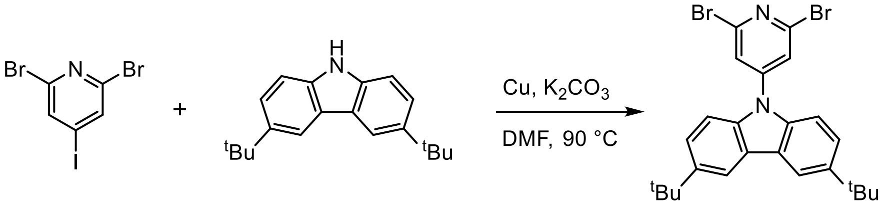

To a 100 mL Schlenk flask with a stir bar was added Cu powder (2.09 g, 33.0 mmol), 3,6-di-tert-butyl-9H-carbazole (4.0 g, 11.0 mmol, TCz), 2,6-dibromo-4-iodopyridine (3.02 g, 10.8 mmol), K_2CO_3 (4.56 g, 33.0 mmol), and DMF (50 mL). The resulting mixture were heated at 90 °C for 5 hours. After the solvent was removed under reduce pressure, the reaction mixture was purified by flash chromatography on silica gel with petroleum ether / CH_2Cl_2 (5:1, v/v) as eluent to give a white powder (3.89 g, 70%). ^{1}H NMR (400 MHz, $CDCl_3$, δ): 8.10 (s, 2H), 7.76 (s, 2H), 7.55–7.50 (m, 4H), 1.47 (s, 18H). ^{13}C NMR (100 MHz, $CDCl_3$, δ): 149.1, 145.5, 142.0, 137.3, 125.0, 124.6, 122.6, 116.9, 109.5, 35.0, 32.0. HRMS (ESI) calcd for $C_{25}H_{27}Br_2N_2$ $[M+H]^+$: m/z 513.0536. Found: 513.0536.

Synthesis of compound 1b/1c

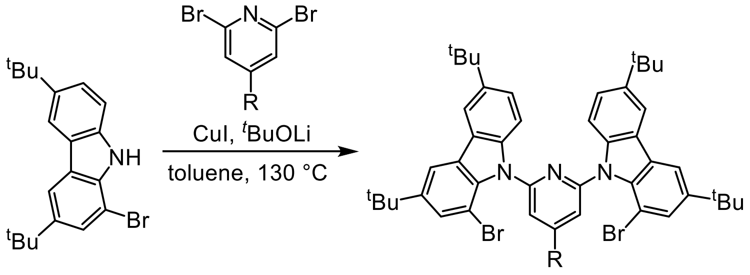

1a, 80%

R= H, 1b, 94%
R= TCz, 1c, 84%

*General procedure for **1b**:* 1-bromo-3,6-di-tert-butyl-9H-carbazole (**1a**) was prepared according to a modified literature procedure[17].Under nitrogen, 2,6-dibromopyridine (1.10 g, 4.64 mmol), **1a** (3.23 g, 9.29 mmol), CuI (18 mg, 0.186 mmol), ^{t}BuOLi (1.12 g, 13.9 mmol), 1-Methylimidazole (30 mg, 0.37 mmol) were added in a Schlenk flask. The flask was evacuated and backfilled with nitrogen three times before toluene (30 ml) was added under nitrogen, then the reaction mixture was warmed slowly and refluxed for 12 h. Then the reaction was quenched with methanol, after the removal of the solvent in vacuo, the residue was partitioned between CH_2Cl_2 and water, the aqueous layer was extracted with CH_2Cl_2. After the organic phase was dried over anhydrous $MgSO_4$, the solvent was removed in vacuo and the residue was purified by column

chromatography on silica gel with petroleum ether / CH_2Cl_2 (10:1, v/v) as eluent to give the final compound as a white powder (3.45 g, yield 94 %). ^{1}H NMR (400 MHz, $CDCl_3$, δ): 8.11 – 7.98 (m, 5H), 7.61 (d, *J* = 1.7 Hz, 2H), 7.49 – 7.37 (m, 6H), 1.45 (s, 18H), 1.43 (s, 18H). ^{13}C NMR (100 MHz, $CDCl_3$, δ): 150.8, 145.1, 144.2, 141.0, 139.0, 136.1, 128.6, 127.3, 124.9, 123.1, 116.1, 115.7, 110.6, 104.1, 34.8, 34.8, 31.9, 31.9. HRMS (ESI) calcd for $C_{45}H_{50}Br_2N_3$ $[M+H]^+$: m/z 792.2351. Found: 792.2337.

*Synthesis of **1c**:* Compound **1c** was synthesized using the same procedure of synthesis as that for compound **1b** as a white powder (4.05 g) in 84% yield. ^{1}H NMR (400 MHz, $CDCl_3$, δ): 8.08 (td, *J* = 5.3, 1.4 Hz, 6H), 7.81–7.78 (m, 4H), 7.65 (d, *J* = 1.7 Hz, 2H), 7.52–7.46 (m, 6H), 1.48–1.41 (m, 54H). ^{13}C NMR (101 MHz, $CDCl_3$, δ): 152.9, 145.5, 144.8, 144.5, 141.2, 137.9, 136.4, 128.7, 127.4, 125.2, 124.7, 124.4, 123.3, 116.7, 116.3, 116.0, 110.7, 109.9, 34.9, 32.1, 32.0, 31.9. MALDI-TOF calcd for $C_{65}H_{73}Br_2N_4$ $[M+H]^+$: m/z 1067.4196. Found: 1067.3749.

Synthesis of compound BN1 and TCz-BN1

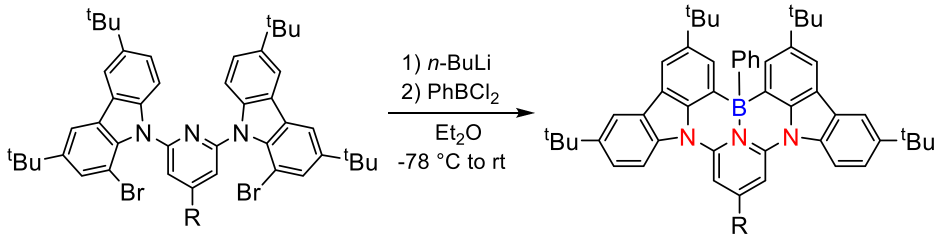

General procedure: **1b**/**1c** (0.148 mmol) and 15 mL of dry/degassed Et_2O were added to an oven-dried 50 mL Schlenk flask under N_2. The mixture was cooled to −78 °C and n-BuLi (0.31 mmol) was added dropwise. After stirring at the same temperature for 2 hours, $PhBCl_2$ (0.148 mmol, 1.0 eq.) was added dropwise into the lithiation flask and the completed reaction was allowed to slowly warm to room temperature over 18 hours. The desired products were extracted with CH_2Cl_2 (40 mL) and purified by column chromatography employing gradient elution (hexane : CH_2Cl_2, 6:1 → 2:1), affording **BN1** and **TCz-BN1** in moderate to high yields (60–82%).

***BN1**:* yellow powder, 87 mg, yield 82%. ^{1}H NMR (400 MHz, $CDCl_3$, δ): 8.22 (d, *J*= 1.7 Hz, 2H), 8.08 (d, *J*= 1.9 Hz, 2H), 7.89–7.75 (m, 8H), 7.49 (dd, *J*= 8.7, 2.0 Hz, 2H), 6.87 (s, 5H), 1.53 (s, 18H), 1.47 (s, 18H). ^{13}C NMR (176 MHz, $CDCl_3$, δ): 149.3, 147.6, 146.6, 141.3, 139.3, 131.3, 129.4, 126.9, 126.4, 125.0, 123.2, 121.5, 117.6, 114.9, 113.1, 104.7, 35.2, 34.9, 32.2, 31.7. ^{11}B NMR (225 MHz, $CDCl_3$, δ): −3.17. HRMS (ESI) m/z: calcd. for $C_{51}H_{55}BN_3$ $[M^+]$ 720.4484, found 720.4493. Elem. Anal. Calcd (%) $C_{51}H_{54}BN_3$: C, 85.10; H, 7.56; B, 1.50; N, 5.84; Found: C, 85.21; H, 7.51; B, 1.40; N, 5.88.

***TCz-BN1**:* yellow powder, 89 mg, yield 60%. ^{1}H NMR (400 MHz, CD_2Cl_2, δ): 8.22 (dd, *J* = 10.4, 1.7 Hz, 4H), 8.14 (d, *J* = 1.9 Hz, 2H), 8.09 (s, 2H), 7.92 (d, *J* = 1.7 Hz, 2H), 7.88 (d, *J* = 8.8 Hz, 2H), 7.76 (d, *J* = 8.7 Hz, 2H), 7.60 (dd, *J* = 8.7, 1.9 Hz, 2H),

7.48 (dd, J = 8.8, 2.0 Hz, 2H), 7.01–6.99 (m, 2H), 6.96–6.87 (m, 3H), 1.57 (s, 18H), 1.49 (s, 18H), 1.43 (s, 18H). ^{13}C NMR (101 MHz, CD_2Cl_2, δ): δ 151.0, 150.8, 148.3, 147.5, 145.6, 139.5, 138.0, 136.8, 131.1, 129.7, 127.6, 126.5, 125.5, 125.3, 125.0, 124.2, 122.1, 118.3, 117.4, 114.9, 113.6, 110.3, 101.0, 35.5, 35.2, 35.2, 32.3, 32.1, 31.8. ^{11}B NMR (128 MHz, CD_2Cl_2, δ): −4.96. High res. MALDI-MS (pos.) m/z: calcd. for $C_{71}H_{78}BN_4$ [M^+] 997.63141, found 997.63101. Elem. Anal. Calcd (%) $C_{71}H_{77}BN_4$: C, 85.51; H, 7.78; B, 1.08; N, 5.62; Found: C, 85.59; H, 7.85; B, 0.96; N, 5.56.

Synthesis of compound 2b/2c

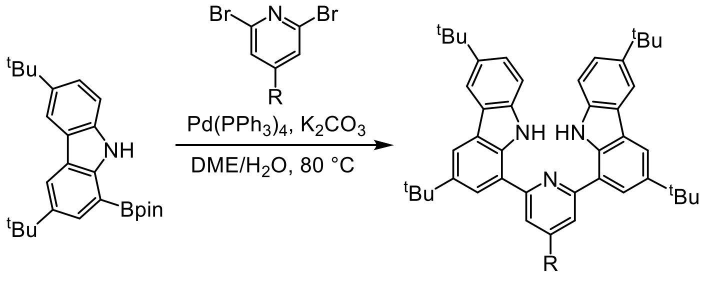

*General procedure for **2b**:* 3,6-di-tert-butyl-1-(4,4,5,5-tetramethyl-1,3,2-dioxaborolan-2-yl) -9H-carbazole (**2a**) was prepared according to a modified literature procedure[18]. In a 100 mL Schlenk flask, 2,6-dibromopyridine (0.2 g, 0.84 mmol) and **2a** (0.82 g, 1.69 mmol) were charged under nitrogen. After adding 20 mL dimethoxyethane and 5 mL K_2CO_3 aqueous solution, the mixture was degassed for 30 min. $Pd(PPh_3)_4$ (98 mg, 0.0085 mmol) was added, then the mixture was heated to 80 °C and stirred for 3 hours. The reactant was poured into brine and extracted by CH_2Cl_2 for three times. The organic phase was dried over $MgSO_4$ and the solvent was evaporated in vacuo. The crude product was purified by flash chromatography on silica gel with petroleum ether / CH_2Cl_2 (5:1, v/v) to give product as white powder (514 mg, 96 %). ^{1}H NMR (400 MHz, $CDCl_3$, δ): 10.28 (s, 2H), 8.28 (d, J = 1.7 Hz, 2H), 8.15 (d, J = 1.9 Hz, 2H), 8.08 (d, J = 1.8 Hz, 2H), 8.05–7.96 (m, 3H), 7.35–7.32 (m, 2H),, 6.95 (d, J = 8.5 Hz, 2H), 1.59 (s, 18H), 1.46 (s, 18H). ^{13}C NMR (101 MHz, $CDCl_3$, δ): 157.6, 142.5, 142.2, 138.5, 138.0, 136.6, 125.3, 124.2, 122.8, 121.9, 120.8, 119.1, 118.0, 116.2, 110.9, 35.1, 34.9, 32.3, 32.2. HRMS (ESI) calcd for $C_{45}H_{52}N_3$ $[M+H]^+$: m/z 634.4156. Found: 634.4146.

*Synthesis of **2c**:* Compound **2c** was synthesized using the same procedure of synthesis as that for compound **2b** as a white powder (0.70 g) in 91% yield. ^{1}H NMR (400 MHz, $CDCl_3$, δ):10.32 (s, 2H), 8.33 (d, J = 1.7 Hz, 2H), 8.24 (d, J = 1.9 Hz, 2H), 8.19 (s, 4H), 8.04 (d, J = 1.8 Hz, 2H), 7.67 (d, J = 8.7 Hz, 2H), 7.57 (dd, J = 8.7, 1.9 Hz, 2H), 7.36 (dd, J = 8.6, 1.9 Hz, 2H), 6.98 (d, J = 8.5 Hz, 2H), 1.56 (s, 18H), 1.52 (s, 18H), 1.48 (s, 18H). ^{13}C NMR (101 MHz, $CDCl_3$, δ): 159.9, 147.9, 144.3, 142.7, 142.4, 138.5, 138.4, 136.7, 125.5, 124.4, 124.3, 122.8, 121.9, 120.5, 118.6, 116.8, 116.4, 115.8, 110.9, 109.6, 35.1, 35.0, 34.9, 32.3, 32.2, 32.1. MALDI-TOF calcd for $C_{65}H_{75}N_4$ $[M+H]^+$: m/z 911.5986. Found: 911.5803.

Synthesis of compound BN2/TCz-BN2

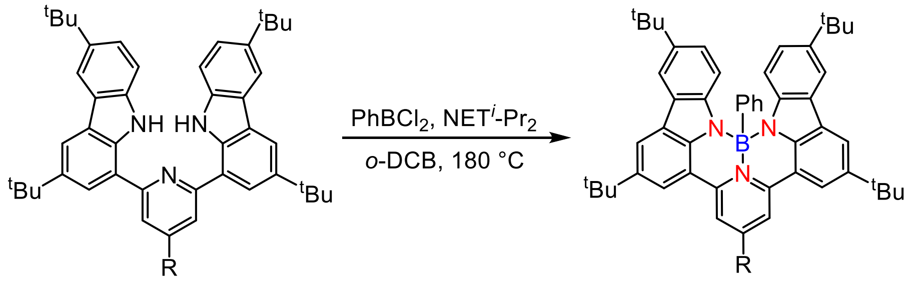

R= H, 2b
R= TCz, 2c

R= H, **BN2**, 89%
R= TCz, **TCz-BN2**, 50%

*General procedure for **BN2**:* To a solution of **2b** (0.4 g, 0.63 mmol) in o-dichlorobenzene (20 mL) under nitrogen was added *N*,*N*-diisopropylethylamine (NEt^{i}-Pr$_2$, 0.163 g, 1.26 mmol) and dichlorophenylborane (0.11 g, 0.69 mmol). The reaction mixture was heated to 180 °C for 24 h. After cooling to the room temperature, the solvent was evaporated through reduced pressure distillation. The residue was purified by flash chromatography on silica gel with petroleum ether / CH_2Cl_2 (5:1, v/v) to give product as orange powder (514 mg) in 89% yield. ^{1}H NMR (400 MHz, CD_2Cl_2, δ): 8.39 (d, *J* = 1.4 Hz, 2H), 8.36–8.34 (m, 1H), 8.21–8.20 (m, 2H), 8.15 (d, *J* = 1.4 Hz, 1H), 8.08 (t, *J* = 7.9 Hz, 1H), 8.01–7.99 (m, 1H), 7.95 (d, *J* = 1.4 Hz, 1H), 7.20 (dd, *J* = 8.7, 2.0 Hz, 1H), 7.16 (dd, *J* = 8.8, 2.1 Hz, 1H), 6.89–6.81 (m, 3H), 6.73 (t, *J* = 7.6 Hz, 2H), 6.17 (d, *J* = 6.9 Hz, 2H), 1.59 (s, 9H), 1.53 (s, 9H), 1.44 (s, 18H). ^{13}C NMR (101 MHz, CD_2Cl_2, δ): 153.6, 151.1, 145.5, 145.0, 143.7, 143.6, 143.5, 143.1, 140.6, 140.0, 132.6, 127.9, 127.6, 127.1, 126.5, 126.5, 125.8, 124.7, 124.1, 122.4, 122.2, 121.1, 119.8, 119.2, 118.9, 118.1, 117.7, 116.9, 113.2, 36.4, 36.4, 35.9, 35.9, 33.3, 33.1. ^{11}B NMR (128 MHz, CD_2Cl_2, δ): 4.66. HRMS (ESI) m/z: calcd. for $C_{51}H_{55}BN_3$ [M^{+}] 720.4484, found 720.4484. Elem. Anal. Calcd (%) $C_{51}H_{54}BN_3$: C, 85.10; H, 7.56; B, 1.50; N, 5.84; Found: C, 85.18; H, 7.46; B, 1.39; N, 5.98.

*Synthesis of **TCz-BN2**:* Compound **TCz-BN2** was synthesized using the same procedure of synthesis as that for compound **BN2** as a red powder (220 mg) in 50% yield. ^{1}H NMR (400 MHz, CD_2Cl_2, δ): 8.59 (d, *J* = 1.9 Hz, 1H), 8.43–8.42 (m, 2H), 8.23–8.22 (m, 5H), 8.10 (d, *J* = 1.3 Hz, 1H), 7.92 (d, *J* = 1.2 Hz, 1H), 7.78 (d, *J* = 8.7 Hz, 2H), 7.60 (dd, *J* = 8.7, 1.9 Hz, 2H), 7.23 (dd, *J* = 8.7, 2.0 Hz, 1H), 7.19 (dd, *J* = 8.8, 2.0 Hz, 1H), 6.94–6.87 (m, 3H), 6.82–6.79 (m, 2H), 6.31 (d, *J* = 6.6 Hz, 2H), 1.57 (s, 9H), 1.50 (s, 27H), 1.45 (s, 18H). ^{13}C NMR (101 MHz, CD_2Cl_2, δ): 155.5, 152.9, 149.9, 146.9, 145.4, 145.1, 143.8, 143.8, 143.7, 143.5, 143.2, 140.2, 138.9, 132.8, 128.1, 127.7, 127.3, 126.7, 126.4, 126.0, 125.8, 124.8, 124.2, 122.8, 122.6, 119.6, 119.2, 118.4, 118.1, 117.7, 117.4, 116.9, 116.9, 116.1, 113.7, 113.1, 111.3, 36.4, 36.3, 36.2, 36.0, 35.9, 33.3, 33.1, 33.0. ^{11}B NMR (128 MHz, CD_2Cl_2, δ): 4.29. HRMS (ESI) m/z: calcd. for $C_{71}H_{78}BN_4$ [M^{+}] 997.6314, found 997.6359. Elem. Anal. Calcd (%) $C_{71}H_{77}BN_4$: C, 85.51; H, 7.78; B, 1.08; N, 5.62; Found: C, 85.56; H, 7.70; B, 0.95; N, 5.78.

Synthesis of compound BN3

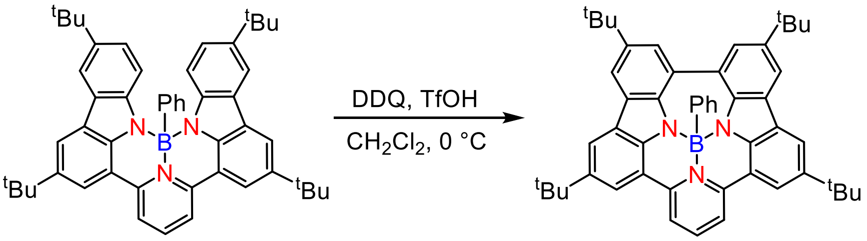

BN2 **BN3**, 68%

*Synthesis of **BN3**:* To a solution of compound **BN2** (115 mg, 0.16 mmol) in dichloromethane (35 mL) was added 2,3-dichloro-5,6-dicyano-1,4-benzoquinone (91 mg, 0.40 mmol), after stirring for 5 min, triflic acid (1.76 mL) was added under argon at 0 °C. Then the mixture was stirred for 20 min at the same temperature. After quenching with triethylamine, the reaction mixture was concentrated under reduced pressure. Purification of the crude product by column chromatography afforded the product **BN3** as a red solid (78 mg) in 68% yield. ^{1}H NMR (400 MHz, CD_2Cl_2, δ): 8.47–7.94 (m, 11H), 6.80 (t, J = 7.1 Hz, 1H), 6.72–6.68 (m, 2H), 6.48 (d, J = 7.3 Hz, 2H), 1.58 (s, 18H), 1.53 (s, 18H). ^{11}B NMR (128 MHz, CD_2Cl_2, δ): 4.10. HRMS (ESI) m/z: calcd. for $C_{51}H_{53}BN_3$ [M$^+$] 718.4327, found 718.4381. Elem. Anal. Calcd (%) $C_{51}H_{52}BN_3$: C, 85.34; H, 7.30; B, 1.51; N, 5.85; Found: C, 85.45; H, 7.42; B, 1.33; N, 5.80.

S2 Differential Scanning Calorimetry (DSC) and Electrochemical Analysis

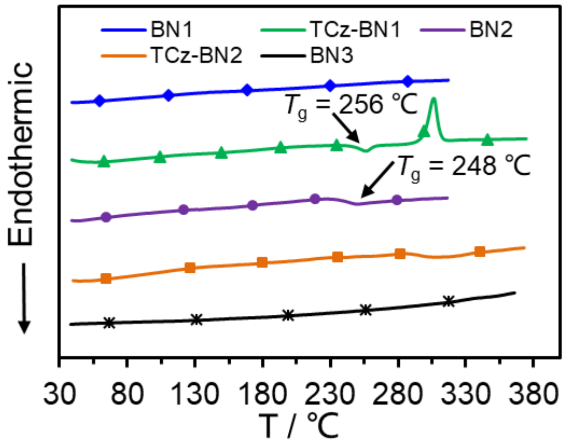

Figure S1.1 DSC plot of **BN1**, **TCz-BN1**, **BN2**, **TCz-BN2** and **BN3**. The samples were heated under a nitrogen atmosphere at a rate of 10 °C/min.

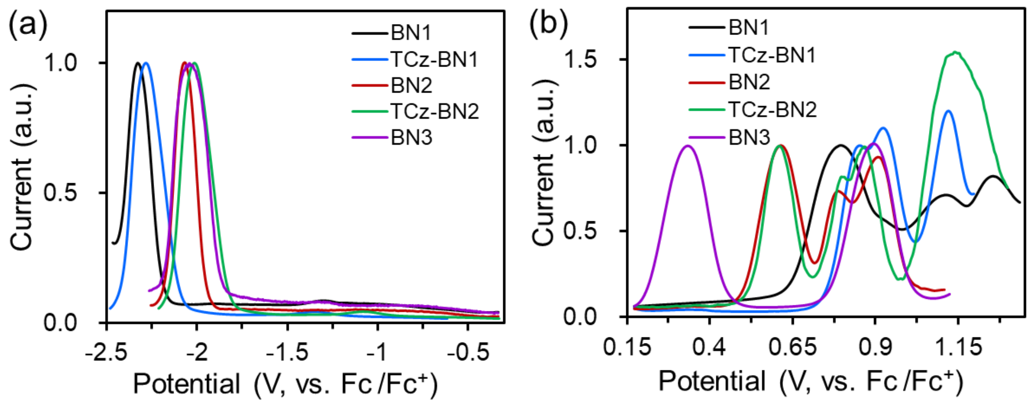

Figure S1.2 Differential Pulse Voltammetry (DPV) diagrams of **BN1**, **TCz-BN1**, **BN2**, **TCz-BN2**, and **BN3** showing the a) reduction and b) oxidation waves using *n*-Bu_4NPF_6 (0.1 M) as the electrolyte and a scan rate = 100 mV/s, recorded in CH_2Cl_2.

Table S1.1 HOMO and LUMO energies from electrochemical data.[a]

	E_{red}^{CV} (V)	E_{red}^{DPV} (V)	E_{ox}^{CV} (V)	E_{ox}^{DPV} (V)	$E_{HOMO/LUMO}$[b] (eV)	ΔE_g[c] (eV)
**BN1**	−2.37	−2.33	+0.81, +1.12, +1.26	+0.80, +1.11, +1.25	−5.60/−2.47	3.13
**TCz-BN1**	−2.33	−2.28	+0.88, +1.08	+0.86, +0.92, +1.12	−5.66/−2.52	3.14
**BN2**	−2.07	−2.06	+0.62, +0.80, +0.91	+0.61, +0.79, +0.91	−5.41/−2.74	2.67
**TCz-BN2**	−2.04	−2.01	+0.65, +0.81, +0.90, +1.14	+0.61, +0.79, +0.87, +1.13	−5.41/−2.79	2.62
**BN3**	−2.02	−2.04	+0.33, +0.87	+0.33, +0.89	−5.13/−2.76	2.37

[a] The oxidation and reduction potentials were recorded in CH_2Cl_2 solution using $[NBu_4]PF_6$ (0.1 M in solvents) as the electrolyte with a scan rate of 100 mV/s, respectively, and the Fc/Fc^+ was used as an internal standard; [b] Measured from the first reduction/oxidation peak positions in DPV diagrams, $E_{LUMO}/E_{HOMO} = -(4.8 + E_{red}/E_{ox})$; [c] $\Delta E_g = E_{LUMO} - E_{HOMO}$.

S3 Crystal Structure

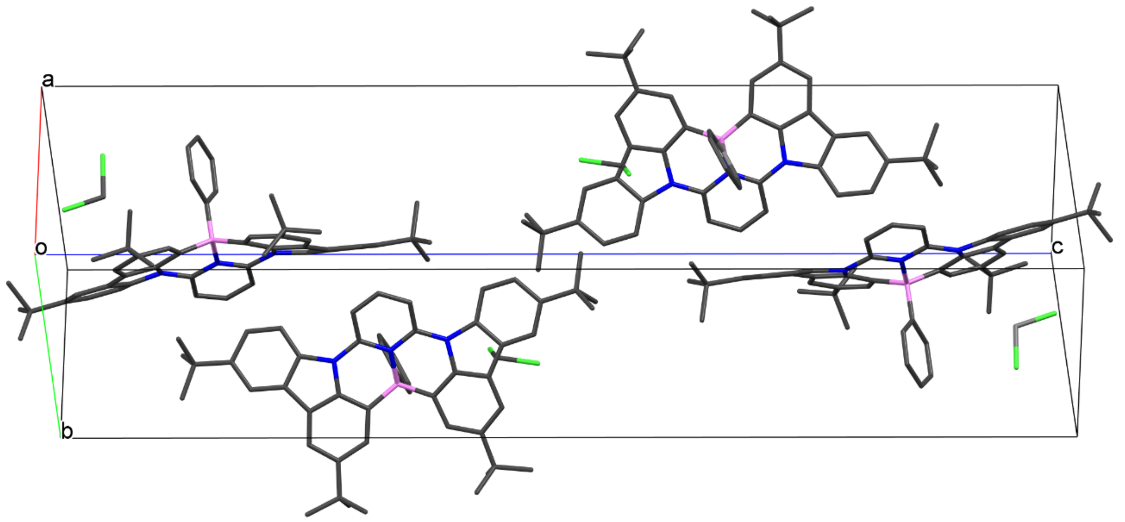

Figure S2.1 Arrangement of **BN1** molecules in a primitive unit cell.

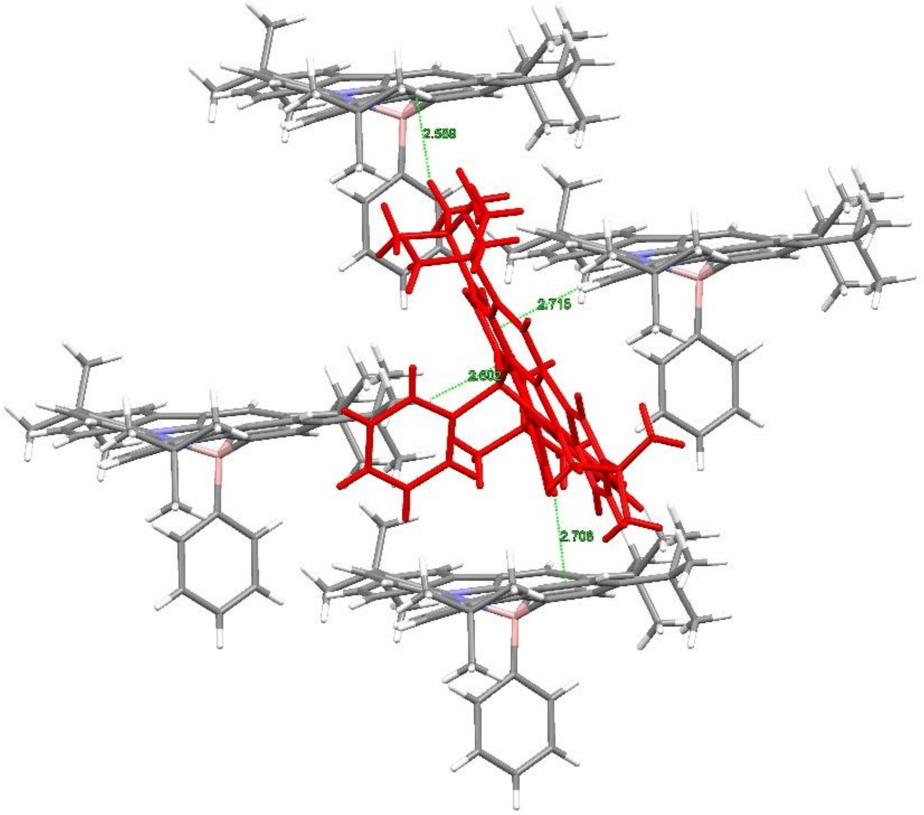

Figure S2.2 C-H···π interactions of **BN1** molecules.

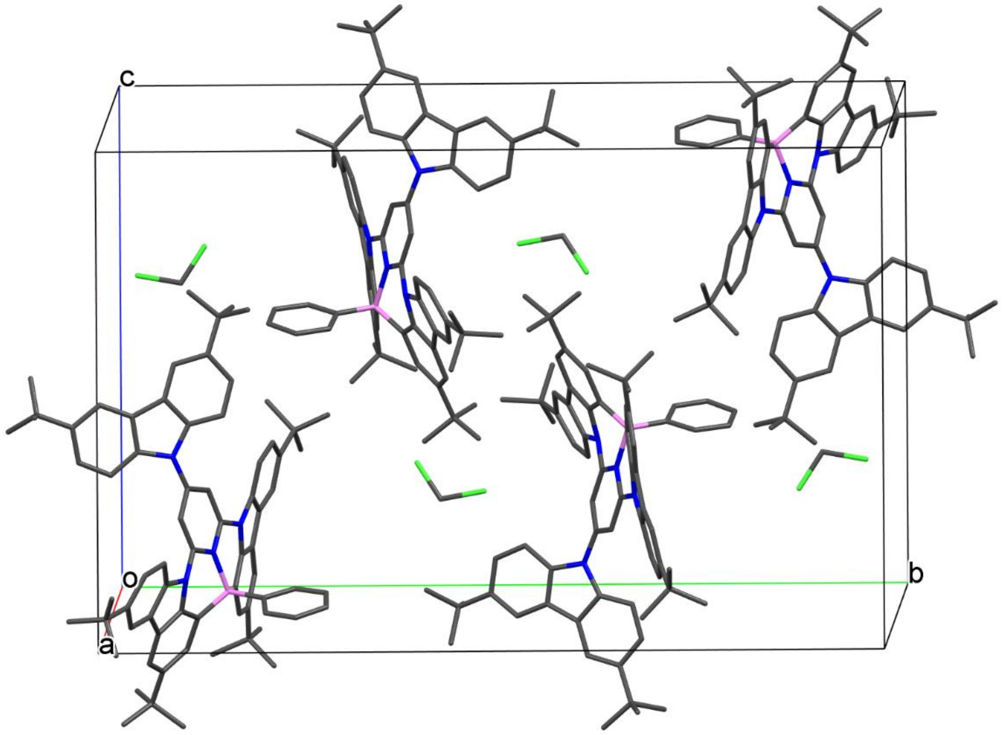

Figure S2.3 Arrangement of **TCz-BN1** molecules in a primitive unit cell.

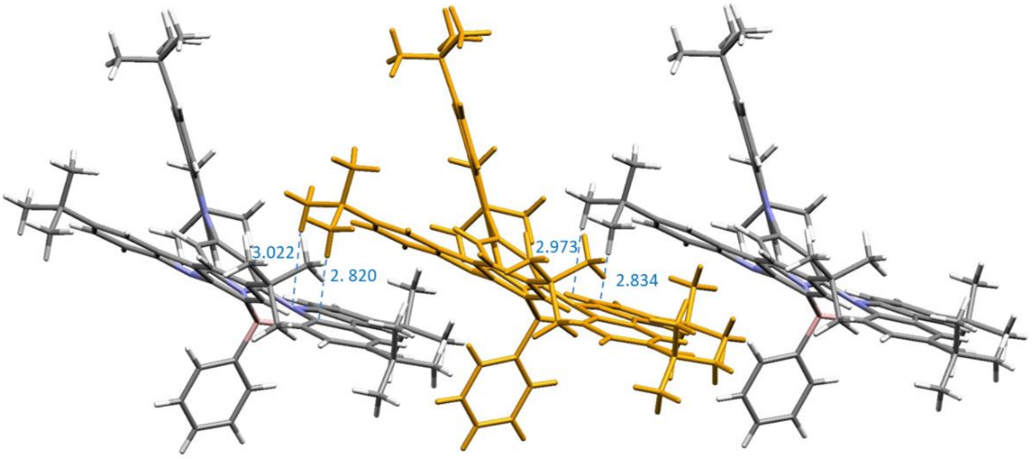

Figure S2.4 The intermolecular interactions of **TCz-BN1**.

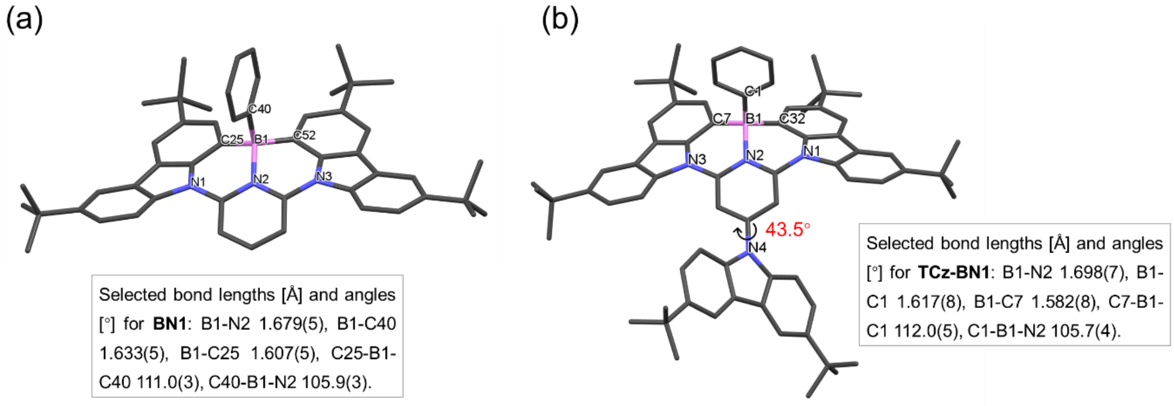

Figure S2.5 The selected bond lengths [Å] and angles [°] for (a) **BN1** and (b) **TCz-BN1**.

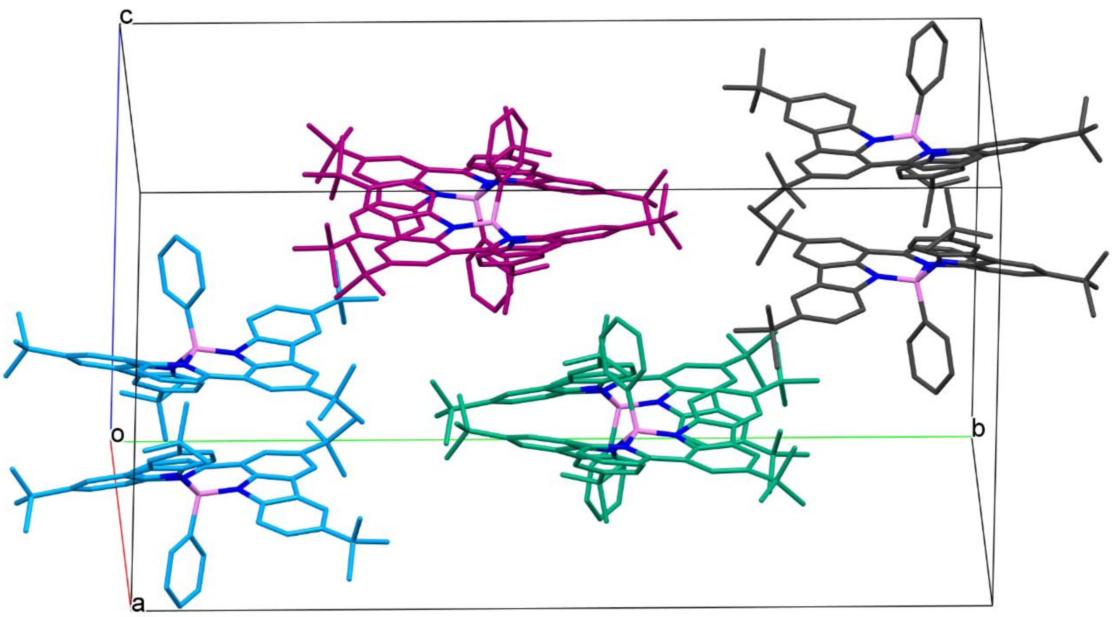

Figure S2.6 Arrangement of **BN2** molecules in a primitive unit cell.

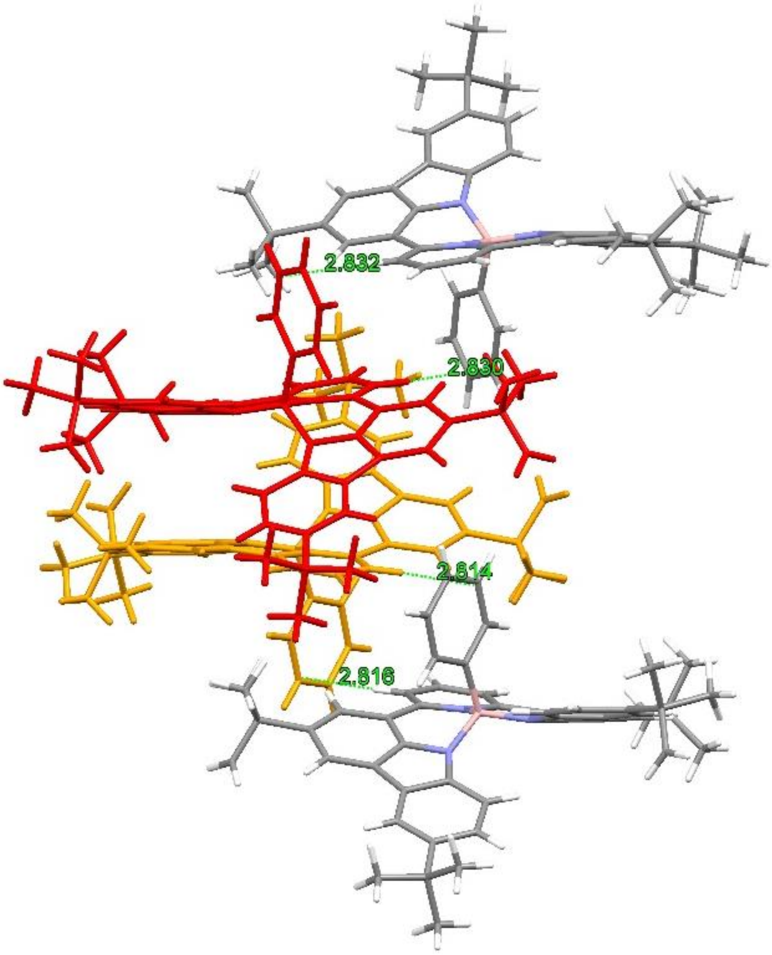

Figure S2.7 The intermolecular interactions of **BN2**.

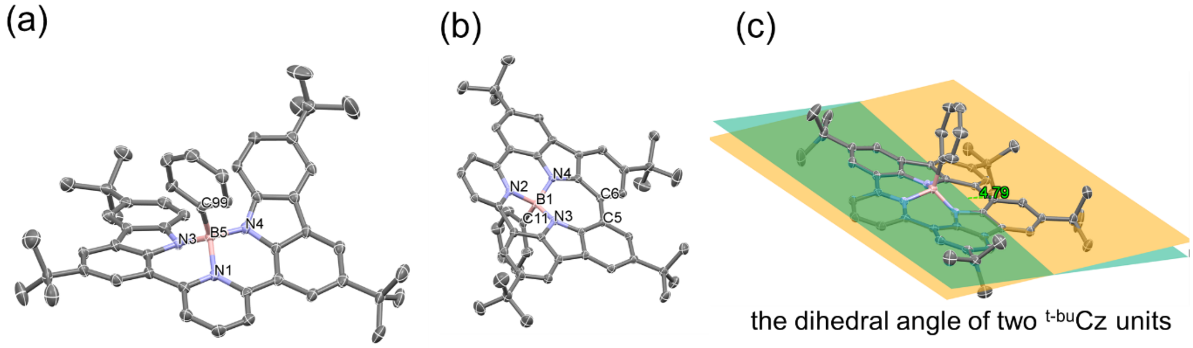

Selected bond lengths [Å] and angles [°] for **BN2**: B5-N1 1.650(5), B5-N3 1.546(5), B5-N4 1.518(4), B5-C99 1.603(5), C99-B5-N4 109.4(2), N3-B5-N1 104.8(2).

Selected bond lengths [Å] and angles [°] for **BN3**: B1-N2 1.636(2), B1-N3 1.503(2), B1-N4 1.517(2), B1-C11 1.624(2), C5-C6 1.497(2), C11-B1-N4 113.9(13), N3-B1-N2 107.2(12).

Figure S2.8 The selected bond lengths [Å] and angles [°] for (a) **BN2** and (b) **BN3**; (c) The dihedral angle of two $^{t\text{-}bu}$Cz units in crystal **BN3**.

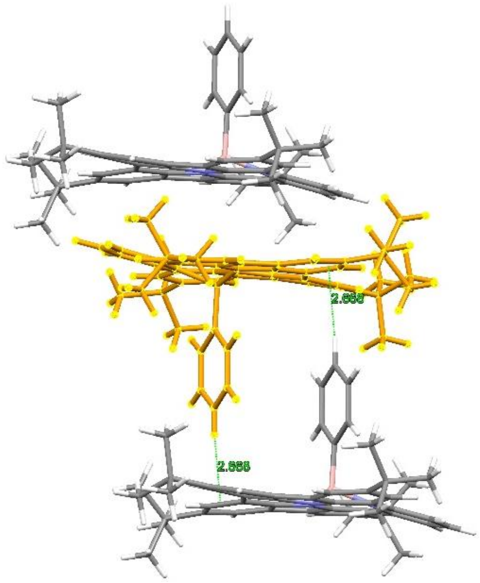

Figure S2.9 The intermolecular interactions of BN3.

S4 Theoretical Calculation

Table S3.1 S_1 and T_1 energies as well as ΔE_{ST} computed at the SCS-CC2/cc-PVDZ and TD(A)-DFT/6-31g(d,p) levels of theory for **BN1**. Comparison between computed and experimental $\boldsymbol{\Delta E_{ST}}$ is provided. .

Method	S_1 / eV	T_1 / eV	ΔE_{ST} / eV	Deviation from $\Delta E_{ST\text{-}exp}$ [a]
TD-CAM-B3LYP	3.64	2.96	0.67	0.47
TDA-CAM-B3LYP	3.72	3.10	0.62	0.42
TD-LC-ωPBE	3.94	2.63	1.31	1.11
TDA-LC-ωPBE	4.06	3.28	0.78	0.58
TD-B3LYP	3.09	2.70	0.39	0.19
TDA-B3LYPA	3.14	2.73	0.41	0.21
TD-PBE0	3.22	2.77	0.45	0.25
TDA-PBE0	3.27	2.82	0.46	0.26
TD-M062X	3.61	3.10	0.51	0.31
TDA-M062X	3.69	3.15	0.54	0.34
SCS-CC2	3.37	3.30	0.07	0.13

[a]Absolute value for ΔE_{ST}(experimental in 5 wt% PMMA) – ΔE_{ST}(calculated)

Table S3.2 S_1 and T_1 energies as well as ΔE_{ST} computed at the SCS-CC2/cc-PVDZ and TD(A)-DFT/6-31g(d,p) levels of theory for **CzBN1**. Comparison between computed and experimental $\boldsymbol{\Delta E_{ST}}$ is provided..

Method	S_1 / eV	T_1 / eV	ΔE_{ST} / eV	Deviation from $\Delta E_{ST\text{-}exp}$ [a]
TD-CAM-B3LYP	3.64	2.96	0.68	0.52
TDA-CAM-B3LYP	3.71	3.17	0.54	0.38

TD-LC-ωPBE	3.95	2.63	1.33	1.17
TDA-LC-ωPBE	4.06	3.36	0.70	0.54
TD-B3LYP	3.06	2.72	0.34	0.18
TDA-B3LYPA	3.11	2.75	0.36	0.20
TD-PBE0	3.19	2.79	0.39	0.23
TDA-PBE0	3.24	2.84	0.40	0.24
TD-M062X	3.60	3.15	0.45	0.29
TDA-M062X	3.67	3.19	0.48	0.32
SCS-CC2	3.32	3.27	0.05	0.09

[a]Absolute value for ΔE_{ST}(experimental in 5 wt% PMMA) – ΔE_{ST}(calculated)

Table S3.3 S_1 and T_1 energies as well as ΔE_{ST} computed at the SCS-CC2/cc-PVDZ and TD(A)-DFT/6-31g(d,p) levels of theory for **BN2**. Comparison between computed and experimental $\boldsymbol{\Delta E_{ST}}$ is provided.

Method	S_1 / eV	T_1 / eV	ΔE_{ST} / eV	Deviation from $\Delta E_{ST\text{-}exp}$[a]
TD-CAM-B3LYP	3.04	2.44	0.60	0.41
TDA-CAM-B3LYP	3.12	2.60	0.51	0.32
TD-LC-ωPBE	3.38	2.28	1.11	0.92
TDA-LC-ωPBE	3.50	2.82	0.68	0.49
TD-B3LYP	2.49	2.17	0.33	0.14
TDA-B3LYPA	2.54	2.20	0.34	0.15
TD-PBE0	2.63	2.23	0.39	0.20
TDA-PBE0	2.68	2.29	0.39	0.20
TD-M062X	3.04	2.61	0.44	0.25
TDA-M062X	3.12	2.67	0.45	0.26
SCS-CC2	2.80	2.72	0.08	0.09

[a]Absolute value for ΔE_{ST}(experimental in 5 wt% PMMA) – ΔE_{ST}(calculated).

Table S3.4 S_1 and T_1 energies as well as ΔE_{ST} computed at the SCS-CC2/cc-PVDZ and TD(A)-DFT/6-31g(d,p) levels of theory for **CzBN2**. Comparison between computed and experimental $\boldsymbol{\Delta E_{ST}}$ is provided.

Method	S_1 / eV	T_1 / eV	ΔE_{ST} / eV	Deviation from $\Delta E_{ST\text{-}exp}$[a]
TD-CAM-B3LYP	3.06	2.48	0.58	0.41
TDA-CAM-B3LYP	3.13	2.66	0.47	0.30
TD-LC-ωPBE	3.41	2.32	1.09	0.92
TDA-LC-ωPBE	3.52	2.87	0.65	0.48
TD-B3LYP	2.47	2.19	0.28	0.11
TDA-B3LYPA	2.50	2.22	0.29	0.12
TD-PBE0	2.61	2.26	0.36	0.19
TDA-PBE0	2.65	2.32	0.34	0.17
TD-M062X	3.06	2.66	0.39	0.28
TDA-M062X	3.12	2.72	0.41	0.30

SCS-CC2	2.76	2.70	0.06	0.05

[a]Absolute value for ΔE_{ST}(experimental in 5 wt% PMMA) – ΔE_{ST}(calculated).

Table S3.5 S_1 and T_1 energies as well as ΔE_{ST} computed at the SCS-CC2/cc-PVDZ and TD(A)-DFT/6-31g(d,p) levels of theory for **BN3**.

**Method**	**S_1 / eV**	**T_1 / eV**	**ΔE_{ST} / eV**
TD-CAM-B3LYP	2.74	2.31	0.43
TDA-CAM-B3LYP	2.79	2.44	0.35
TD-LC-ωPBE	3.19	2.16	1.03
TDA-LC-ωPBE	3.28	2.72	0.57
TD-B3LYP	2.05	1.89	0.16
TDA-B3LYPA	2.08	1.91	0.16
TD-PBE0	2.20	1.99	0.21
TDA-PBE0	2.23	2.03	0.20
TD-M062X	2.74	2.45	0.29
TDA-M062X	2.79	2.50	0.29
SCS-CC2	2.54	2.51	0.03

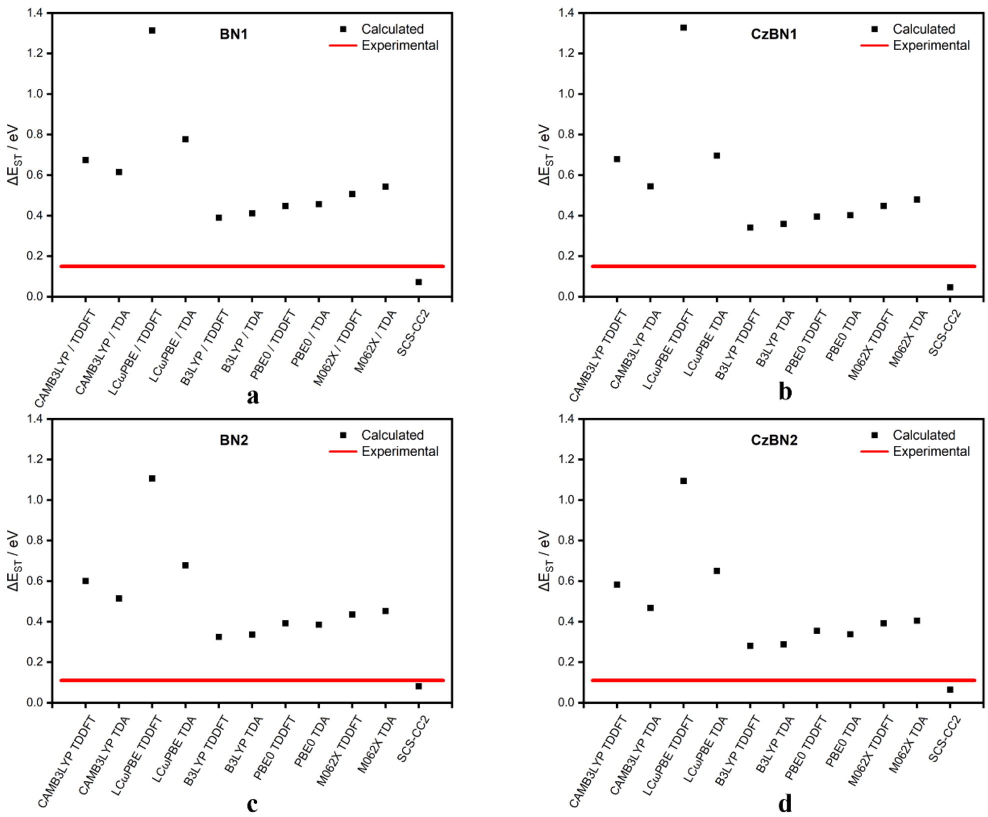

Figure S3.1 ΔE_{ST} calculated using various DFT functionals and SCS-CC2 compared to experimental ΔE_{ST} calculated in 5 wt% PMMA.

Table S3.6 Excited state energies and the corresponding charge transfer metrics for **BN1** as obtained at the SCS-CC2/cc-pVDZ level of theory.

**Excited**	**Energy**	***f***	**Charge**	**Distance**	**Single Electron transition**

State	/ eV		Transferred	charge transferred / Å	
T_1	3.30		0.64	1.31	H -> L 78%, H-8 -> L, 7% H - > L+2 3%
T_2	3.49		0.32	1.02	H-1 -> L+1 42% H-1 -> L 20% H -> L+1 5% H-1 -> L+3 3% H-1 -> L+2 3% H-6 -> L+3 2%
S_1	3.37	0.45	0.67	1.29	H -> L 82%, H-8 -> L 5%
S_2	4.00	0.06	0.51	1.96	H-3 -> L 34%, H -> L+1 29%, H-1-> L 6%, H-4 -> L 6%, H-1 -> L+5 2%, H-3 ->L+2 2%, H-2 -> L 2%

f is oscillator strength

Table S3.7 Excited state energies and the corresponding charge transfer metrics for **CzBN1** as obtained at the SCS-CC2/cc-pVDZ level of theory.

**Excited State**	**Energy / eV**	***f***	**Charge Transferred**	**Distance charge transferred / Å**	**Single Electron transition**
T_1	3.27		0.33	1.20	H -> L 79%, H-11 -> L 6%, H-1 -> L 2%
T_2	3.47		0.33	1.20	H-1 -> L+2 41%, H-1 -> L 17%, H -> L+2 7%, H-1-> L+4 6%, H-3 -> L+2 2%, H-9 -> L+4 2%, H-1 -> L +3 2%
S_1	3.32	0.37	0.68	1.77	H -> L 81%, H-11 -> L 4%, H-1 -> L 2%
S_2	3.86	0.33	0.65	1.22	H-3 -> L 48% H -> L+2 13%, H-6 -> L 9%, H-1 -> L 6%, H-2 -> L 3%

f is oscillator strength

Table S3.8 Excited state data for **BN2** using SCS-CC2 / cc-pVDZ.

**Excited State**	**Energy / eV**	***f***	**Charge Transferred**	**Distance charge transferred / Å**	**Single Electron transition**
T_1	2.72		0.62	2.06	H -> L 52%, H -> L+1 15%, H-1 -> L 12%, H-1 -> L+1 8%, H-7 -> L 2%, H-7 -> L+1 2%

T_2	2.74		0.60	2.27	H-1 -> L 29%, H -> L+1 27%, H -> L 23%, H-1 -> L+1 6% H-7 -> L 3%, H-6 -> L++1 2%, H-6 -> L 2%
S_1	2.80	0.20	0.67	2.51	H -> L 72%, H-1 -> L+1 16%, H-3 -> L 2%
S_2	2.97	0.16	0.72	3.05	H-1 -> L 73%, H -> L+1 15%, H-7 -> L 2%

f is oscillator strength

Table S3.9 Excited state energies and the corresponding charge transfer metrics for **CzBN2** as obtained at the SCS-CC2/cc-pVDZ level of theory.

Excited State	Energy / eV	*f*	Charge Transferred	Distance charge transferred / Å	Single Electron transition
T_1	2.70		0.63	2.66	H -> L 30%, H-1 -> L+1 27%, H-1 -> L 26%, H -> L+1 4%, H-9 -> L 3%, H-8 -> L 2%, H-8 -> L+1 2%
T_2	2.73		0.65	2.68	H-1 -> L 44%, H -> L 16%, H -> L+1 15%, H-1 -> L+1 12%, H-9 -> L 2%
S_1	2.76	0.17	0.68	2.93	H-1 -> L 69%, H -> L+1 20%, H-3 -> L 2%
S_2	2.88	0.26	0.74	3.32	H -> L 73%, H-1 -> L+1 15%, H-9 -> L 2%

f is oscillator strength

Table S3.10 Excited state energies and the corresponding charge transfer metrics for **BN3** as obtained at the SCS-CC2/cc-pVDZ level of theory.

Excited State	Energy / eV	*f*	Charge Transferred	Distance charge transferred / Å	Single Electron transition
T_1	2.51		0.57	2.36	H -> L 79%, H-4 -> L 5%, H-2 -> L+1 5%, H-1 -> L 4%, H-3 -> L+1 2%
T_2	2.65		0.57	2.36	H -> L+1 55%, H-2 -> L 17%, H-4 -> L+1 7%, H-3 -> L 6%, H-1 -> L+1 5%, H-8 -> L 2%
S_1	2.54	0.08	0.74	3.12	H -> L 84%, H-4 -> L 3%, H-3 -> L+1 3%, H-2 -> L+1

					2%, H-13 -> L 2%
S_2	2.95	0.15	0.68	3.21	H -> L+1 61%, H-3 -> L 15%, H-2 -> L 13%

f is oscillator strength

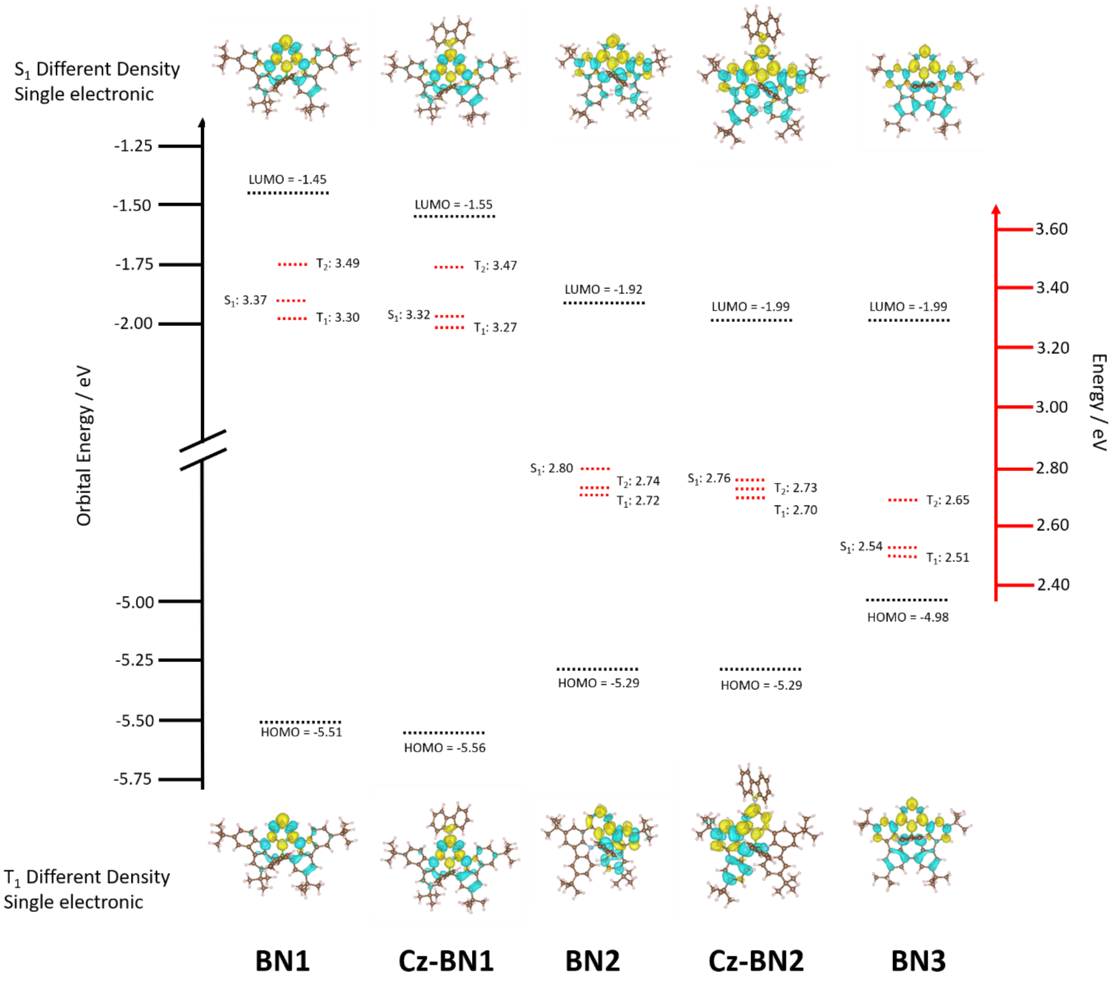

Figure S3.2 HOMO and LUMO energies (doted black lines) as computed at the PBE0/6-31G(d,p) level of theory of the investigated emitters. Excited states energies (red doted lines) and corresponding different density plots for each emitter calculated at the SCS-CC2/cc-pVDZ level of theory including single excitation only contributions

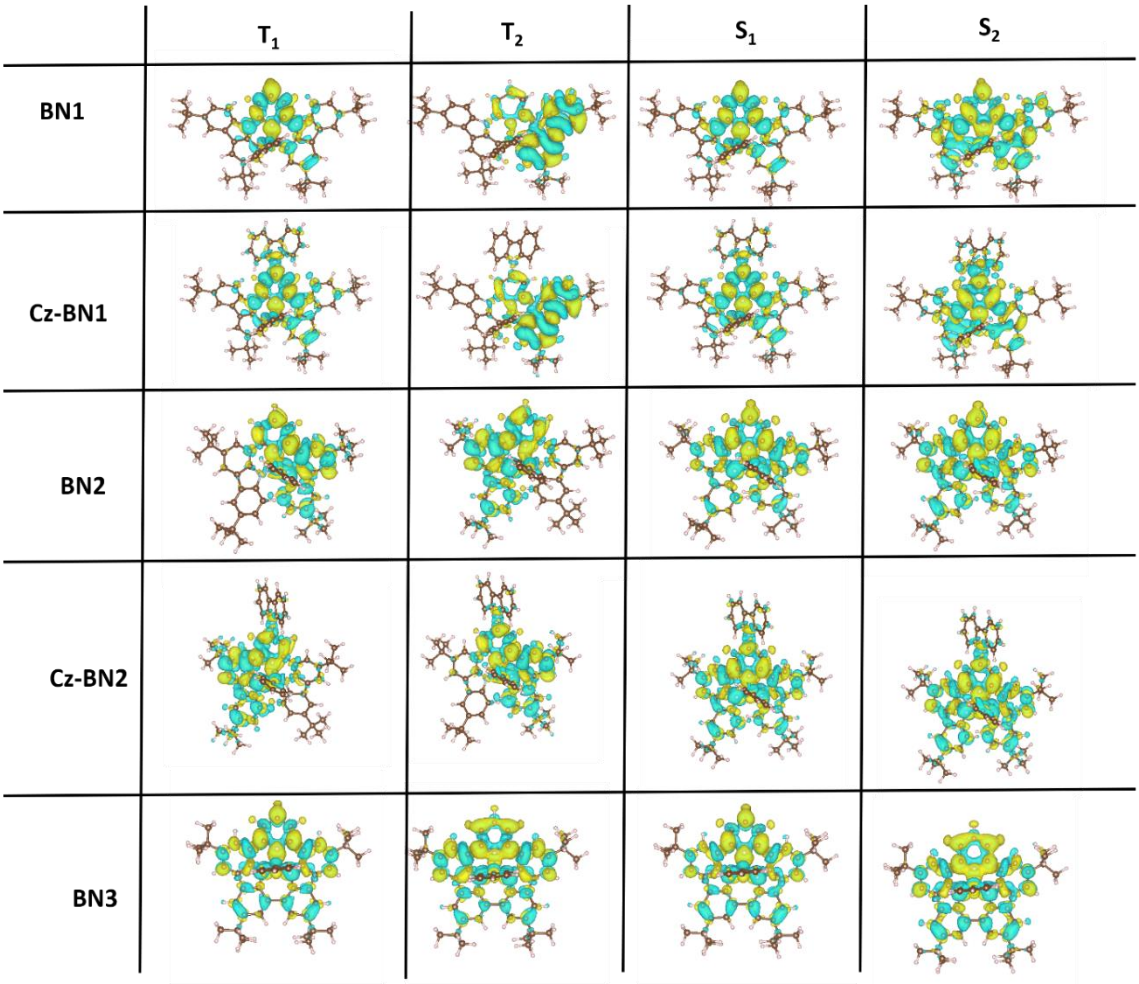

Figure S3.3 T_1, T_2, S_1 and S_2 difference density plots for each emitter computed at the SCS-CC2/cc-pVDZ level including both first and second order contributions.

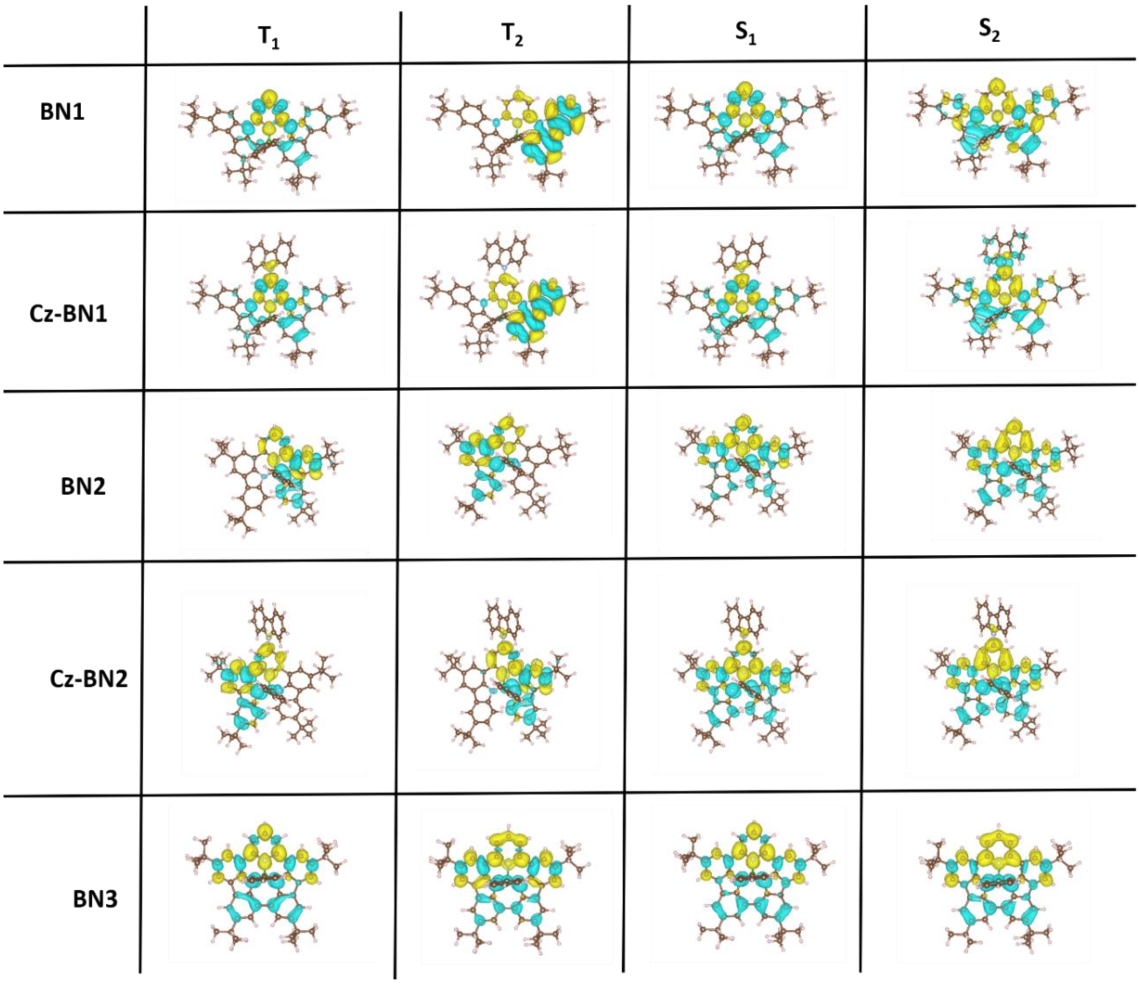

Figure S3.4 T_1, T_2, S_1 and S_2 difference density plots for each emitter computed at the SCS-CC2/cc-pVDZ level including first contributions only.

Table S3.11 First 20 singlet excited states of **BN1** calculated at TDA-M062X/ 6-31G(d,p) level.

Excited state	Energy / eV	f^{a}	ϕ_S^{b}
S_1	3.694	0.533	0.637
S_2	4.291	0.067	0.646
S_3	4.518	0.054	0.707
S_4	4.544	0.056	0.826
S_5	4.589	0.015	0.690
S_6	4.651	0.036	0.769
S_7	4.707	0.017	0.787
S_8	4.808	0.054	0.708
S_9	4.824	0.038	0.534
S_{10}	4.995	0.285	0.851
S_{11}	5.010	0.163	0.855
S_{12}	5.145	0.031	0.422
S_{13}	5.202	0.027	0.578

S_{14}	5.247	0.063	0.621
S_{15}	5.379	0.009	0.669
S_{16}	5.409	0.041	0.800
S_{17}	5.481	0.198	0.661
S_{18}	5.507	0.021	0.757
S_{19}	5.581	0.046	0.738
S_{20}	5.616	0.171	0.903

[a]Oscillator strength, [b]Calculated from the overlap of attachment and detachment density

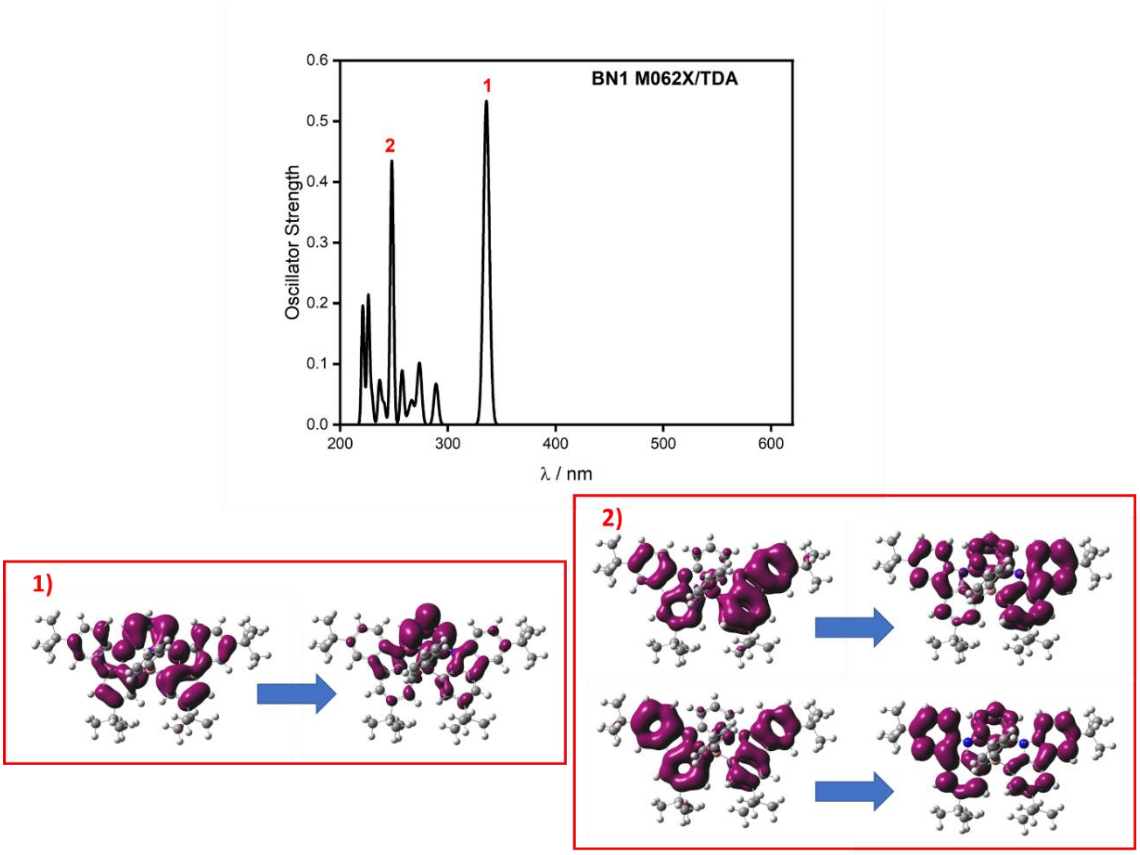

Figure S3.5 Simulated absorption spectra for **BN1** from first 20 singlet excited states calculated at TDA-M062X/ 6-31G(d,p) level and attachment/detachment density plots for transitions 1 and 2 with high oscillator strengths.

Table S3.12 First 20 singlet excited states of **CzBN1** calculated at TDA-M062X/ 6-31G(d,p) level.

Excited state	Energy / eV	f^a	ϕ_S^b
S_1	3.673	0.437	0.634
S_2	4.130	0.462	0.605
S_3	4.373	0.061	0.635
S_4	4.482	0.079	0.767
S_5	4.505	0.094	0.723
S_6	4.552	0.024	0.592

S_7	4.618	0.085	0.831
S_8	4.660	0.007	0.864
S_9	4.707	0.009	0.801
S_{10}	4.776	0.042	0.729
S_{11}	4.805	0.030	0.575
S_{12}	4.909	0.001	0.459
S_{13}	4.991	0.230	0.849
S_{14}	5.007	0.143	0.837
S_{15}	5.093	0.043	0.463
S_{16}	5.116	0.015	0.627
S_{17}	5.134	0.036	0.777
S_{18}	5.142	0.075	0.785
S_{19}	5.153	0.086	0.765
S_{20}	5.187	0.072	0.604

[a]Oscillator strength, [b]Calculated from the overlap of attachment and detachment density

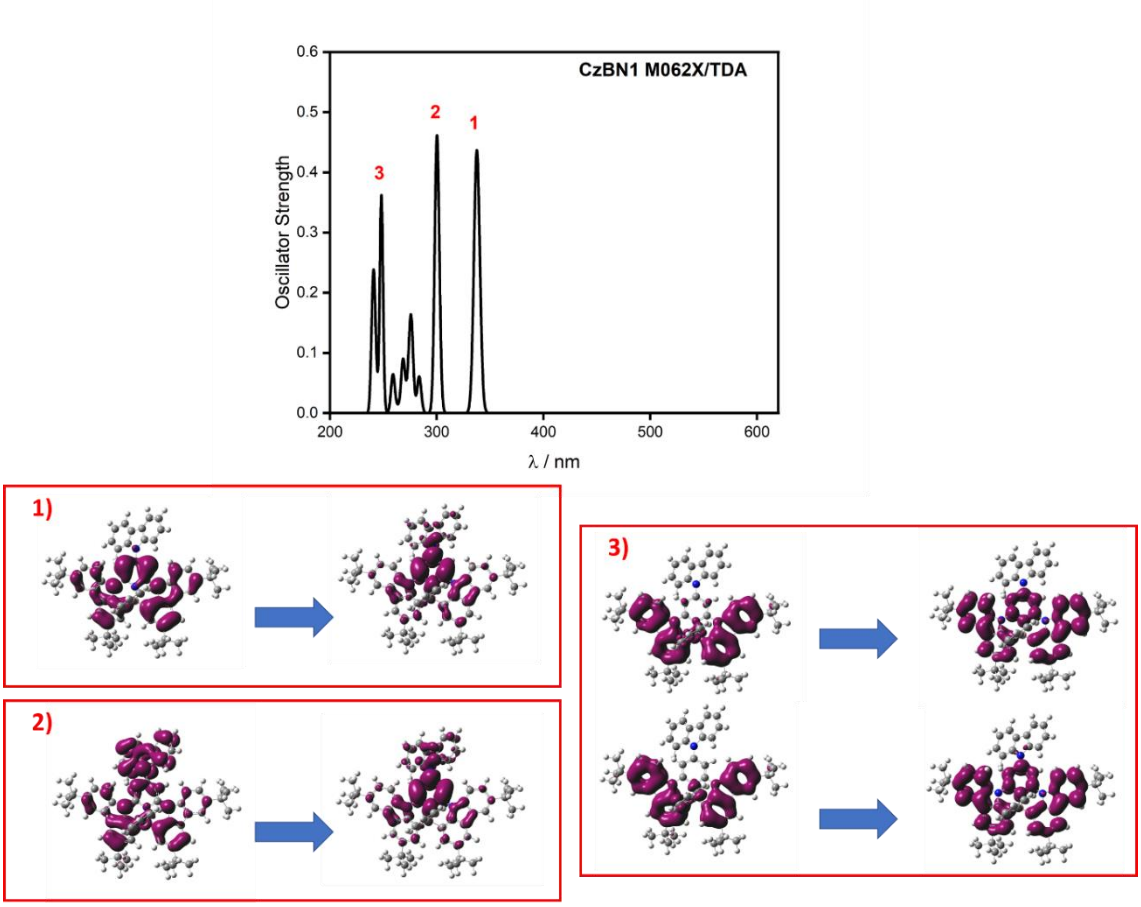

Figure S3.6 Simulated absorption spectra for **CzBN1** from first 20 singlet excited states calculated at TDA-M062X/ 6-31G(d,p) level and attachment/detachment density plots for transitions 1, 2 and 3 with high oscillator strengths.

Table S3.13 First 20 singlet excited states of **BN2** calculated at TDA-M062X/ 6-31G(d,p) level.

Excited state	Energy / eV	f^a	ϕ_S^b
S_1	3.119	0.254	0.621
S_2	3.322	0.156	0.540
S_3	3.681	0.004	0.620
S_4	3.786	0.102	0.667
S_5	4.044	0.032	0.574
S_6	4.063	0.087	0.590
S_7	4.420	0.037	0.772
S_8	4.530	0.042	0.567
S_9	4.570	0.041	0.578
S_{10}	4.610	0.008	0.652
S_{11}	4.711	0.168	0.819
S_{12}	4.811	0.174	0.679
S_{13}	4.863	0.159	0.627
S_{14}	5.008	0.008	0.408
S_{15}	5.088	0.007	0.777
S_{16}	5.126	0.027	0.780
S_{17}	5.203	0.019	0.678
S_{18}	5.267	0.161	0.864
S_{19}	5.299	0.020	0.853
S_{20}	5.326	0.096	0.831

[a]Oscillator strength, [b]Calculated from the overlap of attachment and detachment density

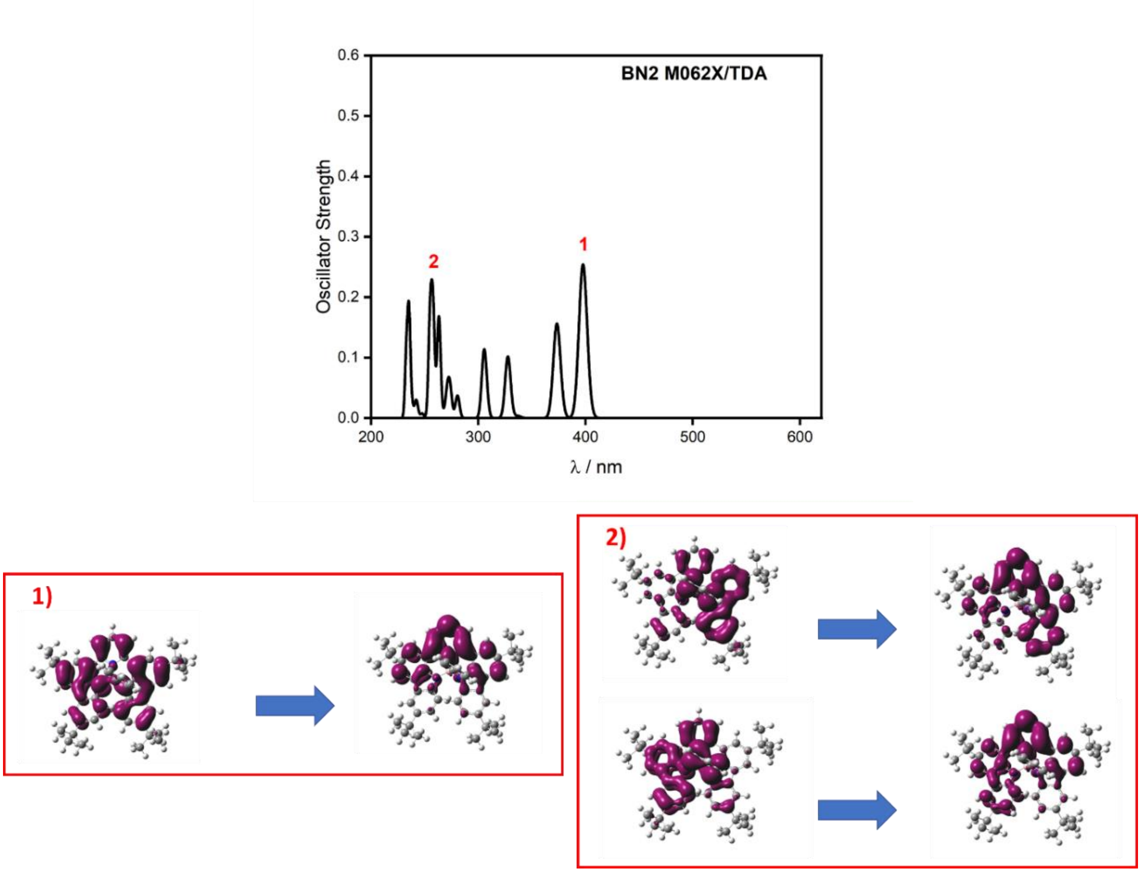

Figure S3.7 Simulated absorption spectra for **BN2** from first 20 singlet excited states calculated at TDA-M062X/ 6-31G(d,p) level and attachment/detachment density plots for transitions 1 and 2 with high oscillator strengths.

Table S3.14 First 20 singlet excited states of **CzBN2** calculated at TDA-M062X/ 6-31G(d,p) level.

Excited state	Energy / eV	*f*[a]	ϕ_S[b]
S_1	3.123	0.224	0.611
S_2	3.272	0.301	0.539
S_3	3.611	0.006	0.600
S_4	3.723	0.148	0.636
S_5	3.983	0.013	0.567
S_6	4.001	0.113	0.617
S_7	4.080	0.391	0.587
S_8	4.340	0.021	0.735
S_9	4.421	0.046	0.675
S_{10}	4.458	0.017	0.541
S_{11}	4.574	0.015	0.480
S_{12}	4.608	0.010	0.774
S_{13}	4.640	0.000	0.368
S_{14}	4.654	0.153	0.858
S_{15}	4.693	0.074	0.840

S_{16}	4.795	0.086	0.516
S_{17}	4.862	0.212	0.724
S_{18}	4.922	0.028	0.471
S_{19}	5.036	0.027	0.755
S_{20}	5.073	0.014	0.700

[a]Oscillator strength, [b]Calculated from the overlap of attachment and detachment density

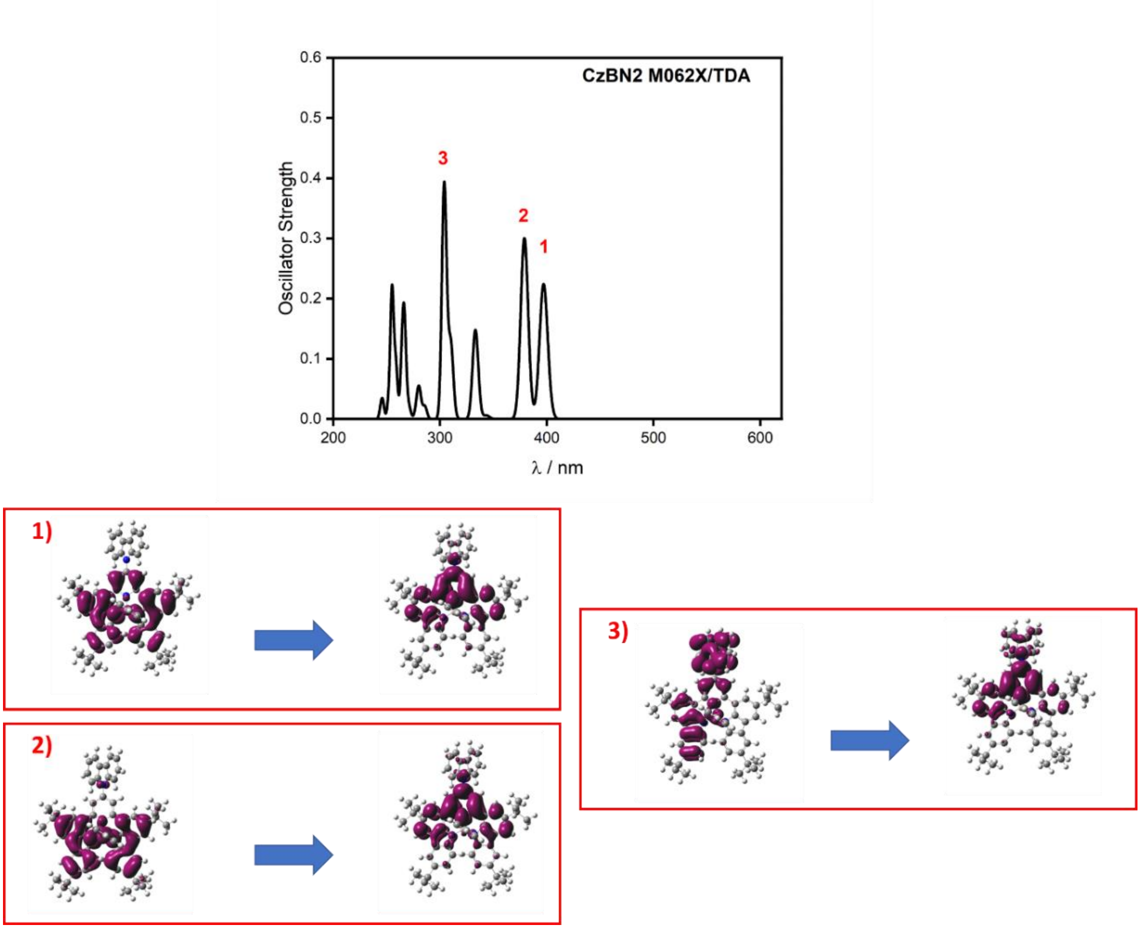

Figure S3.8 Simulated absorption spectra for **CzBN2** from first 20 singlet excited states calculated at TDA-M062X/ 6-31G(d,p) level and attachment/detachment density plots for transitions 1 and 2 with high oscillator strengths.

Table S3.15 First 20 singlet excited states of **BN3** calculated at TDA-M062X/ 6-31G(d,p) level.

Excited state	Energy / eV	f^a	$\phi_S{}^b$
S_1	2.790	0.083	0.525
S_2	3.332	0.211	0.600
S_3	3.568	0.001	0.531
S_4	3.778	0.044	0.756
S_5	3.872	0.017	0.734

S_6	3.983	0.221	0.587
S_7	4.030	0.081	0.599
S_8	4.327	0.350	0.805
S_9	4.415	0.057	0.650
S_{10}	4.471	0.168	0.765
S_{11}	4.523	0.087	0.578
S_{12}	4.570	0.106	0.622
S_{13}	4.709	0.013	0.384
S_{14}	4.785	0.043	0.893
S_{15}	4.853	0.115	0.830
S_{16}	4.945	0.001	0.430
S_{17}	5.060	0.002	0.727
S_{18}	5.159	0.026	0.588
S_{19}	5.190	0.036	0.821
S_{20}	5.236	0.051	0.828

[a]Oscillator strength, [b]Calculated from the overlap of attachment and detachment density

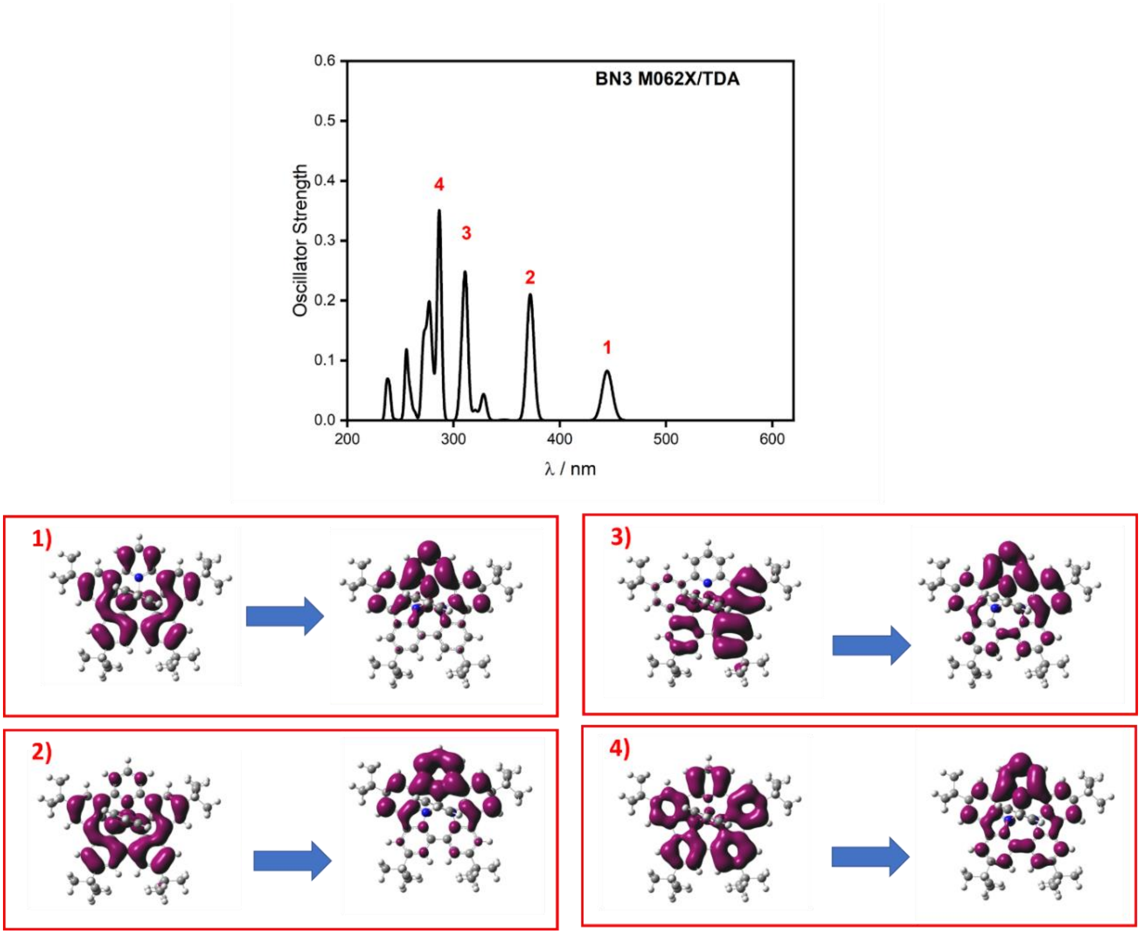

Figure S3.9 Simulated absorption spectra for **BN3** from first 20 singlet excited states calculated at TDA-M062X/ 6-31G(d,p) level and attachment/detachment density plots for transitions 1, 2, 3 and 4 with high oscillator strengths.

S5 Photophysical Properties

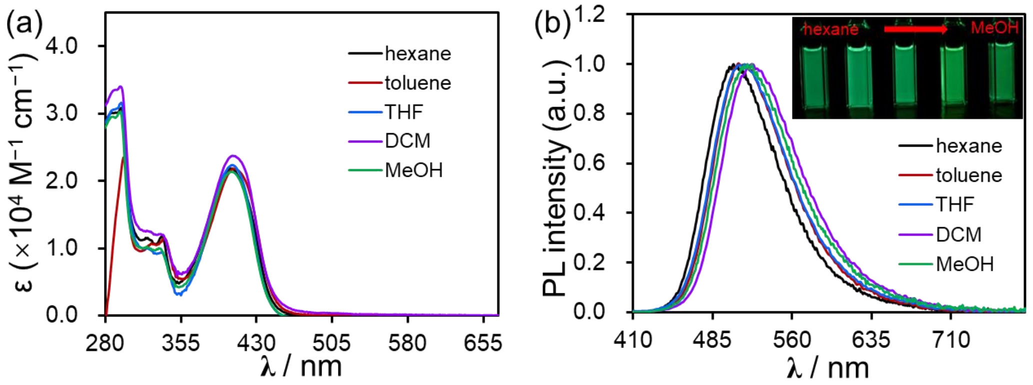

Figure S4.1 a) Absorption and b) emission spectra of **BN1** in various solvents (0.01 M) at 298 K. Photographs showing the solution color under irradiated with 365 nm UV light.

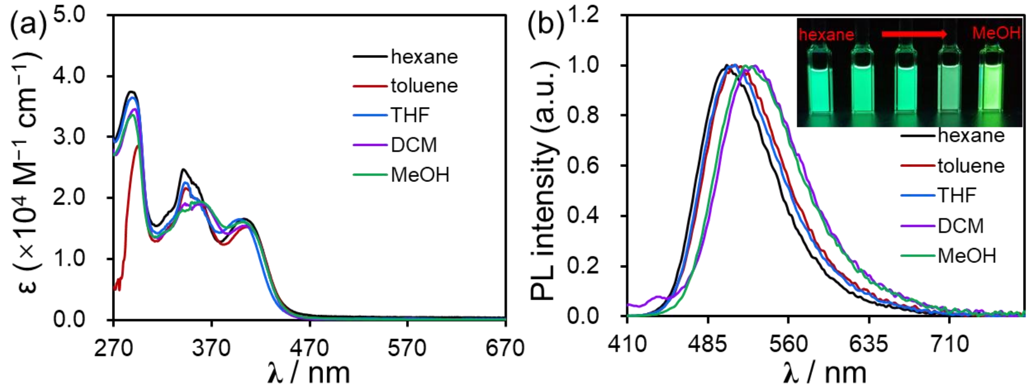

Figure S4.2 a) Absorption and b) emission spectra of **TCz-BN1** in various solvents (0.01 M) at 298 K. Photographs showing the solution color under 365 nm UV light.

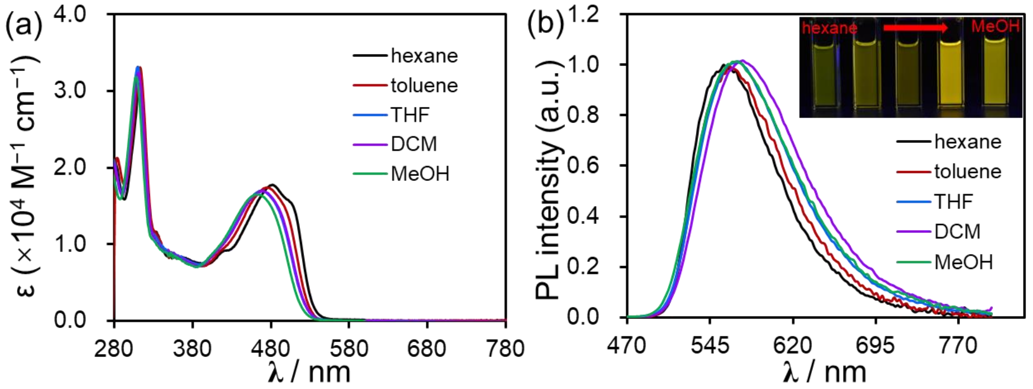

Figure S4.3 a) Absorption and b) emission spectra of **BN2** in various solvents (0.01 M) at 298 K. Photographs showing the solution color under 365 nm UV light.

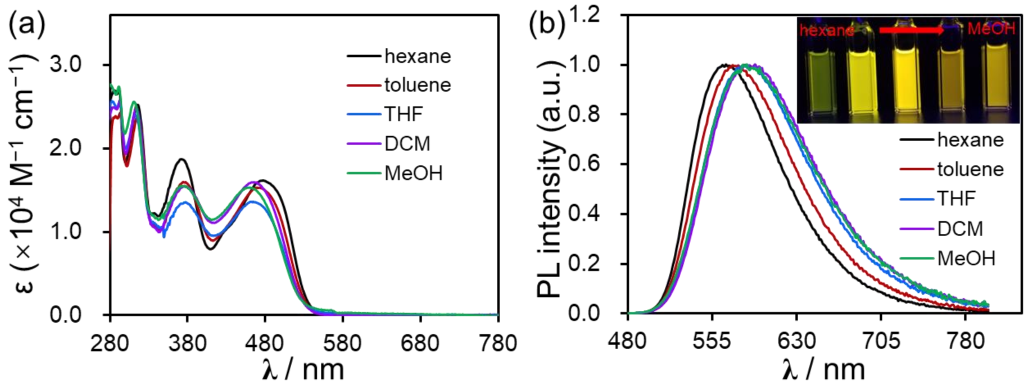

Figure S4.4 a) Absorption and b) emission spectra of **TCz-BN2** in various solvents (0.01 M) at 298 K. Photographs showing the solution color under 365 nm UV light.

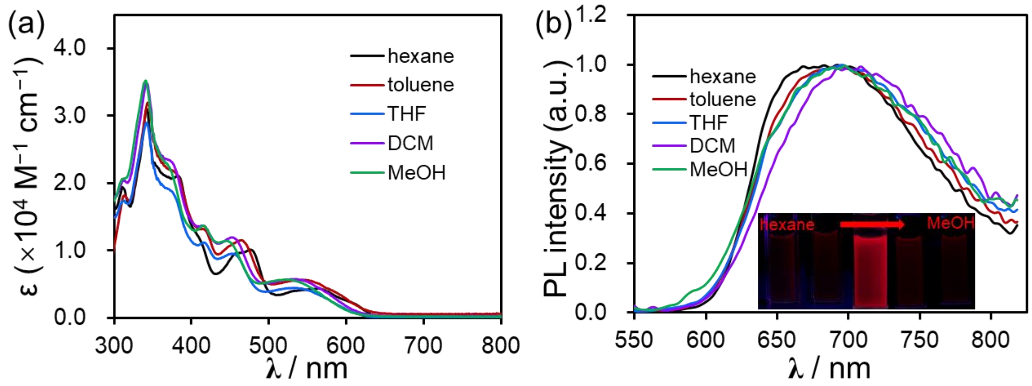

Figure S4.5 a) Absorption and b) emission spectra of **BN3** in various solvents (0.01 M) at 298 K. Photographs showing the solution color under 365 nm UV light.

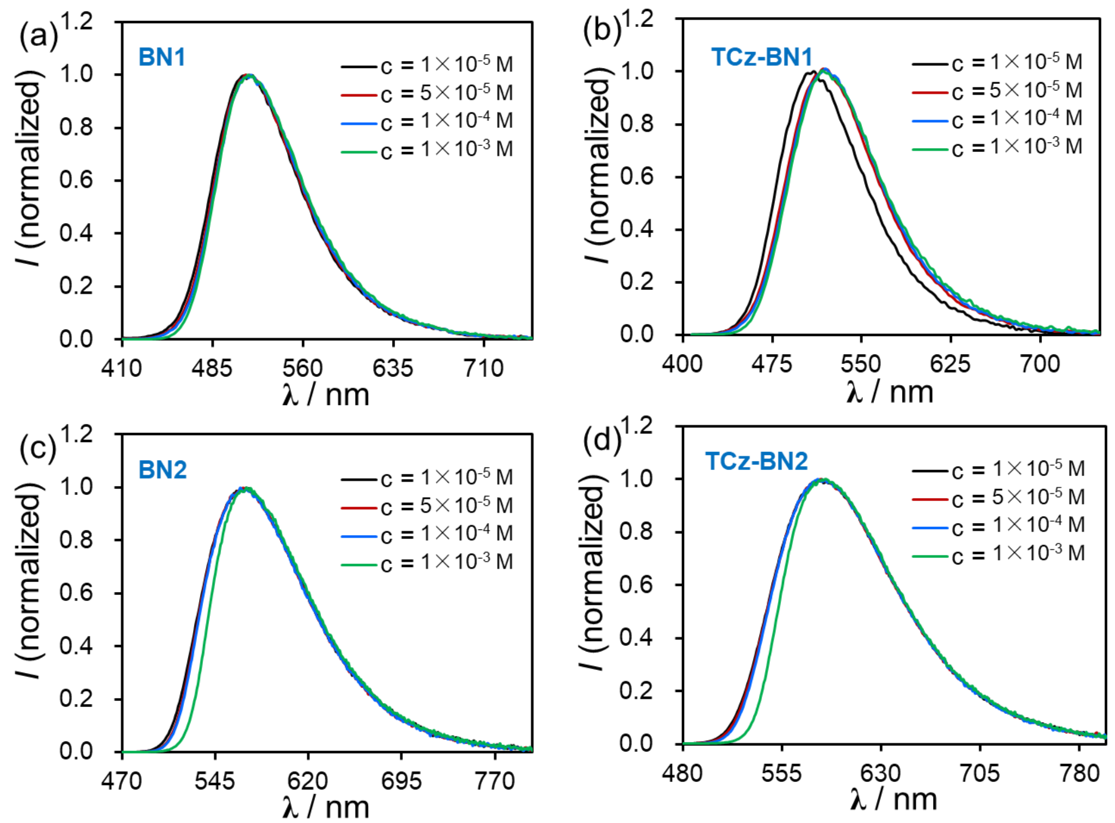

Figure S4.6 Concentration-dependent emission spectra of a) **BN1**, b) **TCz-BN1**, c) **BN2** and d) **TCz-BN2** in THF at 298K.

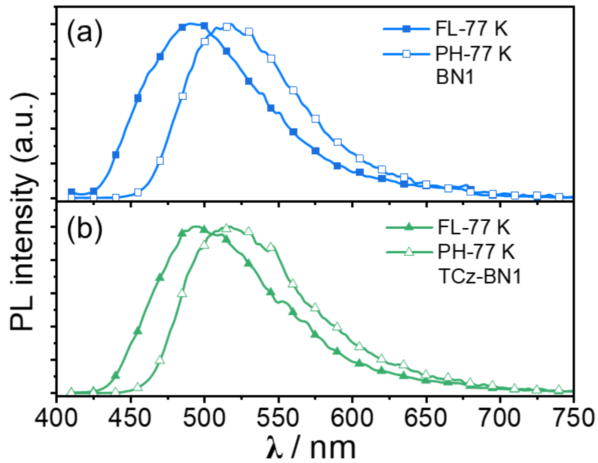

Figure S4.7 The fluorescence and phosphorescence spectra of a) **BN1** and b) **TCz-BN1** in 5 wt% doped PMMA films.

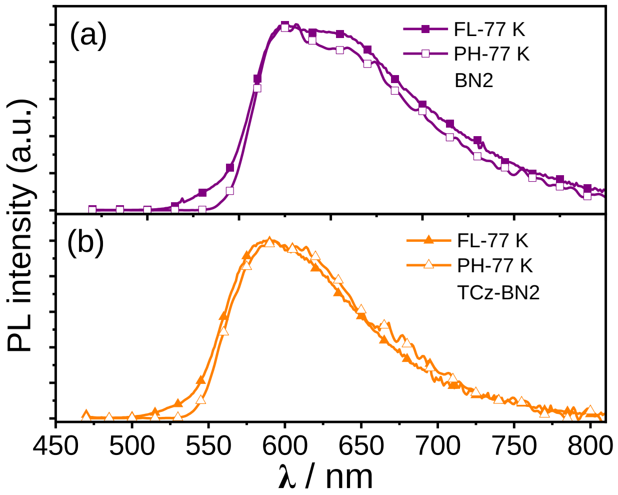

Figure S4.8 The fluorescence and phosphorescence spectra of a) **BN2** and b) **TCz-BN2** in 5 wt% doped PMMA films.

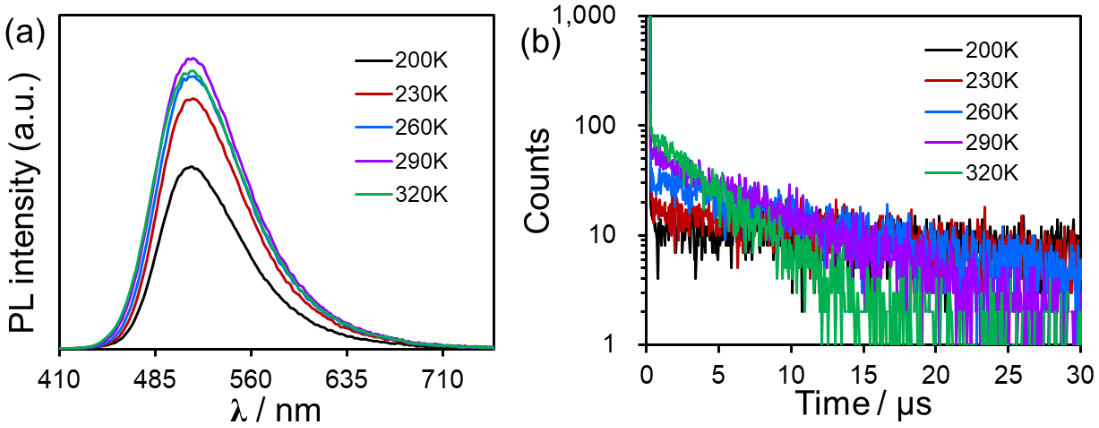

Figure S4.9 Temperature-dependent a) fluorescence and b) transient decay spectra of **BN1** in 2-Me-THF (0.01 mM) recorded between 200 K and 320 K under N_2.

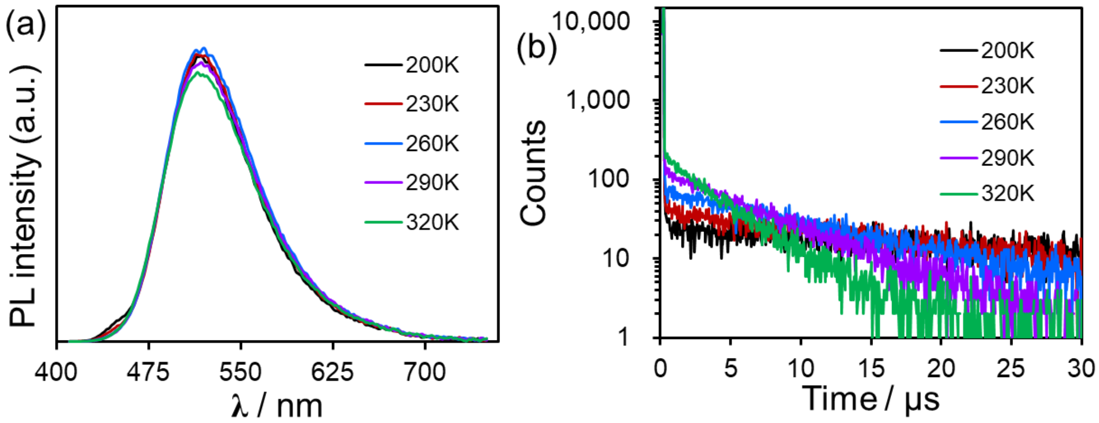

Figure S4.10 Temperature-dependent a) fluorescence and b) transient decay spectra of **TCz-BN1** in 2-Me-THF (0.01 mM) recorded between 200 K and 320 K under N_2.

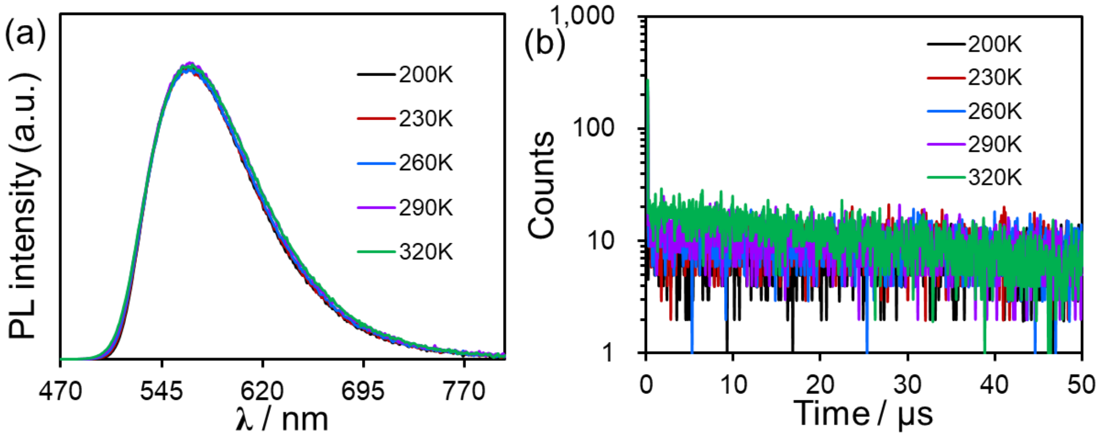

Figure S4.11 Temperature-dependent a) fluorescence and b) transient decay spectra of **BN2** in 2-Me-THF (0.01 mM) recorded between 200 K and 320 K under N_2.

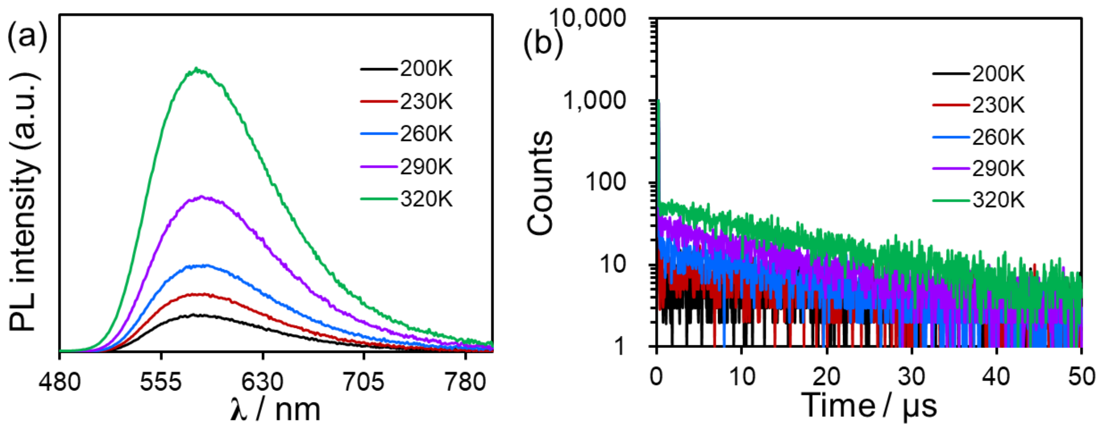

Figure S4.12 Temperature-dependent a) fluorescence and b) transient decay spectra of **TCz-BN2** in 2-Me-THF (0.01 mM) recorded between 200 K and 320 K under N_2.

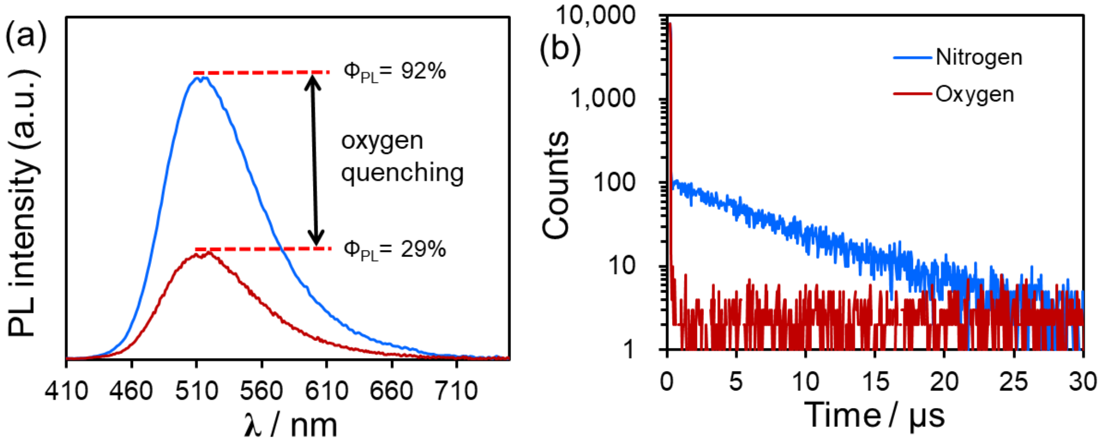

Figure S4.13 Change in a) PL spectra and b) transient PL decay curves of **BN1** in THF at 298 K under different atmospheres: N_2 (blue line) and O_2 bubbling for 2 min (red line).

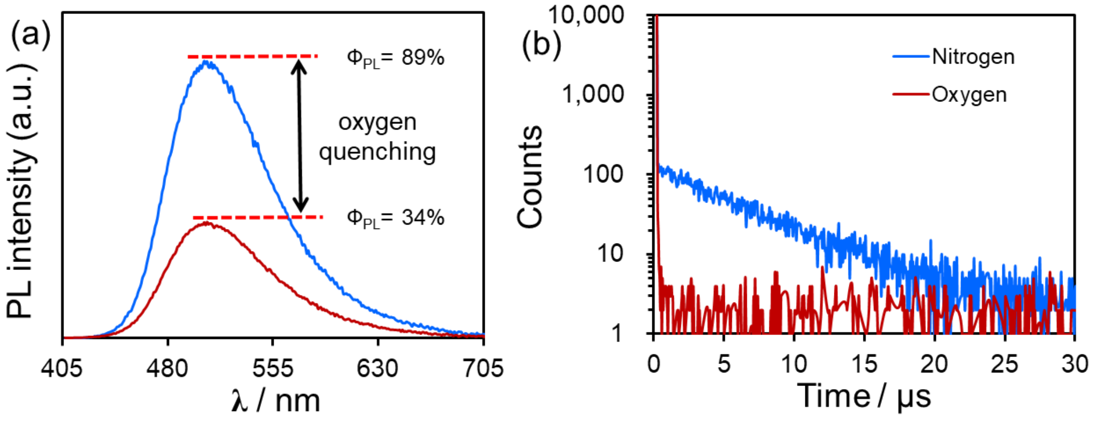

Figure S4.14 Change in a) PL spectra and b) transient PL decay curves of **TCz-BN1** in THF at 298 K under different atmospheres: N_2 (blue line) and O_2 bubbling for 2 min (red line).

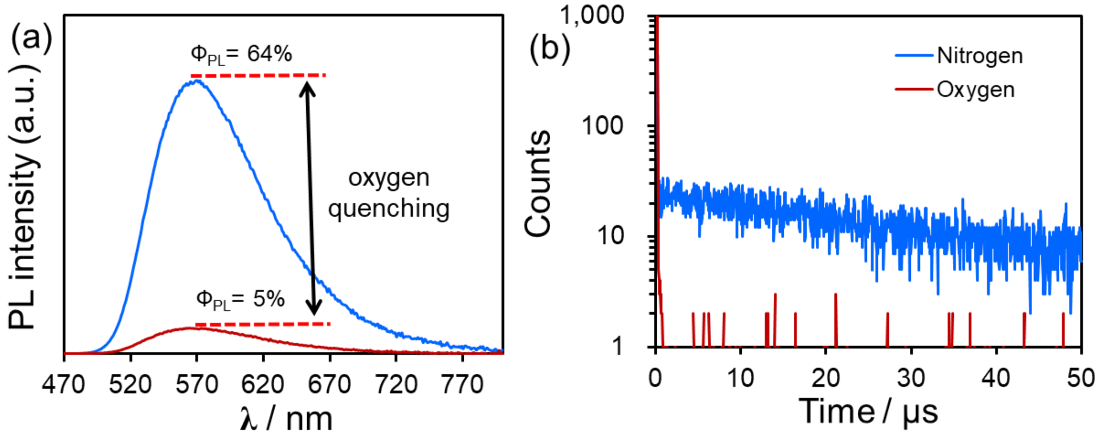

Figure S4.15 Change in a) PL spectra and b) transient PL decay curves of **BN2** in THF at 298 K under different atmospheres: N_2 (blue line) and O_2 bubbling for 2 min (red line).

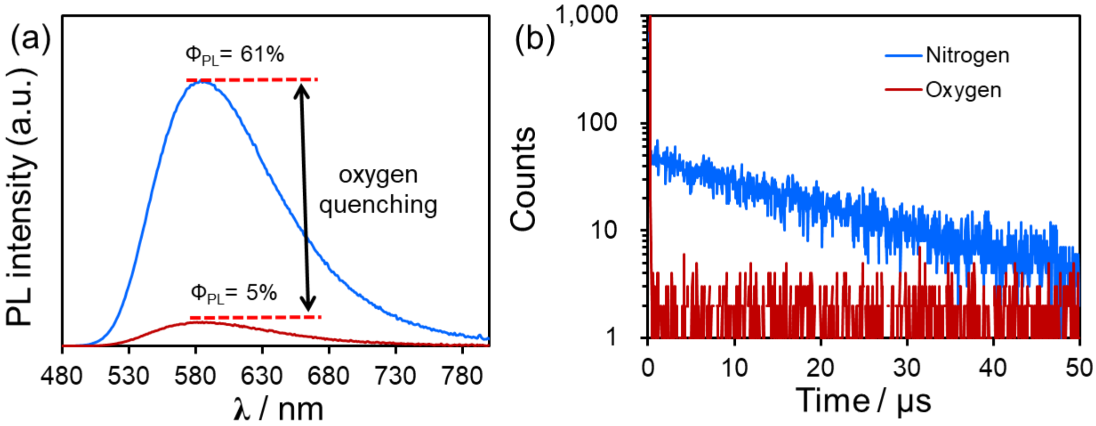

Figure S4.16 Change in a) PL spectra and b) transient PL decay curves of **TCz-BN2** in THF at 298 K under different atmospheres: N_2 (blue line) and O_2 bubbling for 2 min (red line).

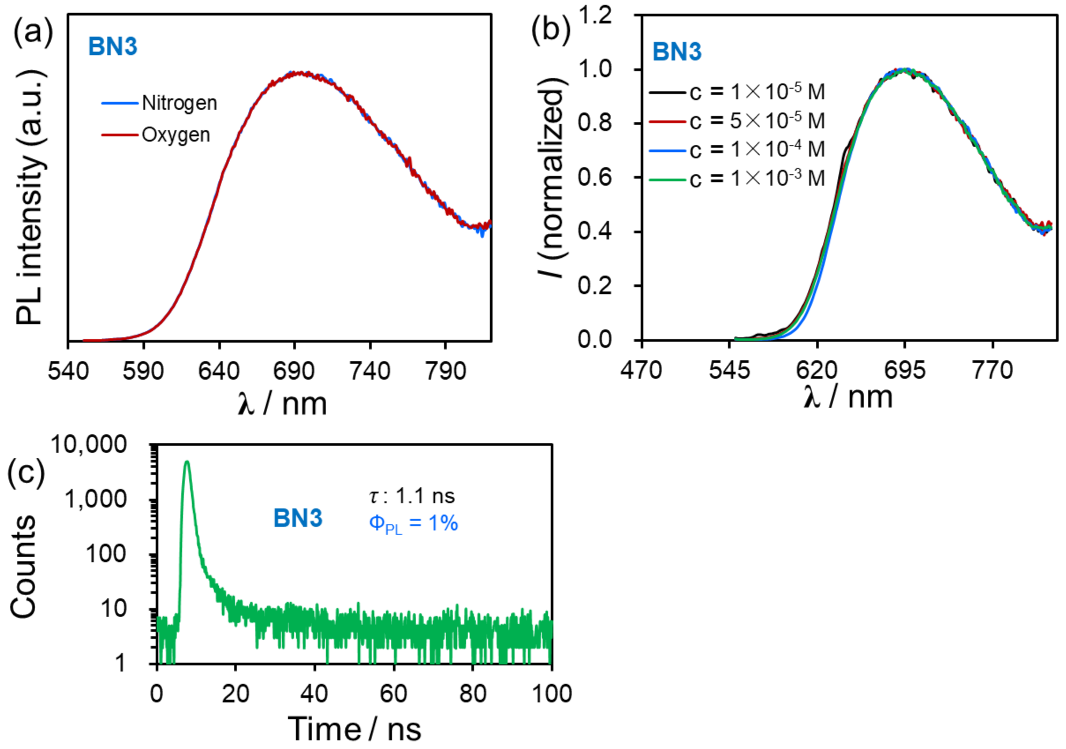

Figure S4.17 a) Change in PL spectra of **BN3** in THF at 298 K under different atmospheres: N_2 (blue line) and O_2 bubbling for 2 min (red line); b) Concentration-dependent emission spectra of **BN3** in THF at 298 K; c) Transient PL decay curves of **BN3** in THF at 298 K under N_2.

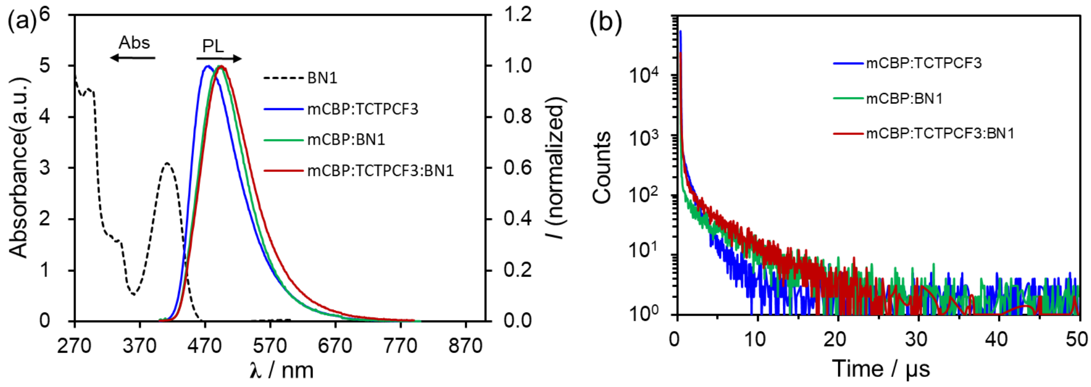

Figure S4.18 a) The absorption spectrum of **BN1** (neat film, dashed lines) and emission spectra of mixed films of mCBP: 20wt% TCTPCF3, mCBP: 2wt% **BN1** and mCBP: 20 wt% TCTPCF3: 2wt% **BN1**; b) PL decay cures of the mixed films of mCBP: 20wt% TCTPCF3, mCBP: 2wt% **BN1** and mCBP: 20 wt% TCTPCF3: 2wt% **BN1**.

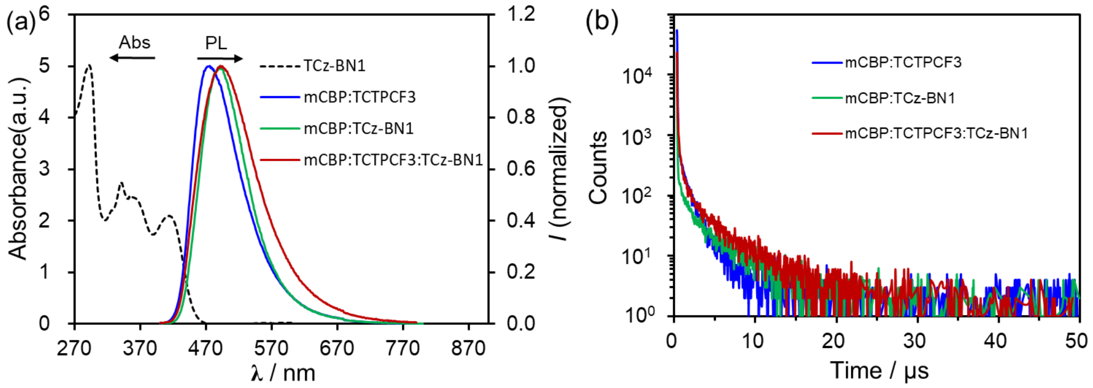

Figure S4.19 a) The absorption spectrum of **TCz-BN1** (neat film, dashed lines) and emission spectra of mixed films of mCBP: 20wt% TCTPCF3, mCBP: 2wt% **TCz-BN1** and mCBP: 20 wt% TCTPCF3: 2wt% **TCz-BN1**; b) PL decay cures of the mixed films of mCBP: 20wt% TCTPCF3, mCBP: 2wt% **TCz-BN1** and mCBP: 20 wt% TCTPCF3: 2wt% **TCz-BN1**.

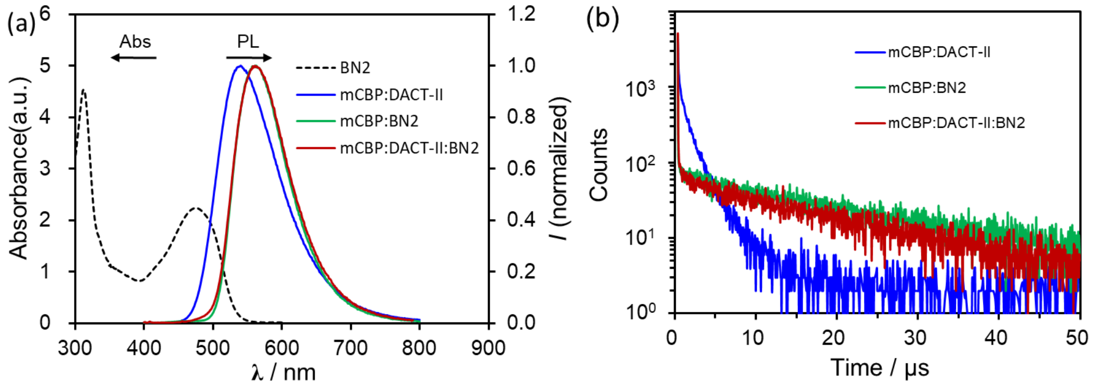

Figure S4.20 a) The absorption spectrum of **BN2** (neat film, dashed lines) and emission spectra of the mixed films of mCBP: 20wt% DACT-II, mCBP: 5wt% **BN2** and mCBP: 20wt% DACT-II: 5wt% **BN2**; b) PL decay cures of the mixed films of mCBP: 20wt% DACT-II, mCBP: 5wt% **BN2** and mCBP: 20wt% DACT-II: 5wt% **BN2**.

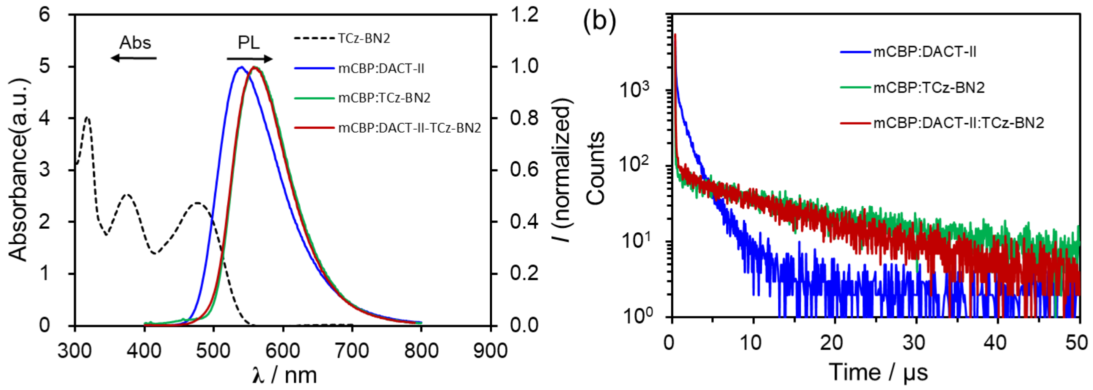

Figure S4.21 a) The absorption spectrum of **TCz-BN2** (neat film, dashed lines) and emission spectra of the mixed films of mCBP: 20wt% DACT-II, mCBP: 5wt% **TCz-BN2** and mCBP: 20wt% DACT-II: 5wt% **TCz-BN2**; b) PL decay cures of the mixed

films of mCBP: 20wt% DACT-II, mCBP: 5wt% **TCz-BN2** and mCBP: 20wt% DACT-II: 5wt% **TCz-BN2**.

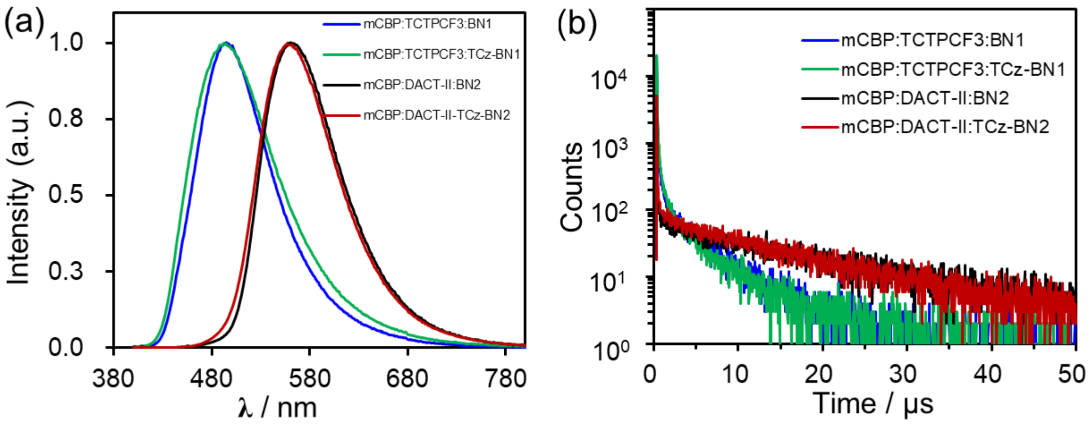

Figure S4.22 (a) The PL spectra and (b) transient PL decay curves of mCBP: 20 wt% sensitizer: 2 wt%/5 wt% emitters.

Table S4.1 The basic material parameters and chemical structures of TCTPCF3 and DACT-II.

Name	Structure	λ_{em} [nm]	HOMO [eV]	LUMO [eV]
TCTPCF3[19]	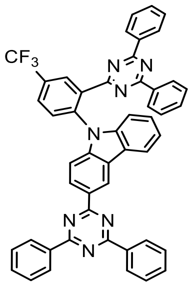	468	5.86	2.98
DACT-II[20]	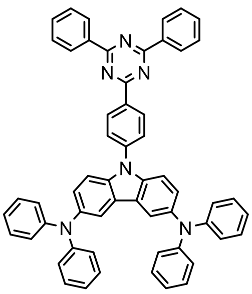	516	5.5	3.2

Table S4.2 Photophysical properties of **BN1**, **TCz-BN1**, **BN2** and **TCz-BN2**-doped film at 300 K.

Compounds	λ_{em} a) [nm]	Φ b) [%]	τ_{PF} c) [ns]	τ_{DF} c) [μs]

mCBP: 20wt% TCTPCF3	474	83	28.0	1.2
mCBP: 2wt% BN1	492	75	45.9	4.5
mCBP: 20wt% TCTPCF3: 2wt% BN1	495	74	46.3	2.8
mCBP: 2wt% TCz-BN1	491	71	46.9	3.0
mCBP: 20wt% TCTPCF3: 2wt% TCz-BN1	492	67	56.9	2.2
mCBP: 20wt% DACT-II	520	91	94.0	1.5
mCBP: 5wt% BN2	559	53	85.3	20.4
mCBP: 20wt% DACT-II: 5wt% BN2	557	48	105.2	14.4
mCBP: 5wt% TCz-BN2	560	62	98.2	15.1
mCBP: 20wt% DACT-II: 5wt% TCz-N2	558	67	114.2	12.2

[a] PL emission maximum; [b] Absolute PL quantum yields evaluated using an integrating sphere; [c] PL lifetimes of prompt fluorescence (τ_{PF}) and delayed fluorescence (τ_{DF}).

S6 Organic Light Emitting Diode (OLED) Device Data

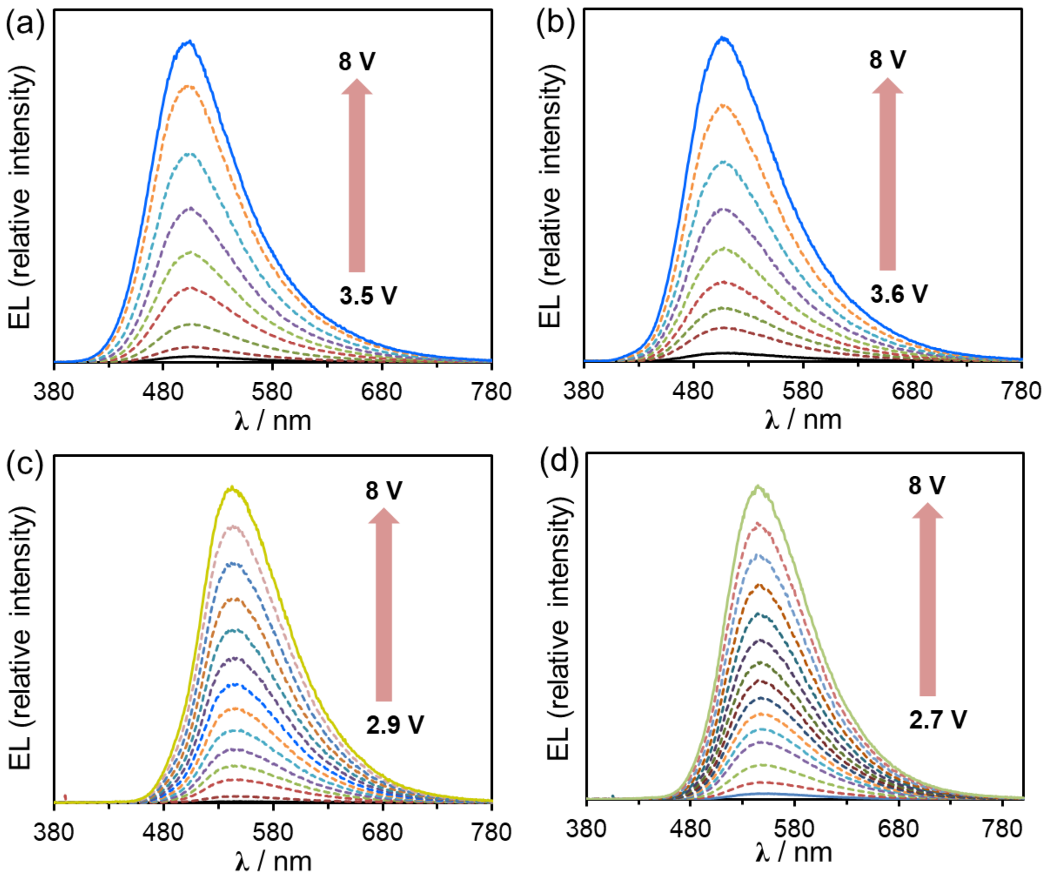

Figure S5.1 The EL spectra of devices based on (a) **BN1**, (b) **TCz-BN1**, (c) **BN2** and (d) **TCz-BN2** under different voltage.

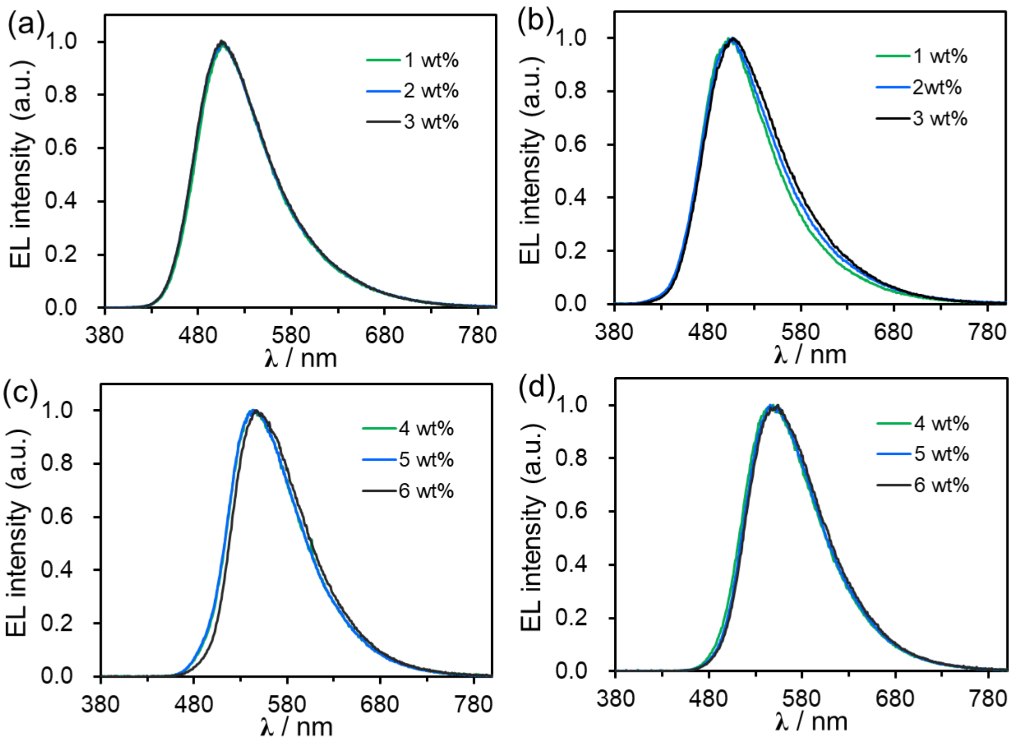

Figure S5.2 EL spectra of devices based on (a) **BN1**, (b) **TCz-BN1**, (c) **BN2** and (d) **TCz-BN2** with increased concentrations from 1 wt% to 6 wt%.

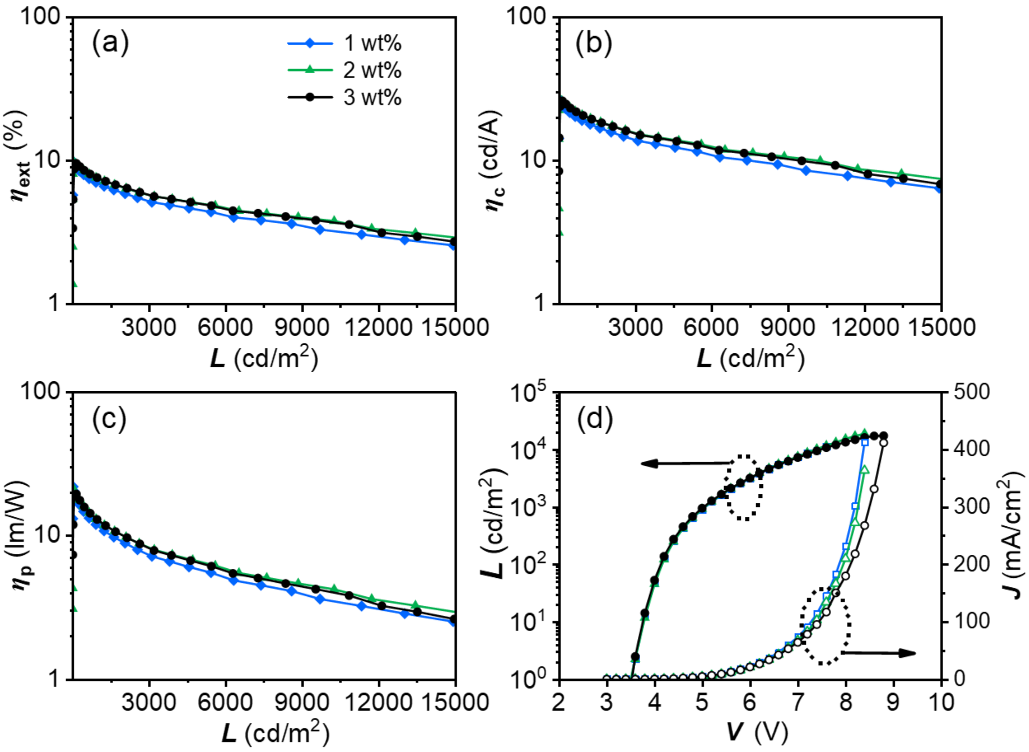

Figure S5.3 EL characteristics of OLED devices based on **BN1** increased concentrations from 1 wt% to 3 wt%. (a) The external quantum efficiency (η_{ext}), (b) current efficiency (η_c) and (c) power efficiency (η_p) versus luminance (L) curves for devices; (d) Luminance (L)–voltage (V)–current density (J) characteristics for the devices.

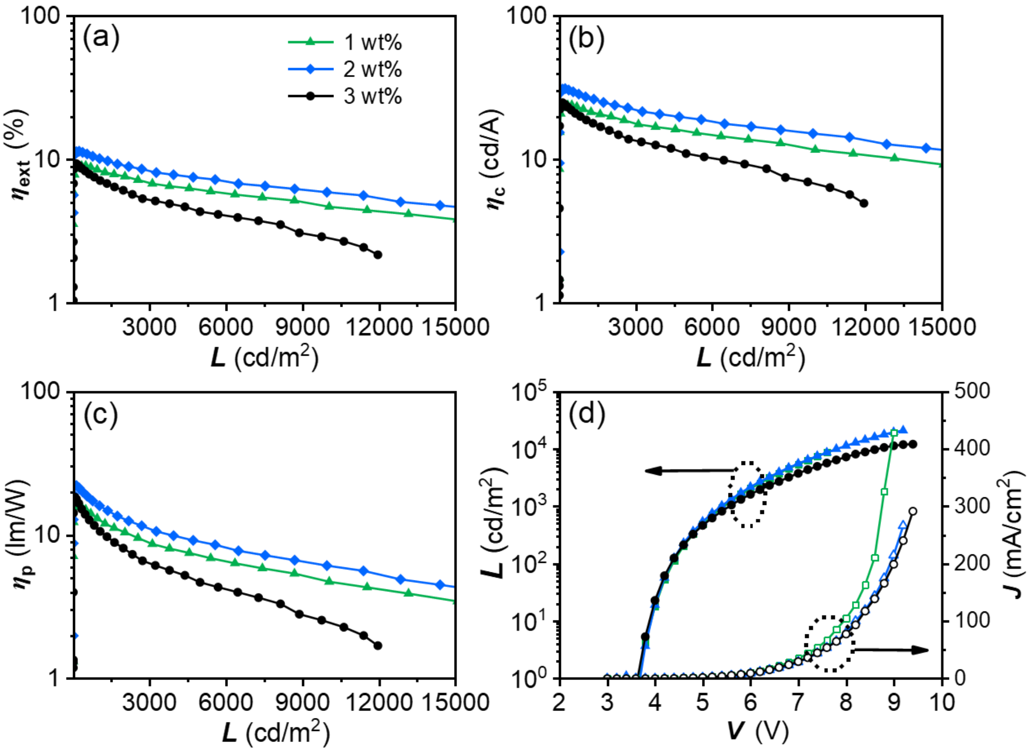

Figure S5.4 EL characteristics of OLED devices based on **TCz-BN1** increased concentrations from 1 wt% to 3 wt%. (a) The external quantum efficiency (η_{ext}), (b) current efficiency (η_c) and (c) power efficiency (η_p) versus luminance (L) curves for devices; (d) Luminance (L)–voltage (V)–current density (J) characteristics for the devices.

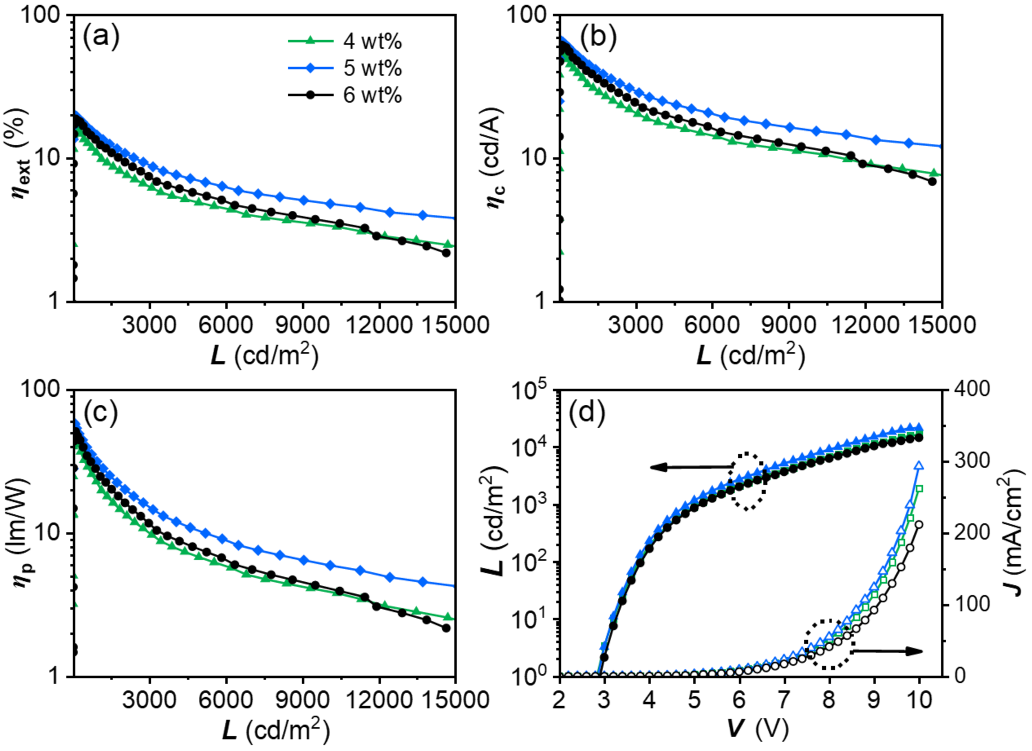

Figure S5.5 EL characteristics of OLED devices based on **BN2** increased

concentrations from 4 wt% to 6 wt%. (a) The external quantum efficiency (η_{ext}), (b) current efficiency (η_c) and (c) power efficiency (η_p) versus luminance (L) curves for devices; (d) Luminance (L)–voltage (V)–current density (J) characteristics for the devices.

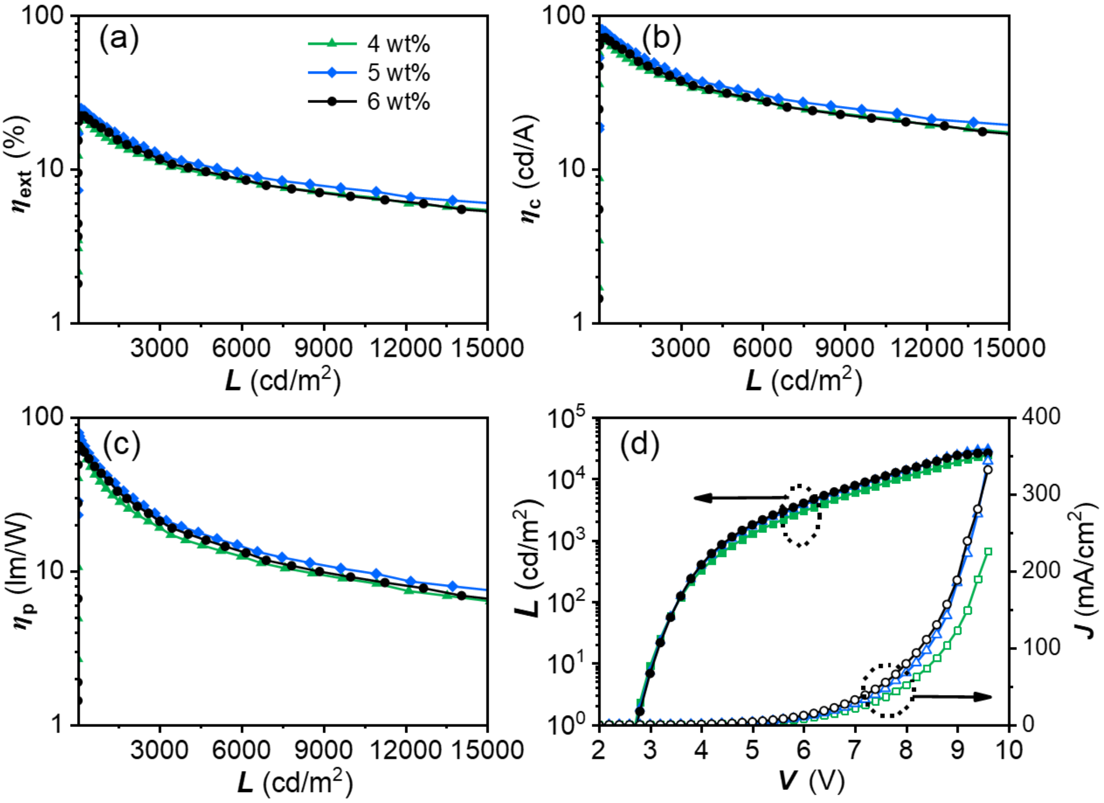

Figure S5.6 EL characteristics of OLED devices based on **TCz-BN2** increased concentrations from 4 wt% to 6 wt%. (a) The external quantum efficiency (η_{ext}), (b) current efficiency (η_c) and (c) power efficiency (η_p) versus luminance (L) curves for devices; (d) Luminance (L)–voltage (V)–current density (J) characteristics for the devices.

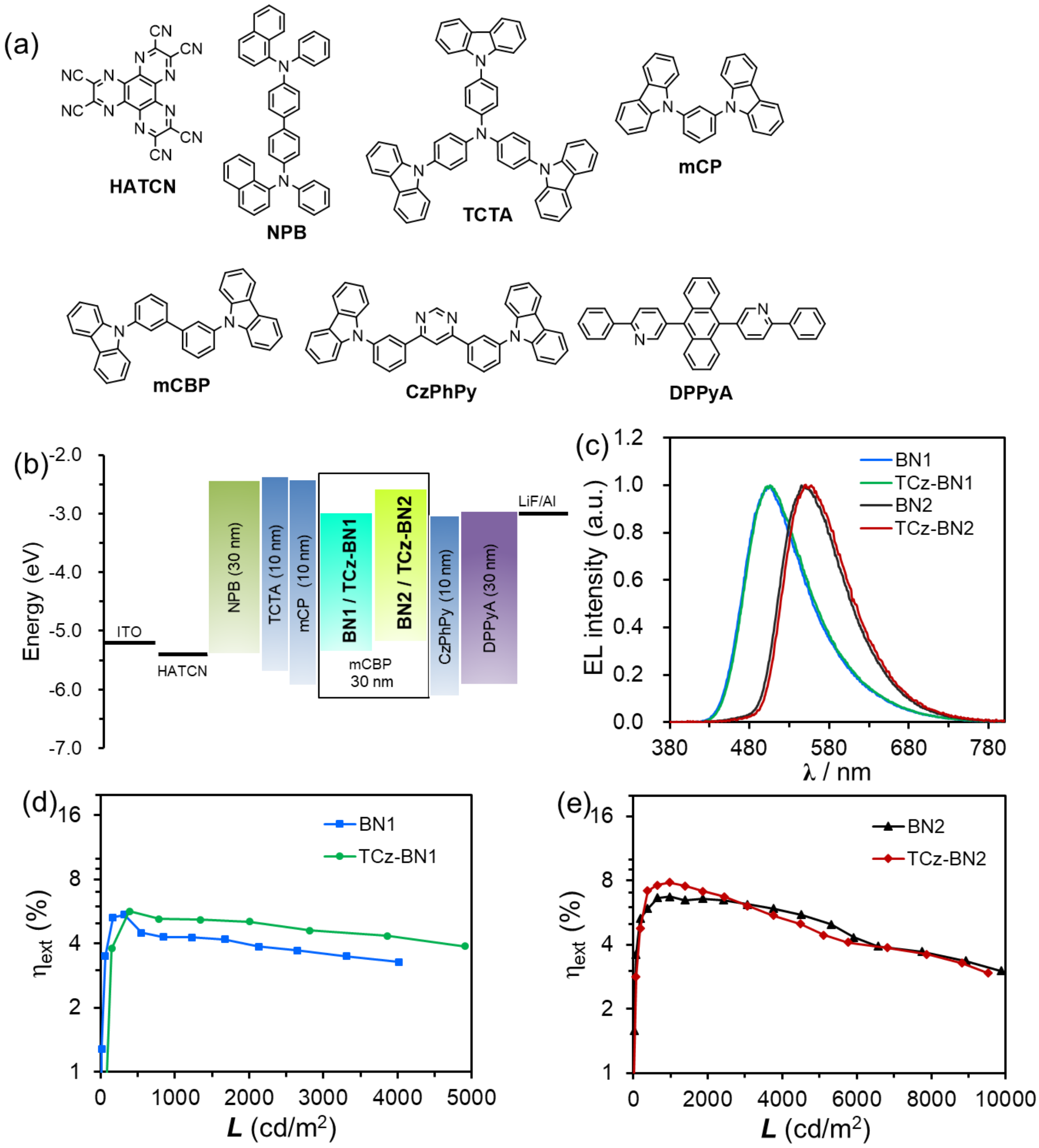

Figure S5.7 (a) Molecule structures of the functional materials used in OLED devices. (b) The device structures of the OLEDs. (c) The EL spectra the devices without sensitizer and (d)/(e) external quantum efficiency (η_{ext})-brightness characteristics of the devices with 2 wt% **BN1**, **TCz-BN1** or 5 wt% **BN2**, **TCz-BN2** as the emitters in device structure of ITO/ NPB (30 nm)/ TCTA (10 nm)/ mCP (10 nm)/ mCBP: emitters (30 nm)/ CzPhPy (10 nm)/ DPPyA (30 nm)/ LiF (0.5 nm)/ Al (150 nm).

Table S5.1 Summary of the device performances of the OLEDs.

Concentration	λ_{EL} [a)] [nm]	V_{on} [b)] [V]	L_{max} [c)] [cd m^{-2}]	η_c [d)] [cd A^{-1}]	η_p [e)] [lm W^{-1}]	η_{ext} [f)] [%]	CIE (x,y) [a)]
**BN1** (1 wt%)	506	3.5	19044	26.7/23.5/19.6	19.5/16.1/11.9	9.7/8.5/7.2	(0.27, 0.49)
**BN1** (2 wt%)	507	3.5	18180	26.6/21.6/19.1	22.0/14.8/11.9	9.9/8.0/7.0	(0.27, 0.49)
**BN1** (3 wt%)	507	3.5	17496	26.1/21.9/19.5	19.6/14.4/11.8	9.5/8.1/7.2	(0.27, 0.49)
**TCz-BN1** (1 wt%)	505	3.7	18684	24.0/23.9/21.5	16.4/15.1/12.0	9.1/9.0/8.2	(0.25, 0.47)
**TCz-BN1** (2 wt%)	507	3.6	20952	31.3/29.8/27.5	22.2/18.7/16.0	11.5/11.0/10.2	(0.28,0.48)
**TCz-BN1** (3 wt%)	507	3.6	12096	25.0/22.2/18.9	18.4/14.0/10.6	9.4/8.4/7.2	(0.28, 0.48)

a) Value taken at a luminance around 1000 cd m^{-2}; b) V_{on}: turn-on voltage at the luminance of 1 cd m^{-2}; c) L = Luminescence; d) Current efficiency (η_c): maximum, then values at 500 and 1000 cd m^{-2}; e) Power efficiency (η_p): maximum, then values at 500 and 1000 cd m^{-2}; f) External quantum efficiency (η_{ext}): maximum, then values at 500 and 1000 cd m^{-2}

Table S5.2 Summary of the device performances of the OLEDs.

Concentration	λ_{EL} [a)] [nm]	V_{on} [b)] [V]	L_{max} [c)] [cd m^{-2}]	η_c [d)] [cd A^{-1}]	η_p [e)] [lm W^{-1}]	η_{ext} [f)] [%]	CIE (x,y) [a)]
**BN2** (4 wt%)	545	2.9	13900	56.2/42.5/32.8	51.4/29.0/19.8	17.2/16.5/12.8	(0.40, 0.57)
**BN2** (5 wt%)	547	2.9	21576	66.1/55.7/44.9	59.0/39.7/28.2	19.9/16.7/13.5	(0.40, 0.57)
**BN2** (6 wt%)	547	2.9	12200	61.9/50.8/41.1	51.2/34.7/24.8	18.9/15.4/12.4	(0.42, 0.56)
**TCz-BN2** (4 wt%)	548	2.7	24660	74.4/63.7/52.6	68.6/47.7/34.4	22.8/19.5/16.1	(0.41, 0.56)
**TCz-BN2** (5 wt%)	554	2.7	30708	81.8/70.1/61.2	79.7/52.4/41.8	25.1/21.4/18.7	(0.41,0.56)
**TCz-BN2** (6 wt%)	554	2.7	26388	72.1/64.4/56.6	65.1/48.1/38.7	22.3/19.9/17.5	(0.42, 0.55)

a) Value taken at a luminance around 1000 cd m^{-2}; b) V_{on}: turn-on voltage at the luminance of 1 cd m^{-2}; c) L = Luminescence; d) Current efficiency (η_c): maximum, then values at 500 and 1000 cd m^{-2}; e) Power efficiency (η_p): maximum, then values at 500 and 1000 cd m^{-2}; f) External quantum efficiency (η_{ext}): maximum, then values at 500 and 1000 cd m^{-2}.

Table S5.3 Summary of the device performances of the OLEDs without sensitizers.

Compounds	λ_{EL} [a] [nm]	V_{on} [b] [V]	L_{max} [c] [cd m^{-2}]	η_c [d] [cd A^{-1}]	η_p [e] [lm W^{-1}]	η_{ext} [f] [%]
**BN1**	505	3.8	4013	14.8	8.6	5.5
**TCz-BN1**	505	3.8	4906	15.3	8.9	5.7
**BN2**	545	3.8	9870	17.1	10.2	6.7
**TCz-BN2**	556	3.8	9530	19.9	11.7	7.8

[a] Value taken at a luminance around 1000 cd m^{-2}; [b] V_{on}: turn-on voltage at the luminance of 1 cd m^{-2}; [c] L = Luminescence; [d] Maximum current efficiency (η_c), [e] Power efficiency (η_p), [f] External quantum efficiency (η_{ext}).

Table S5.4 Summary OLED devices of tetracoordinate boron TADF compounds reported in the literatures.

Emitter	λ_{PL} (nm)	PLQY (%)	T_d (°C)	λ_{EL} (nm)	EQE (%)	Refs.
PrFPCz	485 (in Tol)	22.5 (in Tol)	313	522	7.6	21
PrFCzP	495 (in Tol)	23.4 (in Tol)	265	505	4.8	
PrFTPA	560 (in Tol)	40.8 (in Tol)	286	520	13.5	
1	504 (in Film)	97 (in Film)	-	494	22.7	22
fppyBTPA	494 (in Film)	72 (in Film)	392	494	20.2	23
dfppyBTPA	508 (in Film)	100 (in Film)	359	508	26.6	
3	508 (in film)	42 (in Film)	-	512	5.6	24
5	541 (in film)	29 (in Film)	-	548	8.3	
BFPD	468 (in Tol)	3.1 (in Tol)	280	518	10.5	25
1	468 (in Tol)	39 (in Tol)	401	422	8.8	26
2	522 (in Tol)	61 (in Tol)	451	423	18.0	
3	537 (in Tol)	65 (in Tol)	393	444	17.5	
Dye	721 (in film)	70 (in Film)	-	721	10	27

S7 ^{1}H NMR, ^{13}C NMR and ^{11}B NMR Data

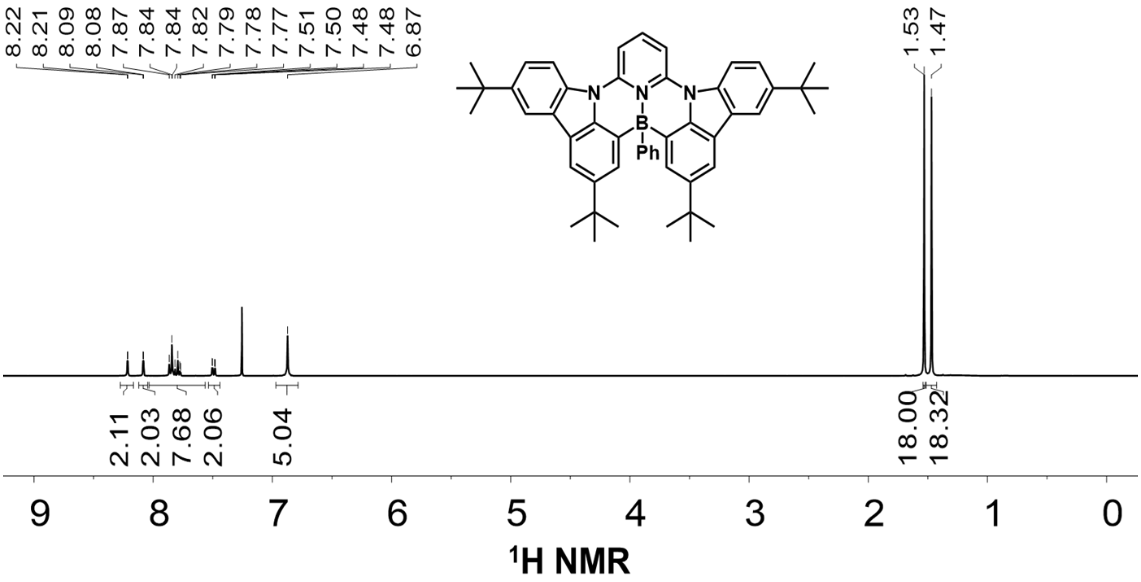

Figure S6.1 ^{1}H NMR spectrum of **BN1** (400 MHz, $CDCl_3$)

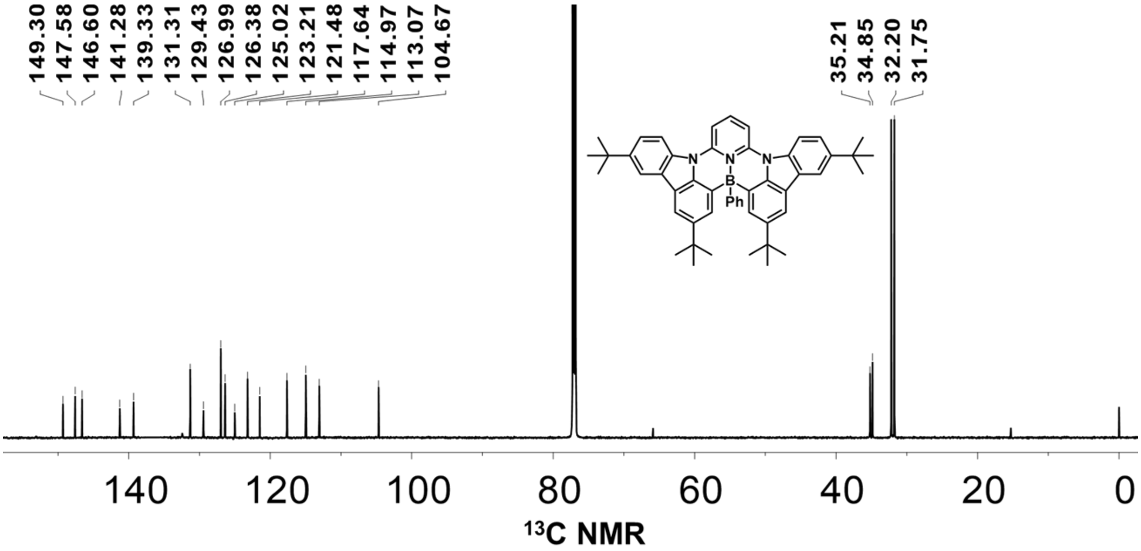

Figure S6.2 ^{13}C NMR spectrum of **BN1** (176 MHz, $CDCl_3$)

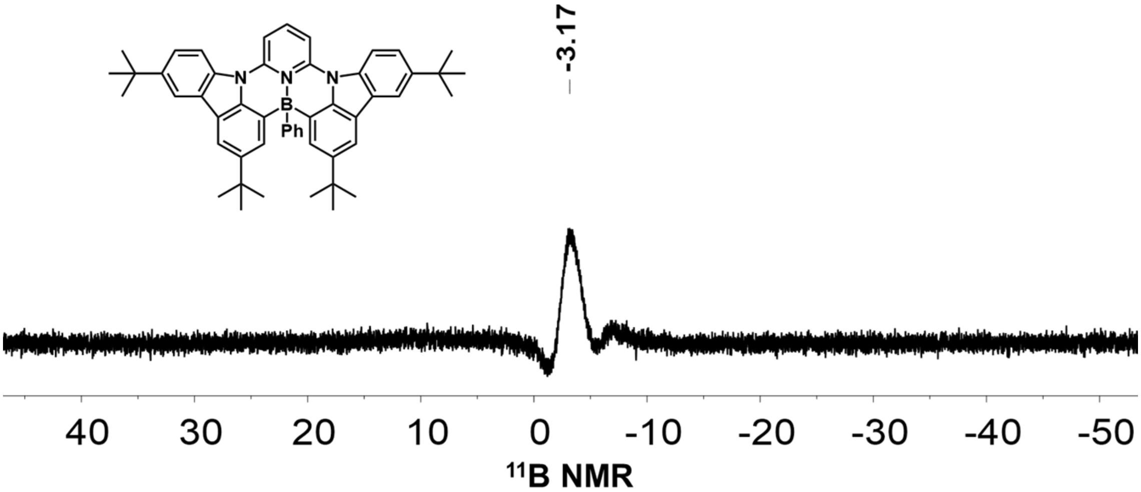

Figure S6.3 ^{11}B NMR spectrum of **BN1** (225 MHz, $CDCl_3$)

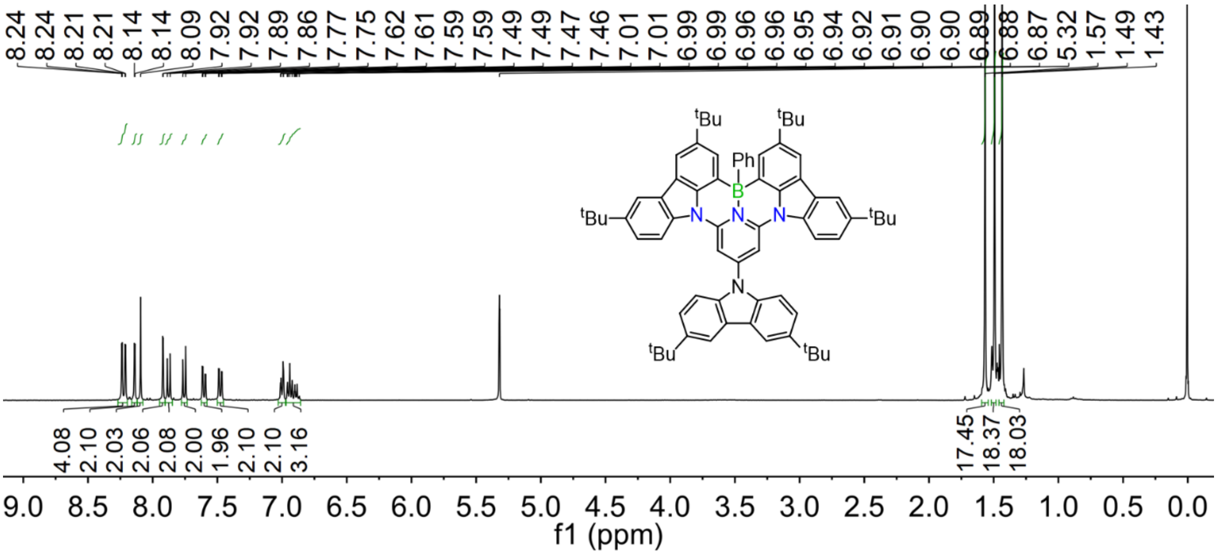

Figure S6.4 ^{1}H NMR spectrum of **TCz-BN1** (400 MHz, CD_2Cl_2)

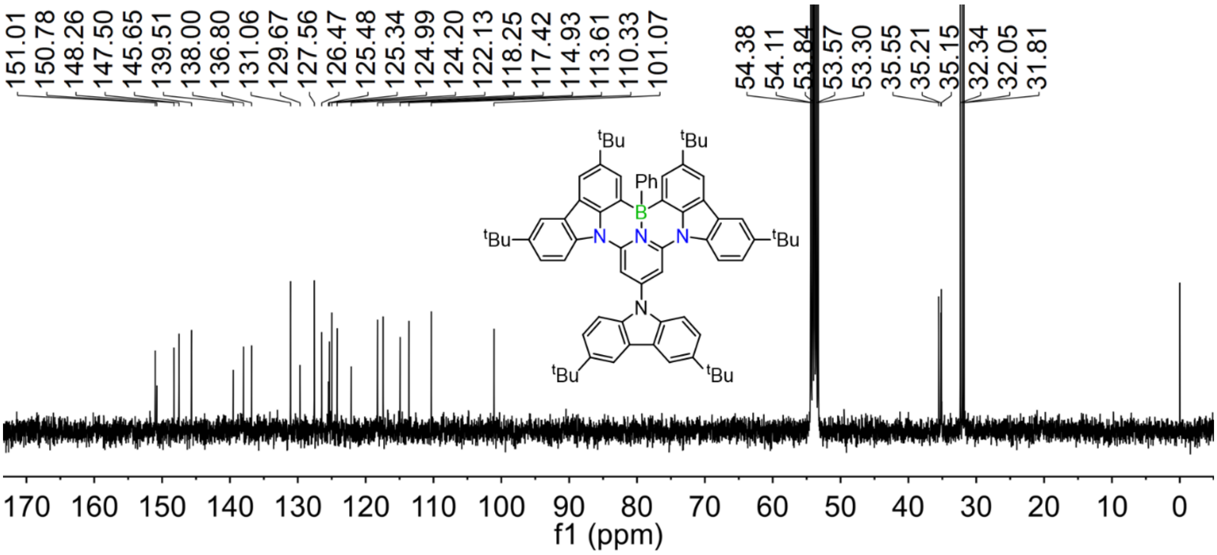

Figure S6.5 ^{13}C NMR spectrum of **TCz-BN1** (101 MHz, CD_2Cl_2)

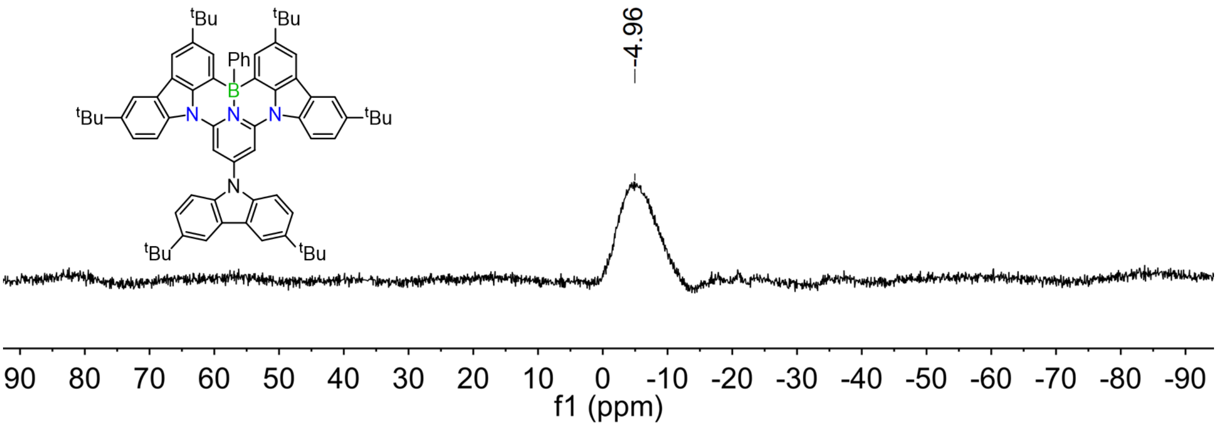

Figure S6.6 ^{11}B NMR spectrum of **TCz-BN1** (128 MHz, CD_2Cl_2)

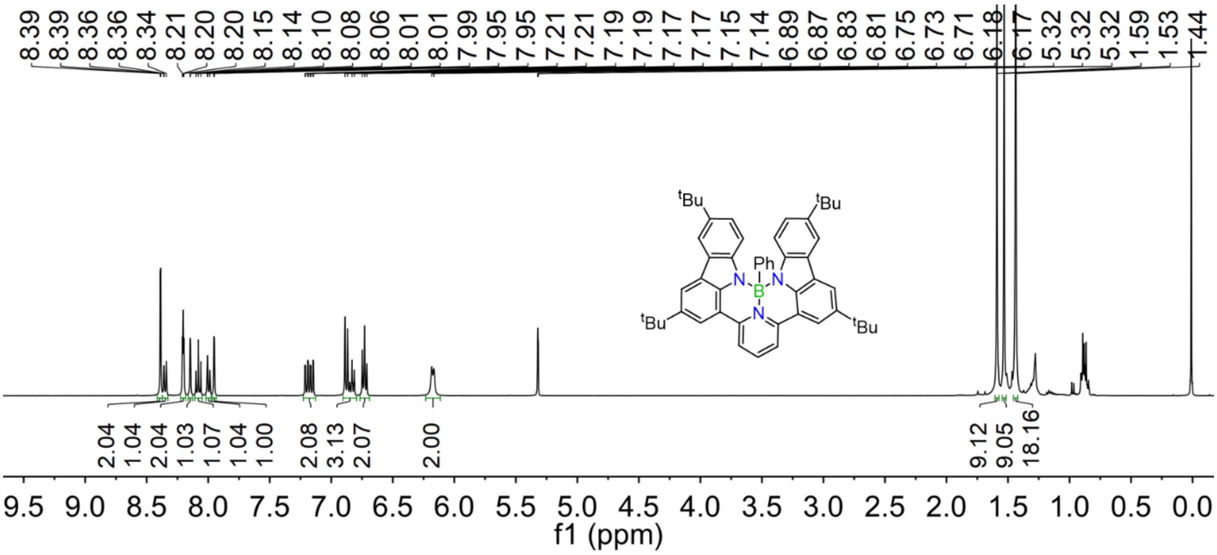

Figure S6.7 ^{1}H NMR spectrum of **BN2** (400 MHz, CD_2Cl_2)

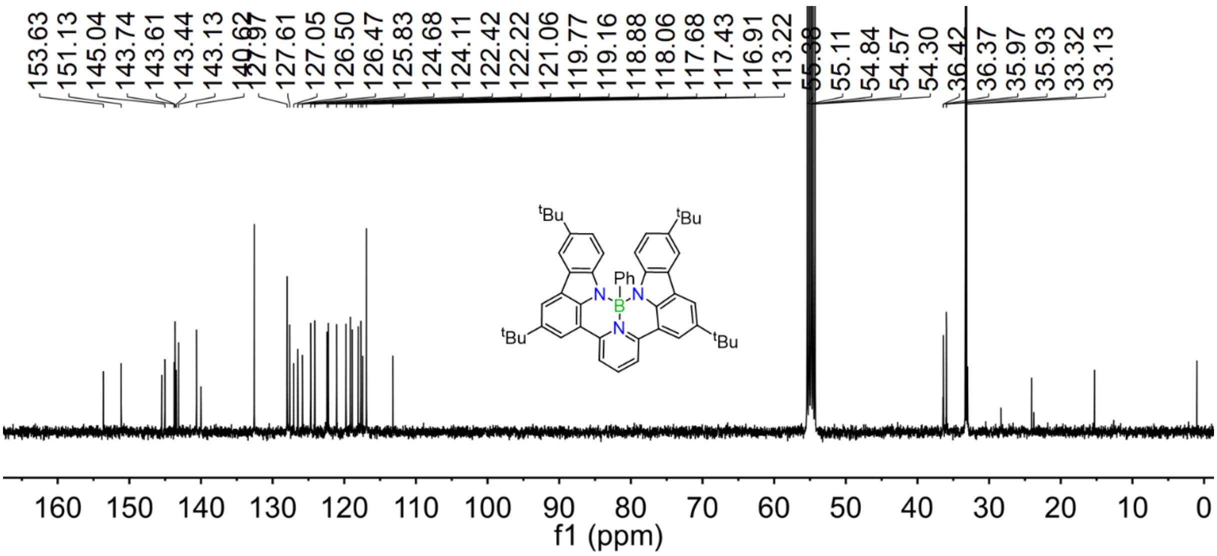

Figure S6.8 ^{13}C NMR spectrum of **BN2** (101 MHz, CD_2Cl_2)

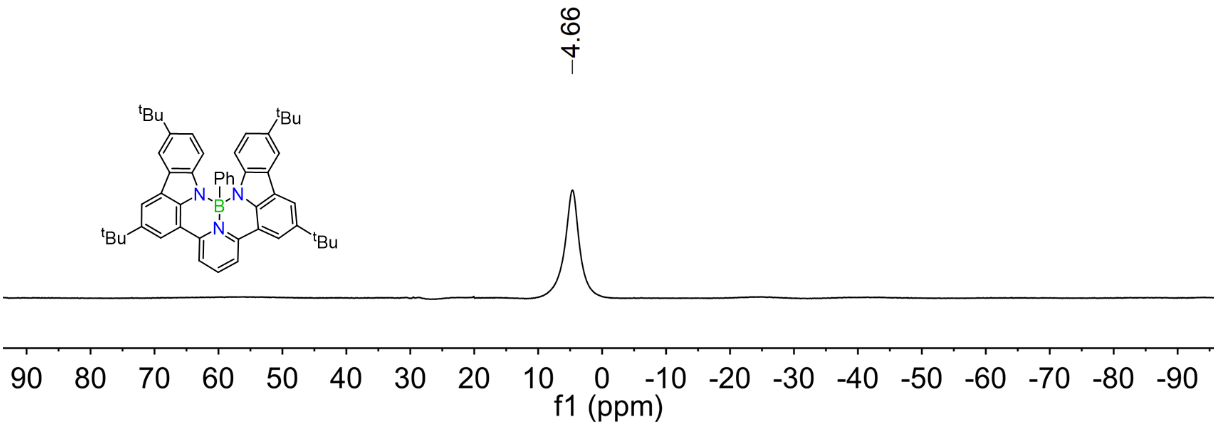

Figure S6.9 ^{11}B NMR spectrum of **BN2** (128 MHz, CD_2Cl_2)

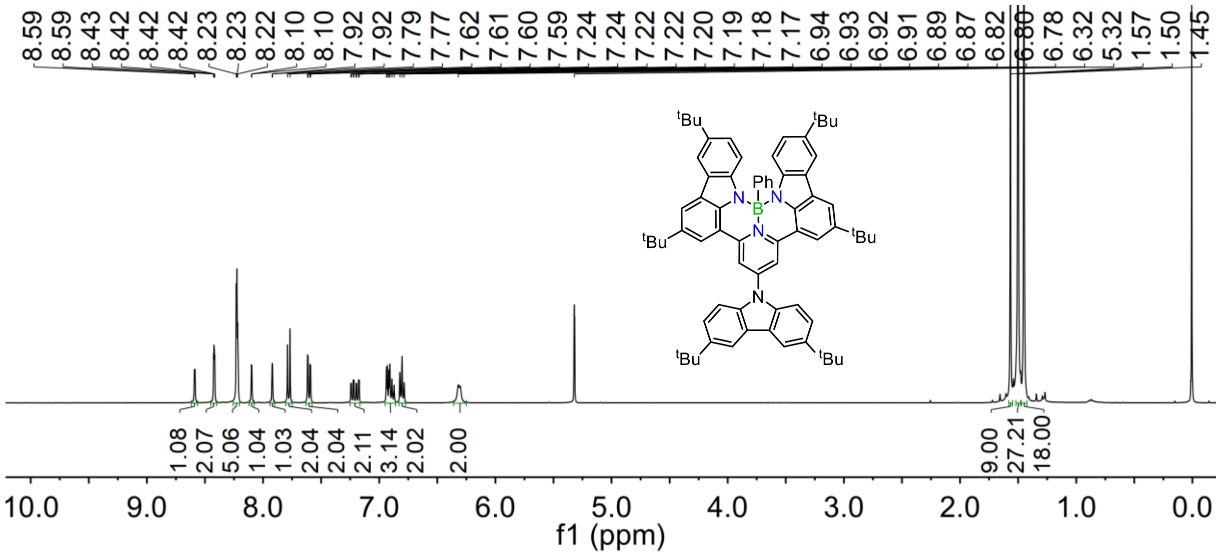

Figure S6.10 ^{1}H NMR spectrum of **TCz-BN2** (400 MHz, CD_2Cl_2)

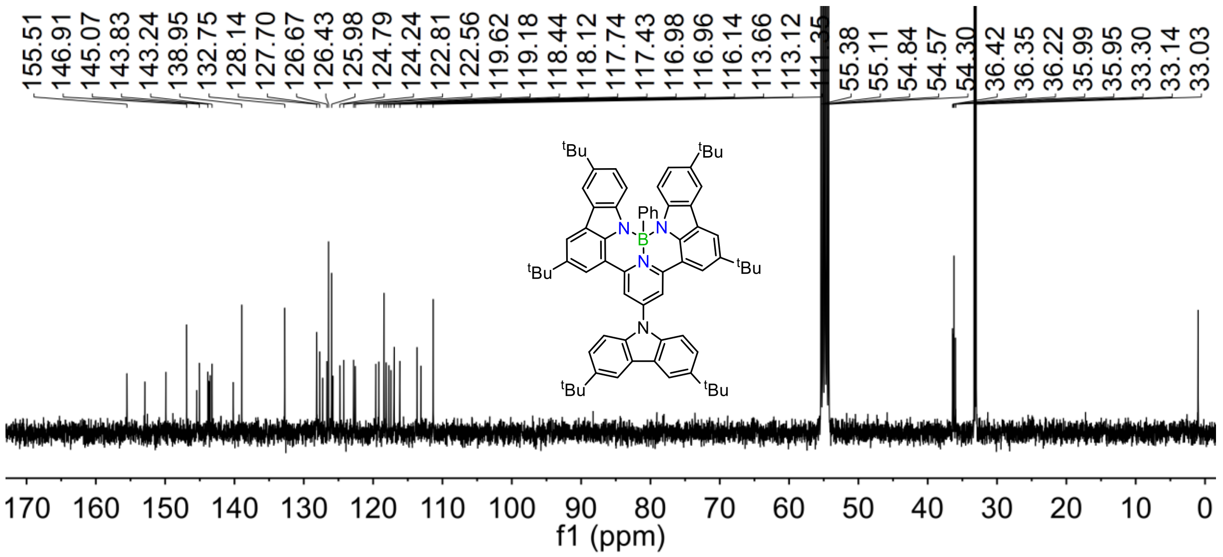

Figure S6.11 ^{13}C NMR spectrum of **TCz-BN2** (101 MHz, CD_2Cl_2)

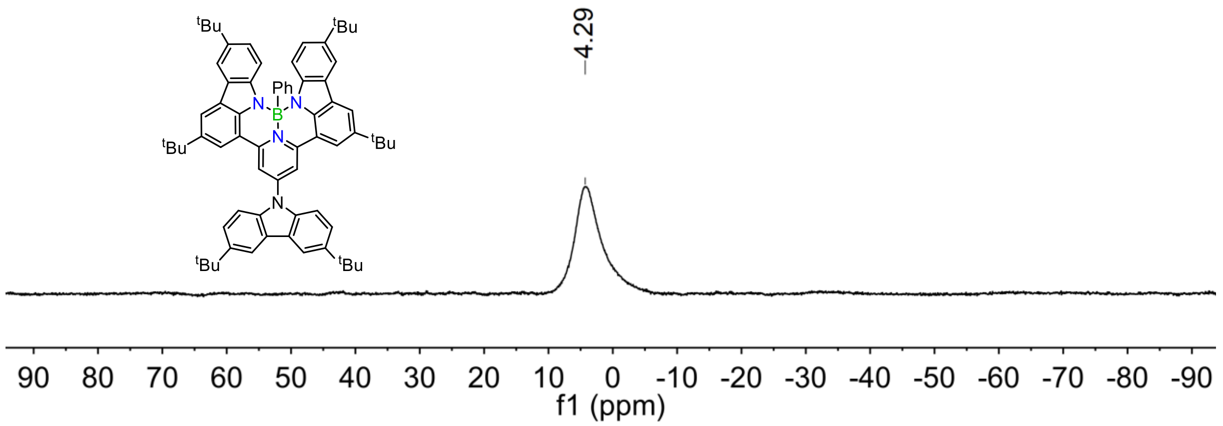

Figure S6.12 ^{11}B NMR spectrum of **TCz-BN2** (128 MHz, CD_2Cl_2)

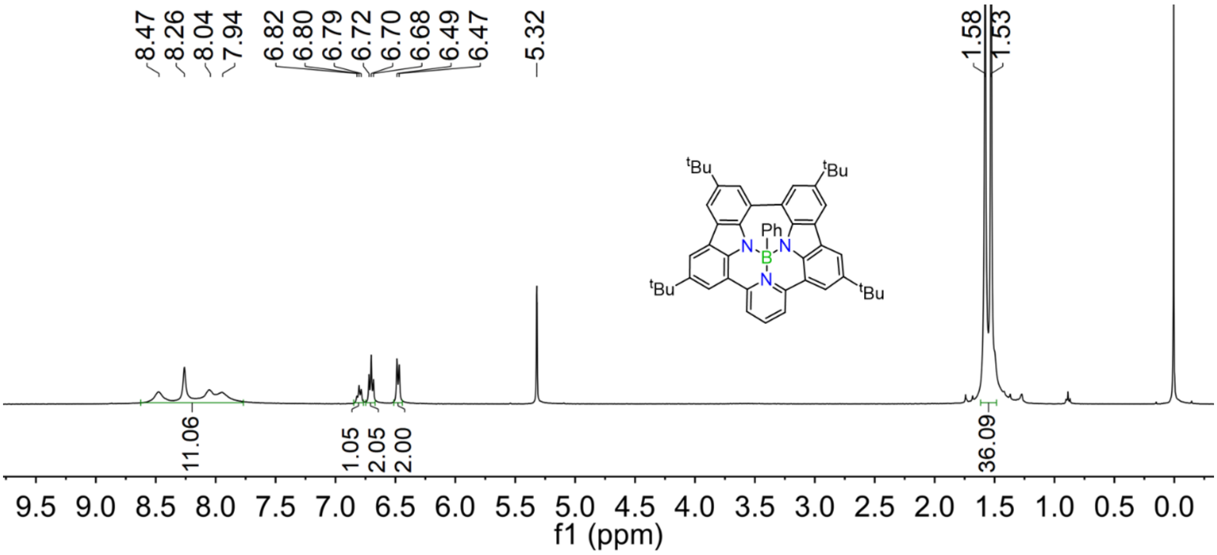

Figure S6.13 ^{1}H NMR spectrum of **BN3** (400 MHz, CD_2Cl_2)

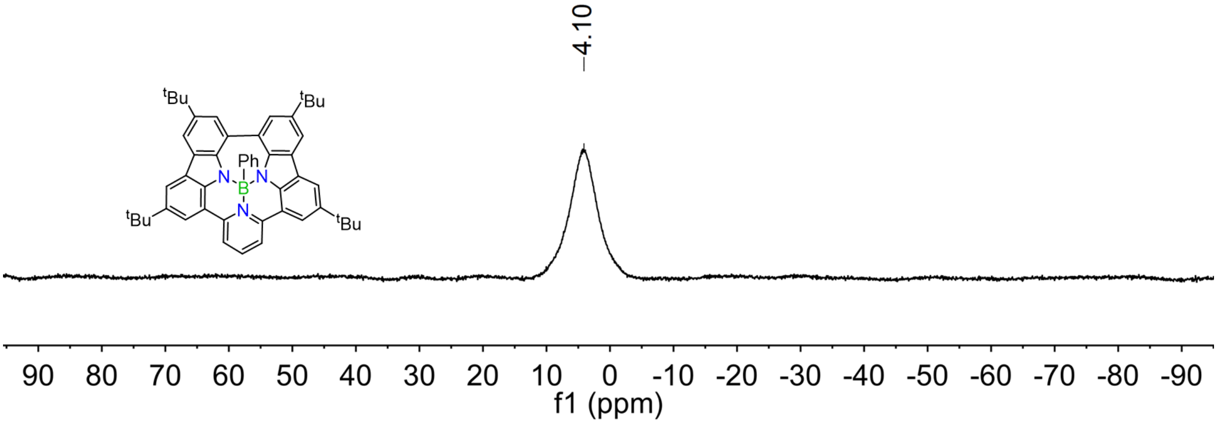

Figure S6.14 ^{11}B NMR spectrum of **BN3** (128 MHz, CD_2Cl_2)

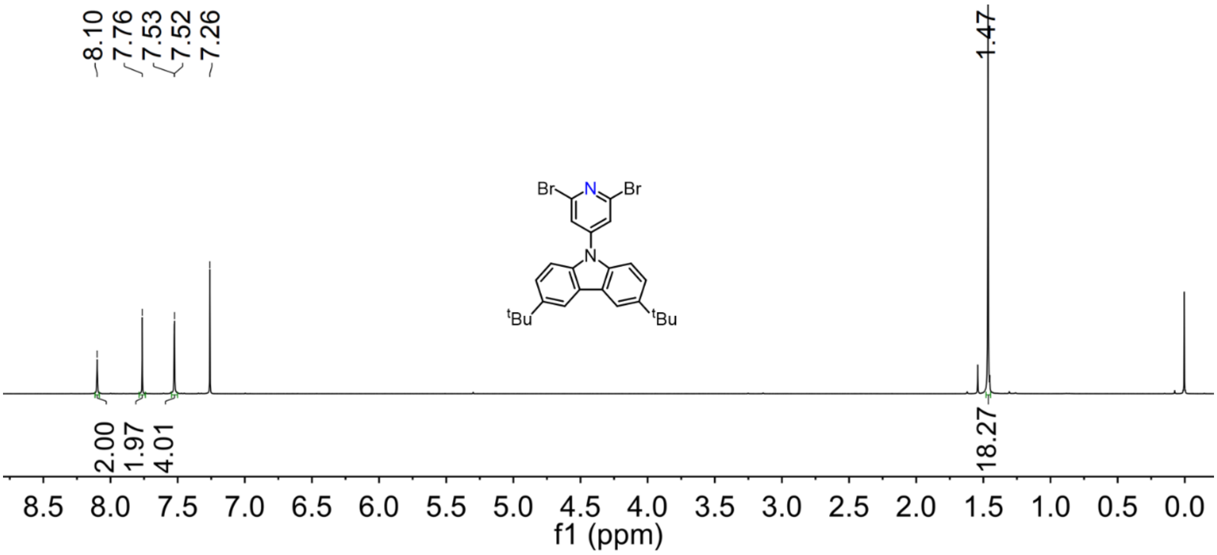

Figure S6.15 ^{11}H NMR spectrum of **1a** (400 MHz, $CDCl_3$)

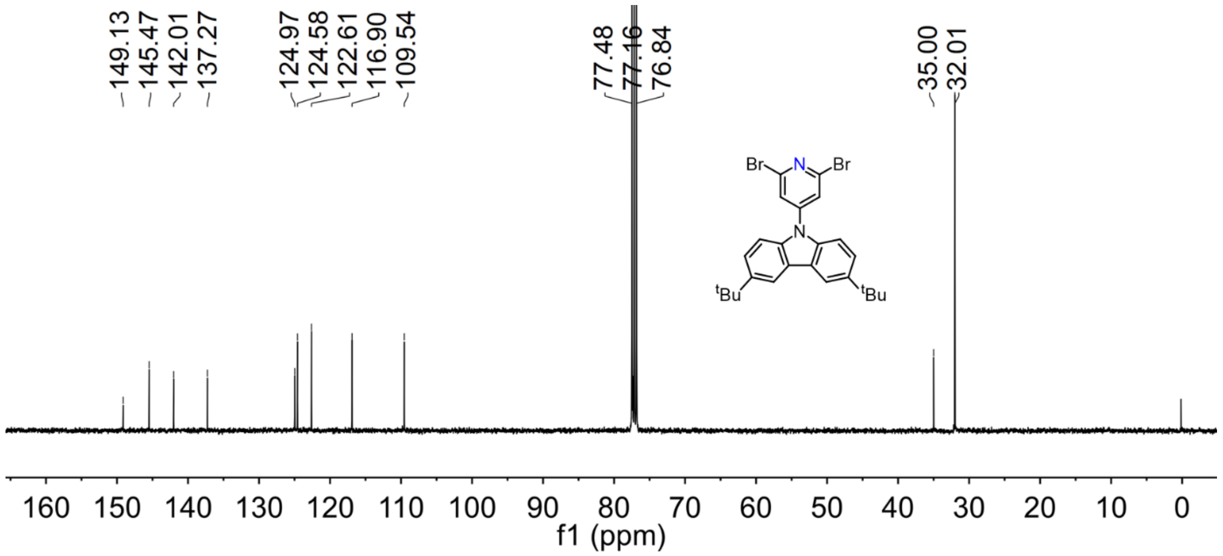

Figure S6.16 ^{13}C NMR spectrum of **11** (101 MHz, $CDCl_3$)

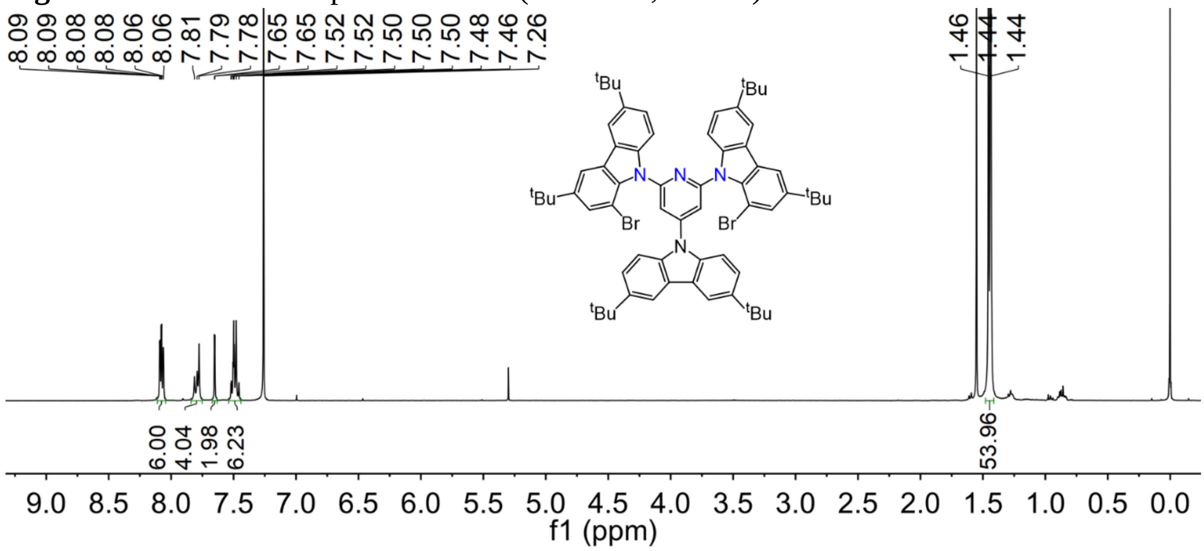

Figure S6.17 ^{1}H NMR spectrum of **1c** (400 MHz, $CDCl_3$)

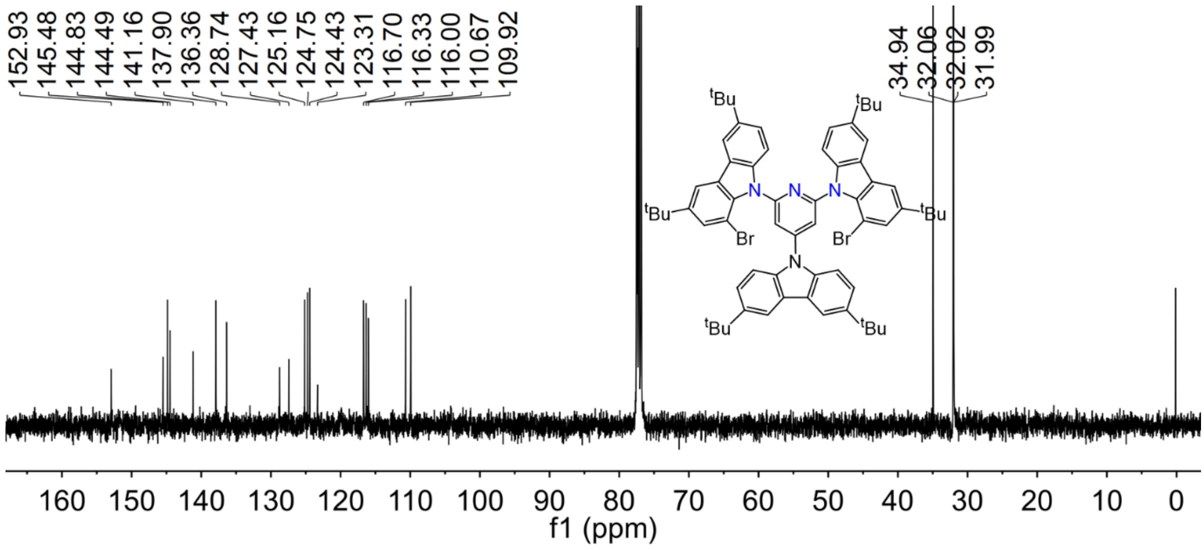

Figure S6.18 ^{13}C NMR spectrum of **1c** (101 MHz, $CDCl_3$)

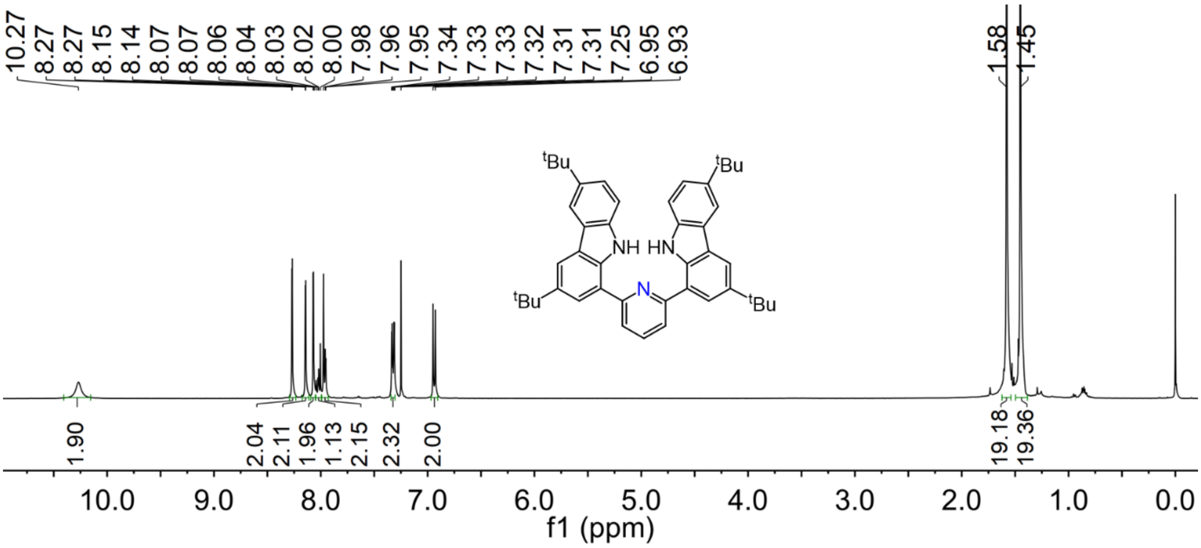

Figure S6.19 ^{1}H NMR spectrum of **2b** (400 MHz, $CDCl_3$)

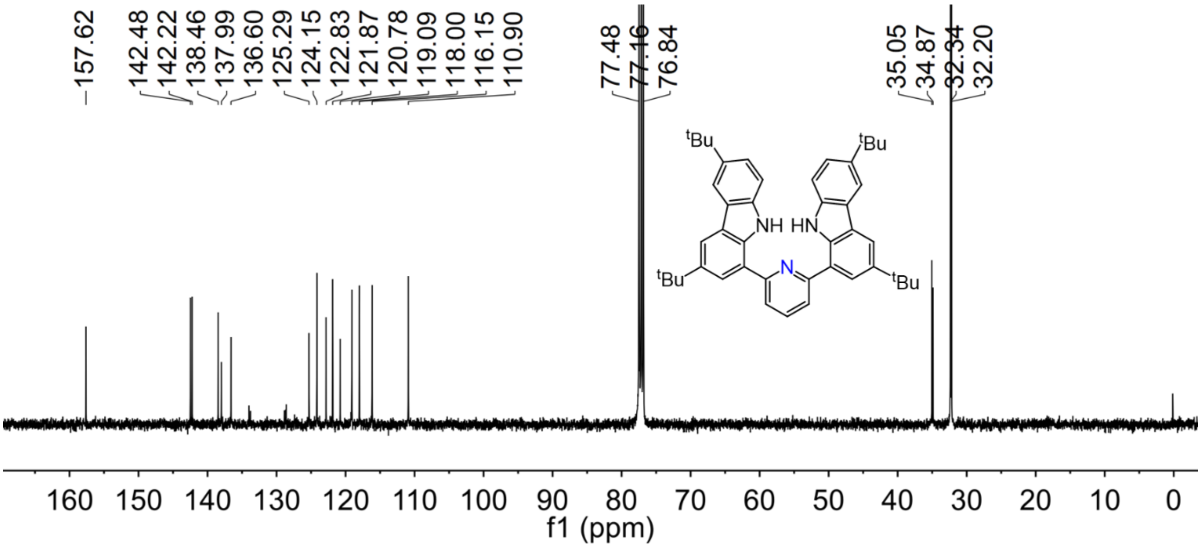

Figure S6.20 ^{13}C NMR spectrum of **2b** (101 MHz, $CDCl_3$)

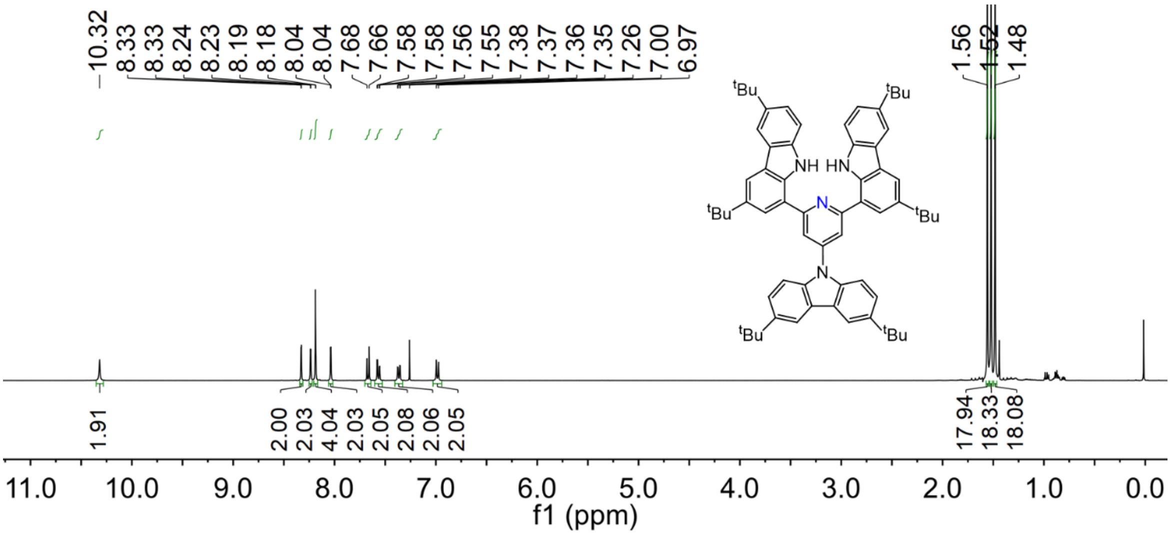

Figure S6.21 ^{1}H NMR spectrum of **2c** (400 MHz, $CDCl_3$)

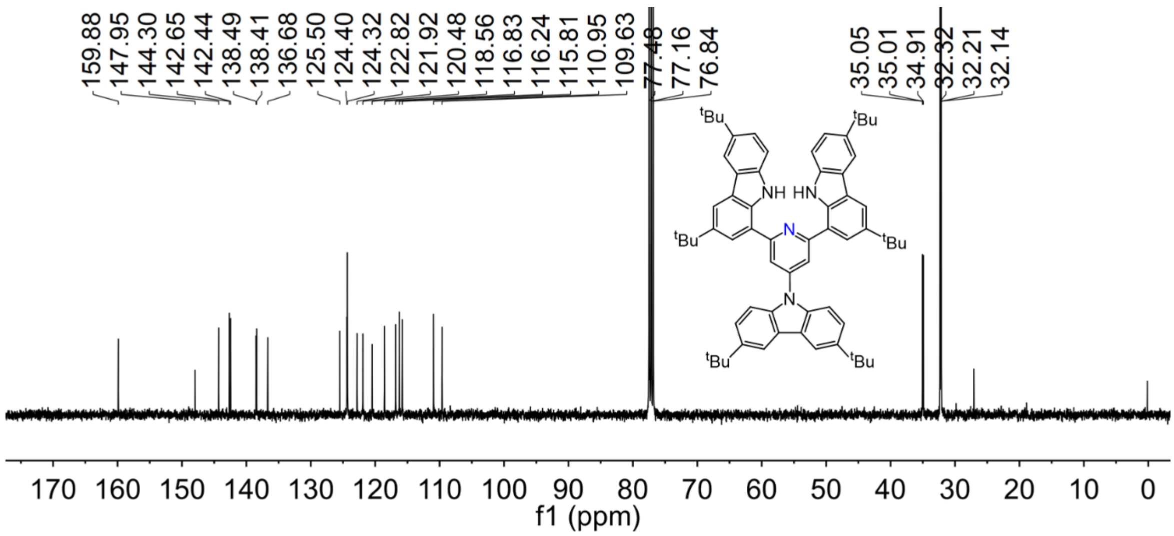

Figure S6.22 ^{13}C NMR spectrum of **2c** (101 MHz, $CDCl_3$)

S8 Supplementary figures and tables

Table S7.1 X-Ray crystallographic data and structure refinement for **BN1** and **TCz-BN1**.

Sample	**BN1**	**TCz-BN1**
Empirical formula	$C_{51}H_{56}BN_3Cl_2$	$C_{72}H_{79}BN_4 Cl_2$
Formula weight	804.70	1082.10
Temperature	296(2) K	180(2) K
Wavelength	0.71073 Å	0.71073 Å
Crystal system	Monoclinic	Monoclinic
Space group	P2(1)/n	P2(1)/n
Unit cell dimensions	a = 9.5765(4) Å<br>b = 10.9386(4) Å<br>c = 42.1549(16)Å<br>α = 90°<br>β = 93.5484(13)°<br>γ = 90°	a = 10.3197(9) Å<br>b = 30.507(3) Å<br>c = 19.381(2) Å<br>α = 90°<br>β = 104.430(3)°<br>γ = 90°
Volume	4407.4(3) $Å^3$	5909.1(10) $Å^3$
Z	4	4
Density (calculated)	1.213 Mg/m^3	1.216 Mg/m^3
Absorption coefficient	0.186 mm^{-1}	0.157 mm^{-1}
F(000)	1712	2312
Theta range for data collection	0.968 to 26.867°	2.270 to 24.793°
Index ranges	-12<=h<=12<br>-13<=k<=13<br>-53<=l<=53	-12<=h<=12<br>-36<=k<=30<br>-22<=l<=22
Reflections collected	48104	59165
Independent reflections	9472 [R(int) = 0.0490]	10116 [R(int) = 0.1696]

Completeness to theta = 25.242°	100.0 %	99.6 %
Absorption correction	Semi-empirical from equivalents	
Refinement method	Full-matrix least-squares on F^2	
Data/restraints/parameters	9472 / 73 / 535	10116 / 31 / 718
Goodness-of-fit on F^2	1.031	1.045
Final R indices [I>2sigma(I)]	R1 = 0.1096<br>wR2 = 0.3132	R1 = 0.1071<br>wR2 = 0.2702
R indices (all data)	R1 = 0.1371<br>wR2 = 0.3416	R1 = 0.2045<br>wR2 = 0.3333
Extinction coefficient	n/a	n/a
Largest diff. peak and hole (e.Å^{-3})	2.054 and -1.376	0.797 and -1.003

Table S7.2 X-Ray crystallographic data and structure refinement for **BN2** and **BN3**.

Sample	**BN2**	**BN3**
Empirical formula	$C_{51}H_{54}BN_3$	$C_{52}H_{54}BN_3Cl_2$
Formula weight	719.82	802.69
Temperature	180(2) K	180(2) K
Wavelength	0.71073 Å	0.71073 Å
Crystal system	Monoclinic	Triclinic
Space group	C2/c	P-1
Unit cell dimensions	a = 15.4761(18) Å<br>b = 32.994(3) Å<br>c = 18.0610(18) Å<br>α = 90°<br>β = 87.00°<br>γ = 90°	a = 11.2074(8) Å<br>b = 13.1456(9) Å<br>c = 15.4247(12) Å<br>α = 72.412(3)°<br>β = 80.497(3)°<br>γ = 85.153(3)°
Volume	9209.6(16) Å^3	2135.1(3) Å^3
Z	8	43
Density (calculated)	1.037 Mg/m^3	1.249 Mg/m^3
Absorption coefficient	0.060 mm^{-1}	0.192 mm^{-1}
F(000)	3080	852
Theta range for data collection	2.258 to 25.499°	2.438 to 27.537°
Index ranges	-18<=h<=18<br>-39<=k<=39<br>-21<=l<=21	-13<=h<=14<br>-16<=k<=17<br>-20<=l<=20
Reflections collected	42064	28132
Independent reflections	7463 [R(int) = 0.1133]	9808 [R(int) = 0.0383]
Completeness to theta = 25.242°	86.5 %	99.7 %
Absorption correction	Semi-empirical from equivalents	

Refinement method	Full-matrix least-squares on F^2	
Data/restraints/parameters	7463 / 40 / 507	9808 / 0 / 532
Goodness-of-fit on F^2	0.970	1.044
Final R indices [I>2sigma(I)]	R1 = 0.0839<br>wR2 = 0.2144	R1 = 0.0529<br>wR2 = 0.1267
R indices (all data)	R1 = 0.1619<br>wR2 = 0.2385	R1 = 0.0844<br>wR2 = 0.1422
Extinction coefficient	n/a	n/a
Largest diff. peak and hole (e.Å^{-3})	0.629 and -0.442	0.448 and -0.535

Table S7.3 Atomic coordinates (x 10^4) and equivalent isotropic displacement parameters (Å^2 x 10^3) for **BN1**. U(eq) is defined as one third of the trace of the orthogonalized U^{ij} tensor.

	x	y	z	U(eq)
Cl(1)	4350(3)	1549(2)	222(1)	83(1)
N(2)	2384(3)	2642(3)	1715(1)	15(1)
N(1)	2992(3)	2664(3)	2267(1)	14(1)
C(29)	5851(4)	4613(3)	2540(1)	14(1)
N(3)	1767(3)	2618(3)	1163(1)	17(1)
C(56)	3867(4)	4392(3)	652(1)	19(1)
C(34)	4023(4)	3266(3)	3092(1)	18(1)
C(52)	4035(4)	3616(3)	1305(1)	16(1)
C(22)	1988(4)	2476(3)	2020(1)	15(1)
C(40)	4807(4)	1594(3)	1616(1)	16(1)
C(26)	5876(4)	4433(3)	1968(1)	16(1)
C(30)	4668(4)	3864(3)	2525(1)	14(1)
C(20)	1376(4)	2481(3)	1471(1)	16(1)
C(42)	628(4)	2163(4)	2084(1)	21(1)
C(53)	5077(4)	4318(3)	1173(1)	18(1)
C(32)	3857(4)	3277(3)	2761(1)	15(1)
C(33)	2078(4)	1703(3)	2770(1)	19(1)
C(27)	7751(4)	5751(3)	2258(1)	19(1)
Cl(19)	5536(4)	-306(2)	638(1)	127(1)
C(24)	4140(4)	3449(3)	2230(1)	13(1)
C(25)	4709(4)	3680(3)	1943(1)	13(1)
C(31)	2854(4)	2519(3)	2601(1)	16(1)
C(28)	6457(4)	4920(3)	2259(1)	16(1)
C(36)	2279(4)	1697(4)	3101(1)	22(1)

C(19)	-11(4)	2219(4)	1529(1)	21(1)
C(48)	2791(4)	3698(3)	772(1)	17(1)
C(43)	-199(4)	1678(4)	798(1)	22(1)
C(51)	2916(4)	3351(3)	1090(1)	16(1)
C(41)	-365(4)	2050(4)	1836(1)	24(1)
C(54)	5023(4)	4712(4)	853(1)	19(1)
C(50)	932(4)	2445(3)	875(1)	18(1)
C(46)	958(4)	3078(4)	323(1)	21(1)
C(37)	4305(4)	516(4)	1739(1)	24(1)
C(39)	6085(4)	1507(4)	1471(1)	26(1)
C(47)	1544(4)	3123(3)	634(1)	18(1)
C(35)	3244(4)	2482(4)	3268(1)	23(1)
C(44)	-756(4)	1662(4)	486(1)	25(1)
C(45)	-204(5)	2363(4)	244(1)	32(1)
C(38)	5020(5)	-591(4)	1724(1)	32(1)
C(55)	6274(4)	5418(4)	736(1)	24(1)
C(1)	6259(5)	-649(4)	1577(1)	30(1)
B(1)	4056(4)	2928(4)	1642(1)	15(1)
C(3)	6796(5)	405(4)	1450(1)	32(1)
C(4)	-840(6)	2331(5)	-98(1)	41(1)
C(5)	6542(5)	6572(4)	938(1)	37(1)
C(6)	7411(6)	6828(5)	2034(1)	42(1)
C(7)	7577(5)	4606(5)	768(1)	39(1)
C(8)	3488(5)	2373(6)	3629(1)	44(1)
C(9)	6032(6)	5810(6)	388(1)	48(2)
C(10)	2127(6)	2241(6)	3792(1)	45(1)
C(11)	8167(6)	6274(6)	2585(1)	48(1)
C(12)	8975(6)	5040(6)	2139(2)	59(2)
C(13)	4303(8)	1157(10)	3702(2)	92(3)
C(14)	4562(13)	1033(8)	617(2)	100(3)
C(15)	346(10)	1869(13)	-308(2)	119(4)
C(16)	4395(9)	3382(8)	3768(2)	76(2)
C(17)	-1106(8)	3551(7)	-225(2)	68(1)
C(18)	-1863(8)	1277(7)	-164(2)	70(1)

Table S7.4 Bond lengths [Å] and angles [°] for **BN1**.

Cl(1)-C(14)	1.758(8)
N(2)-C(22)	1.374(5)
N(2)-C(20)	1.377(4)
N(2)-B(1)	1.679(5)
N(1)-C(22)	1.388(4)

N(1)-C(24)	1.412(4)
N(1)-C(31)	1.433(4)
C(29)-C(28)	1.394(5)
C(29)-C(30)	1.396(5)
N(3)-C(20)	1.383(5)
N(3)-C(51)	1.410(5)
N(3)-C(50)	1.426(4)
C(56)-C(54)	1.396(5)
C(56)-C(48)	1.399(5)
C(34)-C(35)	1.381(5)
C(34)-C(32)	1.394(5)
C(52)-C(51)	1.389(5)
C(52)-C(53)	1.400(5)
C(52)-B(1)	1.606(5)
C(22)-C(42)	1.389(5)
C(40)-C(37)	1.385(5)
C(40)-C(39)	1.405(5)
C(40)-B(1)	1.633(5)
C(26)-C(25)	1.388(5)
C(26)-C(28)	1.418(5)
C(30)-C(24)	1.390(5)
C(30)-C(32)	1.449(5)
C(20)-C(19)	1.394(5)
C(42)-C(41)	1.373(5)
C(53)-C(54)	1.416(5)
C(32)-C(31)	1.409(5)
C(33)-C(31)	1.386(5)
C(33)-C(36)	1.394(5)
C(27)-C(12)	1.518(6)
C(27)-C(11)	1.524(6)
C(27)-C(6)	1.531(6)
C(27)-C(28)	1.537(5)
Cl(19)-C(14)	1.735(8)
C(24)-C(25)	1.381(5)
C(25)-B(1)	1.607(5)
C(36)-C(35)	1.416(5)
C(19)-C(41)	1.372(5)
C(48)-C(51)	1.394(5)
C(48)-C(47)	1.440(5)
C(43)-C(44)	1.390(5)
C(43)-C(50)	1.392(5)
C(54)-C(55)	1.533(5)
C(50)-C(47)	1.414(5)
C(46)-C(45)	1.384(6)

C(46)-C(47)	1.394(5)
C(37)-C(38)	1.394(6)
C(39)-C(3)	1.391(6)
C(35)-C(8)	1.534(5)
C(44)-C(45)	1.403(6)
C(45)-C(4)	1.534(7)
C(38)-C(1)	1.373(6)
C(55)-C(7)	1.531(6)
C(55)-C(9)	1.533(6)
C(55)-C(5)	1.536(6)
C(1)-C(3)	1.383(6)
C(4)-C(17)	1.453(8)
C(4)-C(18)	1.526(8)
C(4)-C(15)	1.566(11)
C(8)-C(16)	1.500(9)
C(8)-C(10)	1.516(7)
C(8)-C(13)	1.563(10)

C(22)-N(2)-C(20)	117.3(3)
C(22)-N(2)-B(1)	121.2(3)
C(20)-N(2)-B(1)	121.3(3)
C(22)-N(1)-C(24)	121.1(3)
C(22)-N(1)-C(31)	128.3(3)
C(24)-N(1)-C(31)	107.2(3)
C(28)-C(29)-C(30)	118.8(3)
C(20)-N(3)-C(51)	121.7(3)
C(20)-N(3)-C(50)	128.1(3)
C(51)-N(3)-C(50)	107.1(3)
C(54)-C(56)-C(48)	119.4(3)
C(35)-C(34)-C(32)	120.6(3)
C(51)-C(52)-C(53)	113.4(3)
C(51)-C(52)-B(1)	116.4(3)
C(53)-C(52)-B(1)	129.5(3)
N(2)-C(22)-N(1)	117.6(3)
N(2)-C(22)-C(42)	122.0(3)
N(1)-C(22)-C(42)	120.4(3)
C(37)-C(40)-C(39)	115.9(4)
C(37)-C(40)-B(1)	124.9(3)
C(39)-C(40)-B(1)	119.2(3)
C(25)-C(26)-C(28)	124.0(3)
C(24)-C(30)-C(29)	118.8(3)
C(24)-C(30)-C(32)	106.8(3)
C(29)-C(30)-C(32)	134.1(3)
N(2)-C(20)-N(3)	117.9(3)

N(2)-C(20)-C(19)	121.9(3)
N(3)-C(20)-C(19)	120.2(3)
C(41)-C(42)-C(22)	119.3(3)
C(52)-C(53)-C(54)	124.0(3)
C(34)-C(32)-C(31)	120.4(3)
C(34)-C(32)-C(30)	131.1(3)
C(31)-C(32)-C(30)	108.1(3)
C(31)-C(33)-C(36)	118.1(3)
C(12)-C(27)-C(11)	109.0(4)
C(12)-C(27)-C(6)	109.3(4)
C(11)-C(27)-C(6)	107.6(4)
C(12)-C(27)-C(28)	109.7(3)
C(11)-C(27)-C(28)	112.7(3)
C(6)-C(27)-C(28)	108.5(3)
C(25)-C(24)-C(30)	125.7(3)
C(25)-C(24)-N(1)	124.0(3)
C(30)-C(24)-N(1)	110.1(3)
C(24)-C(25)-C(26)	113.7(3)
C(24)-C(25)-B(1)	116.4(3)
C(26)-C(25)-B(1)	129.5(3)
C(33)-C(31)-C(32)	120.3(3)
C(33)-C(31)-N(1)	131.4(3)
C(32)-C(31)-N(1)	107.7(3)
C(29)-C(28)-C(26)	118.9(3)
C(29)-C(28)-C(27)	121.6(3)
C(26)-C(28)-C(27)	119.5(3)
C(33)-C(36)-C(35)	122.5(3)
C(41)-C(19)-C(20)	119.1(3)
C(51)-C(48)-C(56)	118.3(3)
C(51)-C(48)-C(47)	106.9(3)
C(56)-C(48)-C(47)	134.5(3)
C(44)-C(43)-C(50)	118.2(4)
C(52)-C(51)-C(48)	125.9(3)
C(52)-C(51)-N(3)	124.0(3)
C(48)-C(51)-N(3)	110.0(3)
C(19)-C(41)-C(42)	120.3(4)
C(56)-C(54)-C(53)	118.9(3)
C(56)-C(54)-C(55)	122.5(3)
C(53)-C(54)-C(55)	118.5(3)
C(43)-C(50)-C(47)	120.1(3)
C(43)-C(50)-N(3)	131.6(3)
C(47)-C(50)-N(3)	107.9(3)
C(45)-C(46)-C(47)	121.1(4)
C(40)-C(37)-C(38)	122.7(4)

C(3)-C(39)-C(40)	122.0(4)
C(46)-C(47)-C(50)	119.7(3)
C(46)-C(47)-C(48)	132.2(4)
C(50)-C(47)-C(48)	108.0(3)
C(34)-C(35)-C(36)	117.9(3)
C(34)-C(35)-C(8)	121.8(4)
C(36)-C(35)-C(8)	120.1(4)
C(43)-C(44)-C(45)	122.8(4)
C(46)-C(45)-C(44)	117.9(4)
C(46)-C(45)-C(4)	120.3(4)
C(44)-C(45)-C(4)	121.7(4)
C(1)-C(38)-C(37)	120.0(4)
C(7)-C(55)-C(54)	109.2(3)
C(7)-C(55)-C(9)	108.8(4)
C(54)-C(55)-C(9)	112.0(3)
C(7)-C(55)-C(5)	108.7(4)
C(54)-C(55)-C(5)	110.0(3)
C(9)-C(55)-C(5)	108.1(4)
C(38)-C(1)-C(3)	119.3(4)
C(52)-B(1)-C(25)	116.2(3)
C(52)-B(1)-C(40)	110.0(3)
C(25)-B(1)-C(40)	111.0(3)
C(52)-B(1)-N(2)	106.9(3)
C(25)-B(1)-N(2)	106.2(3)
C(40)-B(1)-N(2)	105.9(3)
C(1)-C(3)-C(39)	120.1(4)
C(17)-C(4)-C(18)	122.1(6)
C(17)-C(4)-C(45)	112.0(5)
C(18)-C(4)-C(45)	113.6(5)
C(17)-C(4)-C(15)	101.9(7)
C(18)-C(4)-C(15)	97.7(6)
C(45)-C(4)-C(15)	106.2(5)
C(16)-C(8)-C(10)	112.8(5)
C(16)-C(8)-C(35)	112.3(5)
C(10)-C(8)-C(35)	112.0(4)
C(16)-C(8)-C(13)	106.2(6)
C(10)-C(8)-C(13)	105.2(5)
C(35)-C(8)-C(13)	107.7(5)
Cl(19)-C(14)-Cl(1)	110.4(4)

Symmetry transformations used to generate equivalent atoms: #1 -x+1, -y+1, -z+1

Table S7.5 Anisotropic displacement parameters (Å^2 x 10^3) for **BN1**; the anisotropic

displacement factor exponent takes the form: $-2\pi^2$ [h^2 $a^{*2}U^{11}$ + ... + 2hka* b* U^{12}]

	U11	U22	U33	U23	U13	U12
Cl(1)	105(2)	79(1)	68(1)	-1(1)	17(1)	25(1)
N(2)	15(1)	16(1)	13(1)	1(1)	-2(1)	-1(1)
N(1)	15(1)	17(1)	10(1)	2(1)	-1(1)	-5(1)
C(29)	17(2)	13(2)	11(2)	0(1)	-4(1)	-2(1)
N(3)	18(2)	24(2)	9(1)	1(1)	-2(1)	-4(1)
C(56)	22(2)	22(2)	12(2)	5(1)	-1(1)	-1(2)
C(34)	18(2)	24(2)	11(2)	-1(1)	-4(1)	-2(1)
C(52)	17(2)	17(2)	13(2)	1(1)	-1(1)	-1(1)
C(22)	19(2)	16(2)	10(2)	1(1)	-1(1)	-1(1)
C(40)	16(2)	19(2)	14(2)	0(1)	-6(1)	-2(1)
C(26)	19(2)	15(2)	14(2)	3(1)	0(1)	-3(1)
C(30)	18(2)	13(2)	11(2)	2(1)	-1(1)	3(1)
C(20)	19(2)	18(2)	11(2)	2(1)	-2(1)	-2(1)
C(42)	17(2)	31(2)	13(2)	2(2)	1(1)	-5(2)
C(53)	19(2)	21(2)	13(2)	1(1)	-2(1)	-2(1)
C(32)	18(2)	14(2)	13(2)	-1(1)	1(1)	-1(1)
C(33)	19(2)	23(2)	14(2)	1(1)	0(1)	-4(1)
C(27)	17(2)	18(2)	21(2)	2(1)	-1(1)	-6(1)
Cl(19)	219(4)	70(1)	86(2)	-7(1)	-36(2)	49(2)
C(24)	15(2)	12(2)	13(2)	3(1)	-1(1)	-2(1)
C(25)	15(2)	14(2)	11(2)	3(1)	-2(1)	-2(1)
C(31)	17(2)	21(2)	9(2)	0(1)	-2(1)	1(1)
C(28)	16(2)	14(2)	18(2)	2(1)	-4(1)	-2(1)
C(36)	22(2)	30(2)	13(2)	5(2)	2(1)	-9(2)
C(19)	16(2)	31(2)	16(2)	1(2)	-5(1)	-4(2)
C(48)	19(2)	22(2)	11(2)	3(1)	-2(1)	0(1)
C(43)	23(2)	28(2)	15(2)	2(2)	-1(1)	-6(2)
C(51)	19(2)	16(2)	13(2)	3(1)	1(1)	-1(1)
C(41)	14(2)	37(2)	21(2)	2(2)	1(1)	-5(2)
C(54)	21(2)	24(2)	13(2)	3(1)	2(1)	-2(2)
C(50)	20(2)	24(2)	10(2)	1(1)	-4(1)	-2(1)
C(46)	22(2)	29(2)	12(2)	3(1)	-3(1)	-1(2)
C(37)	23(2)	21(2)	30(2)	5(2)	5(2)	-3(2)
C(39)	21(2)	23(2)	36(2)	4(2)	6(2)	-2(2)
C(47)	20(2)	20(2)	15(2)	2(1)	-1(1)	-1(1)

C(35)	23(2)	35(2)	12(2)	2(2)	-1(1)	-5(2)
C(44)	24(2)	34(2)	17(2)	-3(2)	-6(2)	-6(2)
C(45)	28(2)	38(2)	31(2)	-3(1)	13(1)	0(2)
C(38)	32(2)	18(2)	45(3)	9(2)	2(2)	-1(2)
C(55)	23(2)	34(2)	16(2)	8(2)	1(1)	-6(2)
C(1)	32(2)	18(2)	39(2)	-2(2)	-1(2)	6(2)
B(1)	15(2)	16(2)	12(2)	1(1)	0(1)	-4(1)
C(3)	24(2)	29(2)	44(3)	-1(2)	9(2)	1(2)
C(4)	41(1)	45(1)	38(1)	-1(1)	2(1)	0(1)
C(5)	42(3)	34(2)	37(3)	3(2)	12(2)	-18(2)
C(6)	46(3)	36(3)	42(3)	13(2)	-11(2)	-15(2)
C(7)	28(2)	52(3)	38(3)	4(2)	10(2)	0(2)
C(8)	32(2)	88(4)	13(2)	12(2)	4(2)	-15(2)
C(9)	39(3)	79(4)	25(2)	24(2)	-4(2)	-29(3)
C(10)	48(3)	72(4)	18(2)	4(2)	9(2)	-4(3)
C(11)	48(3)	63(4)	33(3)	0(2)	-4(2)	-34(3)
C(12)	27(3)	51(3)	103(5)	-24(3)	21(3)	-8(2)
C(13)	63(4)	186(10)	28(3)	44(4)	5(3)	54(5)
C(14)	176(10)	66(5)	60(5)	-2(4)	24(6)	47(6)
C(15)	102(7)	215(13)	37(4)	-35(6)	-12(4)	0(8)
C(16)	79(2)	78(2)	69(2)	-4(2)	0(2)	-20(2)
C(17)	78(2)	60(1)	63(2)	15(2)	-11(2)	8(2)
C(18)	70(2)	73(2)	65(2)	-8(2)	-14(2)	-22(2)

Table S7.6 Hydrogen coordinates (x 10^4) and isotropic displacement parameters (Å^2 x 10^3) for **BN1**.

	x	y	z	U(eq)
H(29)	6228	4901	2735	16
H(56)	3813	4637	440	23
H(34)	4664	3791	3195	22
H(26)	6304	4632	1783	19
H(42)	392	2032	2292	25
H(53)	5852	4539	1305	21
H(33)	1441	1175	2666	23
H(36)	1760	1155	3216	26

H(19)	-686	2161	1361	26
H(43)	-573	1189	952	26
H(41)	-1283	1858	1877	28
H(46)	1354	3536	166	25
H(37)	3457	531	1835	29
H(39)	6466	2208	1386	32
H(44)	-1526	1166	435	30
H(38)	4659	-1290	1814	38
H(1)	6731	-1388	1563	36
H(3)	7636	375	1350	39
H(5A)	5720	7075	925	56
H(5B)	7311	7019	859	56
H(5C)	6763	6342	1155	56
H(6A)	7092	6524	1829	63
H(6B)	8237	7314	2015	63
H(6C)	6692	7321	2118	63
H(7A)	7817	4452	989	58
H(7B)	8342	5012	676	58
H(7C)	7390	3845	660	58
H(9A)	5918	5098	255	72
H(9B)	6822	6273	325	72
H(9C)	5204	6304	364	72
H(10A)	1550	2944	3746	68
H(10B)	2324	2176	4017	68
H(10C)	1647	1519	3715	68
H(11A)	7446	6814	2649	72
H(11B)	9028	6719	2576	72
H(11C)	8290	5619	2736	72
H(12A)	9265	4427	2293	89
H(12B)	9739	5588	2110	89
H(12C)	8697	4654	1941	89
H(13A)	3791	485	3606	138
H(13B)	4409	1035	3928	138
H(13C)	5209	1205	3617	138
H(14A)	5033	1656	748	120
H(14B)	3652	887	698	120
H(15A)	1153	2384	-275	178
H(15B)	21	1896	-528	178
H(15C)	590	1044	-250	178
H(16A)	5214	3459	3650	114
H(16B)	4667	3196	3986	114
H(16C)	3882	4137	3758	114
H(17A)	-1922	3884	-135	101
H(17B)	-1257	3509	-452	101

H(17C)	-315	4065	-170	101
H(18A)	-1507	553	-59	105
H(18B)	-1972	1131	-389	105
H(18C)	-2753	1484	-86	105

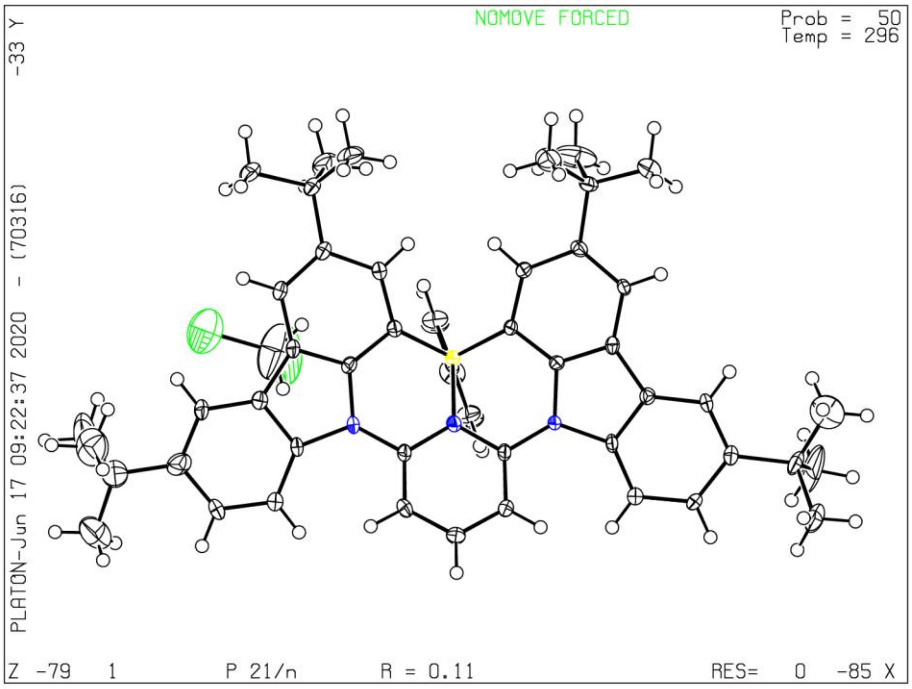

The ORTEP diagram showing the structure of **BN1** with labeling schemes

Table S7.7 Atomic coordinates (x 10^4) and equivalent isotropic displacement parameters ($Å^2$ x 10^3) for **TCz-BN1**. U(eq) is defined as one third of the trace of the orthogonalized U^{ij} tensor.

	x	y	z	U(eq)
N(3)	7217(4)	6749(1)	2727(2)	28(1)
N(1)	3418(4)	6170(1)	3338(2)	30(1)
N(4)	3889(4)	5996(2)	938(2)	33(1)
N(2)	5317(4)	6460(1)	3036(2)	27(1)
C(14)	8100(5)	6873(2)	2295(3)	28(1)
C(31)	4109(5)	6244(2)	2821(3)	27(1)

C(27)	5992(5)	6540(2)	2522(3)	26(1)
C(8)	7983(5)	6739(2)	3435(3)	28(1)
C(12)	9319(5)	6831(2)	3462(3)	29(1)
C(28)	5522(5)	6396(2)	1824(3)	30(1)
C(7)	7466(5)	6671(2)	4022(3)	29(1)
C(30)	3600(5)	6098(2)	2132(3)	31(1)
C(43)	2141(5)	5961(2)	3246(3)	30(1)
C(42)	2110(5)	5753(2)	3885(3)	31(1)
C(33)	4129(5)	6089(2)	4046(3)	30(1)
C(52)	3980(5)	6199(2)	302(3)	31(1)
C(11)	10246(5)	6834(2)	4125(3)	31(1)
C(46)	-189(5)	5537(2)	3391(3)	34(1)
C(29)	4329(5)	6169(2)	1628(3)	28(1)
C(16)	8927(5)	7112(2)	1325(3)	34(1)
C(9)	8435(5)	6690(2)	4671(3)	32(1)
C(34)	3397(5)	5817(2)	4392(3)	32(1)
C(10)	9803(5)	6757(2)	4739(3)	31(1)
C(59)	3396(5)	5567(2)	770(3)	32(1)
C(17)	10222(5)	7160(2)	1751(3)	33(1)
C(15)	7862(5)	6980(2)	1577(3)	32(1)
C(13)	9391(5)	6922(2)	2742(3)	31(1)
C(32)	5380(5)	6257(2)	4347(3)	30(1)
C(1)	5197(5)	7102(2)	3932(3)	32(1)
C(53)	3551(5)	5904(2)	-259(3)	33(1)
C(58)	3172(5)	5505(2)	37(3)	33(1)
C(47)	960(5)	5541(2)	3951(3)	34(1)
C(55)	4323(5)	6738(2)	-521(3)	35(1)
C(36)	5297(5)	5809(2)	5400(3)	36(1)
C(37)	5947(5)	6102(2)	5046(3)	34(1)
C(57)	3958(5)	6446(2)	-1094(3)	35(1)
C(56)	4348(5)	6622(2)	175(3)	36(1)
C(19)	11342(5)	7311(2)	1409(3)	41(2)
C(45)	-134(5)	5760(2)	2770(3)	36(1)
C(48)	-1446(6)	5297(2)	3463(3)	40(1)
C(54)	3543(5)	6027(2)	-951(3)	36(1)
C(44)	1018(5)	5970(2)	2688(3)	35(1)
C(18)	10439(5)	7063(2)	2467(3)	31(1)
C(23)	10766(5)	6737(2)	5481(3)	39(1)
C(35)	3993(5)	5680(2)	5076(3)	36(1)
C(38)	6055(5)	5645(2)	6140(3)	42(2)
C(70)	4026(6)	6591(2)	-1840(3)	42(2)
C(62)	2427(6)	4766(2)	151(3)	44(2)
C(63)	2687(6)	5108(2)	-263(3)	42(2)
C(60)	3211(6)	5229(2)	1207(3)	43(2)

C(2)	3957(5)	7161(2)	4070(3)	41(2)
C(61)	2720(6)	4836(2)	889(3)	48(2)
C(3)	3389(6)	7562(2)	4091(3)	48(2)
C(64)	1822(6)	4335(2)	-186(3)	51(1)
C(24)	12215(6)	6832(3)	5480(3)	57(2)
C(21)	10977(6)	7752(2)	1054(4)	54(2)
C(68)	3152(7)	6982(2)	-2084(3)	58(2)
C(22)	12692(6)	7353(2)	1965(3)	55(2)
C(25)	10331(7)	7070(3)	5964(4)	67(2)
C(4)	4029(7)	7932(2)	3970(4)	57(2)
C(20)	11495(7)	6967(3)	848(4)	62(2)
B(1)	5894(6)	6631(2)	3890(3)	31(2)
C(40)	6430(8)	6031(2)	6651(3)	64(2)
C(69)	5451(7)	6715(3)	-1835(3)	68(2)
C(71)	3583(7)	6224(2)	-2384(3)	59(2)
C(39)	7348(6)	5421(2)	6058(4)	57(2)
C(41)	5269(7)	5310(3)	6448(4)	70(2)
C(6)	5836(7)	7482(2)	3836(4)	68(2)
C(26)	10703(7)	6278(2)	5780(4)	69(2)
C(5)	5258(8)	7894(2)	3848(5)	88(3)
C(65)	2517(11)	3948(3)	240(5)	105(3)
C(51)	-2642(7)	5414(4)	2901(5)	119(4)
C(50)	-1160(8)	4818(3)	3545(7)	128(5)
C(49)	-1777(9)	5435(4)	4145(5)	129(4)
C(67)	2229(14)	4246(3)	-859(5)	142(5)
C(66)	364(10)	4351(4)	-321(8)	171(6)
Cl(2)	193(8)	3767(4)	2475(6)	380(5)
Cl(1)	219(8)	4618(3)	1906(5)	332(3)
C(76)	250(20)	4066(3)	1756(10)	219(8)

Table S7.8 Bond lengths [Å] and angles [°] for **TCz-BN1**.

N(3)-C(27)	1.382(6)
N(3)-C(8)	1.403(6)
N(3)-C(14)	1.432(6)
N(1)-C(31)	1.386(6)
N(1)-C(33)	1.408(6)
N(1)-C(43)	1.434(6)
N(4)-C(29)	1.403(6)
N(4)-C(52)	1.404(7)
N(4)-C(59)	1.412(7)
N(2)-C(27)	1.373(6)

N(2)-C(31)	1.380(6)
N(2)-B(1)	1.695(7)
C(14)-C(15)	1.391(7)
C(14)-C(13)	1.404(7)
C(31)-C(30)	1.381(7)
C(27)-C(28)	1.389(7)
C(8)-C(7)	1.389(7)
C(8)-C(12)	1.396(7)
C(12)-C(11)	1.398(7)
C(12)-C(13)	1.443(7)
C(28)-C(29)	1.382(7)
C(28)-H(28A)	0.9500
C(7)-C(9)	1.399(7)
C(7)-B(1)	1.582(8)
C(30)-C(29)	1.391(7)
C(30)-H(30A)	0.9500
C(43)-C(44)	1.375(7)
C(43)-C(42)	1.398(7)
C(42)-C(47)	1.385(7)
C(42)-C(34)	1.455(7)
C(33)-C(32)	1.377(7)
C(33)-C(34)	1.399(7)
C(52)-C(56)	1.384(7)
C(52)-C(53)	1.395(7)
C(11)-C(10)	1.396(7)
C(11)-H(11A)	0.9500
C(46)-C(47)	1.393(7)
C(46)-C(45)	1.396(7)
C(46)-C(48)	1.526(8)
C(16)-C(15)	1.370(7)
C(16)-C(17)	1.392(7)
C(16)-H(16A)	0.9500
C(9)-C(10)	1.400(7)
C(9)-H(9A)	0.9500
C(34)-C(35)	1.381(7)
C(10)-C(23)	1.532(7)
C(59)-C(60)	1.378(8)
C(59)-C(58)	1.394(7)
C(17)-C(18)	1.381(7)
C(17)-C(19)	1.539(7)
C(15)-H(15A)	0.9500
C(13)-C(18)	1.387(7)
C(32)-C(37)	1.416(7)
C(32)-B(1)	1.614(8)

C(1)-C(6)	1.371(8)
C(1)-C(2)	1.382(7)
C(1)-B(1)	1.617(8)
C(53)-C(54)	1.391(7)
C(53)-C(58)	1.441(8)
C(58)-C(63)	1.384(8)
C(47)-H(47A)	0.9500
C(55)-C(56)	1.388(7)
C(55)-C(57)	1.398(7)
C(55)-H(55A)	0.9500
C(36)-C(35)	1.393(7)
C(36)-C(37)	1.396(8)
C(36)-C(38)	1.537(8)
C(37)-H(37A)	0.9500
C(57)-C(54)	1.399(8)
C(57)-C(70)	1.531(8)
C(56)-H(56A)	0.9500
C(19)-C(21)	1.516(8)
C(19)-C(22)	1.540(8)
C(19)-C(20)	1.546(9)
C(45)-C(44)	1.394(7)
C(45)-H(45A)	0.9500
C(48)-C(51)	1.473(9)
C(48)-C(50)	1.492(10)
C(48)-C(49)	1.504(11)
C(54)-H(54A)	0.9500
C(44)-H(44A)	0.9500
C(18)-H(18A)	0.9500
C(23)-C(25)	1.524(9)
C(23)-C(24)	1.523(8)
C(23)-C(26)	1.524(9)
C(35)-H(35A)	0.9500
C(38)-C(41)	1.516(8)
C(38)-C(40)	1.524(9)
C(38)-C(39)	1.542(8)
C(70)-C(68)	1.500(9)
C(70)-C(69)	1.515(9)
C(70)-C(71)	1.527(8)
C(62)-C(63)	1.383(8)
C(62)-C(61)	1.402(8)
C(62)-C(64)	1.529(8)
C(63)-H(63A)	0.9500
C(60)-C(61)	1.386(8)
C(60)-H(60A)	0.9500

C(2)-C(3)	1.362(8)
C(2)-H(2A)	0.9500
C(61)-H(61A)	0.9500
C(3)-C(4)	1.356(9)
C(3)-H(3A)	0.9500
C(64)-C(66)	1.463(11)
C(64)-C(67)	1.492(11)
C(64)-C(65)	1.513(10)
C(24)-H(24A)	0.9800
C(24)-H(24B)	0.9800
C(24)-H(24C)	0.9800
C(21)-H(21A)	0.9800
C(21)-H(21B)	0.9800
C(21)-H(21C)	0.9800
C(68)-H(68A)	0.9800
C(68)-H(68B)	0.9800
C(68)-H(68C)	0.9800
C(22)-H(22A)	0.9800
C(22)-H(22B)	0.9800
C(22)-H(22C)	0.9800
C(25)-H(25A)	0.9800
C(25)-H(25B)	0.9800
C(25)-H(25C)	0.9800
C(4)-C(5)	1.352(9)
C(4)-H(4A)	0.9500
C(20)-H(20A)	0.9800
C(20)-H(20B)	0.9800
C(20)-H(20C)	0.9800
C(40)-H(40A)	0.9800
C(40)-H(40B)	0.9800
C(40)-H(40C)	0.9800
C(69)-H(69A)	0.9800
C(69)-H(69B)	0.9800
C(69)-H(69C)	0.9800
C(71)-H(71A)	0.9800
C(71)-H(71B)	0.9800
C(71)-H(71C)	0.9800
C(39)-H(39A)	0.9800
C(39)-H(39B)	0.9800
C(39)-H(39C)	0.9800
C(41)-H(41A)	0.9800
C(41)-H(41B)	0.9800
C(41)-H(41C)	0.9800
C(6)-C(5)	1.394(10)

C(6)-H(6A)	0.9500
C(26)-H(26A)	0.9800
C(26)-H(26B)	0.9800
C(26)-H(26C)	0.9800
C(5)-H(5A)	0.9500
C(65)-H(65A)	0.9800
C(65)-H(65B)	0.9800
C(65)-H(65C)	0.9800
C(51)-H(51A)	0.9800
C(51)-H(51B)	0.9800
C(51)-H(51C)	0.9800
C(50)-H(50A)	0.9800
C(50)-H(50B)	0.9800
C(50)-H(50C)	0.9800
C(49)-H(49A)	0.9800
C(49)-H(49B)	0.9800
C(49)-H(49C)	0.9800
C(67)-H(67A)	0.9800
C(67)-H(67B)	0.9800
C(67)-H(67C)	0.9800
C(66)-H(66A)	0.9800
C(66)-H(66B)	0.9800
C(66)-H(66C)	0.9800
Cl(2)-C(76)	1.680(17)
Cl(1)-C(76)	1.7101(10)
C(76)-H(76A)	0.9900
C(76)-H(76B)	0.9900

C(27)-N(3)-C(8)	121.5(4)
C(27)-N(3)-C(14)	128.6(4)
C(8)-N(3)-C(14)	106.9(4)
C(31)-N(1)-C(33)	119.8(4)
C(31)-N(1)-C(43)	127.3(4)
C(33)-N(1)-C(43)	106.4(4)
C(29)-N(4)-C(52)	126.5(4)
C(29)-N(4)-C(59)	125.5(4)
C(52)-N(4)-C(59)	107.8(4)
C(27)-N(2)-C(31)	116.7(4)
C(27)-N(2)-B(1)	122.4(4)
C(31)-N(2)-B(1)	120.9(4)
C(15)-C(14)-C(13)	119.7(5)
C(15)-C(14)-N(3)	131.8(5)
C(13)-C(14)-N(3)	108.2(4)
C(30)-C(31)-N(2)	123.0(5)

C(30)-C(31)-N(1)	120.4(4)
N(2)-C(31)-N(1)	116.6(4)
N(2)-C(27)-N(3)	117.8(4)
N(2)-C(27)-C(28)	122.2(4)
N(3)-C(27)-C(28)	119.9(4)
C(7)-C(8)-C(12)	125.2(5)
C(7)-C(8)-N(3)	124.6(4)
C(12)-C(8)-N(3)	110.1(4)
C(11)-C(12)-C(8)	118.6(5)
C(11)-C(12)-C(13)	134.5(5)
C(8)-C(12)-C(13)	106.9(4)
C(29)-C(28)-C(27)	119.9(5)
C(29)-C(28)-H(28A)	120.0
C(27)-C(28)-H(28A)	120.0
C(8)-C(7)-C(9)	113.3(5)
C(8)-C(7)-B(1)	117.8(5)
C(9)-C(7)-B(1)	128.6(5)
C(31)-C(30)-C(29)	119.2(5)
C(31)-C(30)-H(30A)	120.4
C(29)-C(30)-H(30A)	120.4
C(44)-C(43)-C(42)	120.3(5)
C(44)-C(43)-N(1)	131.1(5)
C(42)-C(43)-N(1)	108.4(4)
C(47)-C(42)-C(43)	120.2(5)
C(47)-C(42)-C(34)	131.3(5)
C(43)-C(42)-C(34)	108.5(4)
C(32)-C(33)-C(34)	125.4(5)
C(32)-C(33)-N(1)	123.6(5)
C(34)-C(33)-N(1)	111.0(4)
C(56)-C(52)-C(53)	120.7(5)
C(56)-C(52)-N(4)	130.5(5)
C(53)-C(52)-N(4)	108.8(5)
C(10)-C(11)-C(12)	119.3(5)
C(10)-C(11)-H(11A)	120.4
C(12)-C(11)-H(11A)	120.4
C(47)-C(46)-C(45)	117.2(5)
C(47)-C(46)-C(48)	120.7(5)
C(45)-C(46)-C(48)	122.1(5)
C(28)-C(29)-C(30)	119.0(5)
C(28)-C(29)-N(4)	120.4(5)
C(30)-C(29)-N(4)	120.5(4)
C(15)-C(16)-C(17)	123.8(5)
C(15)-C(16)-H(16A)	118.1
C(17)-C(16)-H(16A)	118.1

C(7)-C(9)-C(10)	124.7(5)
C(7)-C(9)-H(9A)	117.6
C(10)-C(9)-H(9A)	117.6
C(35)-C(34)-C(33)	118.4(5)
C(35)-C(34)-C(42)	136.1(5)
C(33)-C(34)-C(42)	105.5(5)
C(11)-C(10)-C(9)	118.7(5)
C(11)-C(10)-C(23)	122.2(5)
C(9)-C(10)-C(23)	119.1(5)
C(60)-C(59)-C(58)	120.7(5)
C(60)-C(59)-N(4)	130.5(5)
C(58)-C(59)-N(4)	108.6(4)
C(18)-C(17)-C(16)	117.7(5)
C(18)-C(17)-C(19)	123.1(5)
C(16)-C(17)-C(19)	119.2(5)
C(16)-C(15)-C(14)	118.0(5)
C(16)-C(15)-H(15A)	121.0
C(14)-C(15)-H(15A)	121.0
C(18)-C(13)-C(14)	120.5(5)
C(18)-C(13)-C(12)	131.6(5)
C(14)-C(13)-C(12)	107.8(4)
C(33)-C(32)-C(37)	113.6(5)
C(33)-C(32)-B(1)	115.7(4)
C(37)-C(32)-B(1)	130.4(5)
C(6)-C(1)-C(2)	114.5(5)
C(6)-C(1)-B(1)	120.6(5)
C(2)-C(1)-B(1)	124.9(5)
C(52)-C(53)-C(54)	120.2(5)
C(52)-C(53)-C(58)	107.4(5)
C(54)-C(53)-C(58)	132.4(5)
C(63)-C(58)-C(59)	119.9(5)
C(63)-C(58)-C(53)	132.7(5)
C(59)-C(58)-C(53)	107.4(5)
C(42)-C(47)-C(46)	121.0(5)
C(42)-C(47)-H(47A)	119.5
C(46)-C(47)-H(47A)	119.5
C(56)-C(55)-C(57)	123.1(5)
C(56)-C(55)-H(55A)	118.4
C(57)-C(55)-H(55A)	118.4
C(35)-C(36)-C(37)	119.2(5)
C(35)-C(36)-C(38)	122.6(5)
C(37)-C(36)-C(38)	118.2(5)
C(36)-C(37)-C(32)	123.4(5)
C(36)-C(37)-H(37A)	118.3

C(32)-C(37)-H(37A)	118.3
C(55)-C(57)-C(54)	117.4(5)
C(55)-C(57)-C(70)	120.0(5)
C(54)-C(57)-C(70)	122.6(5)
C(52)-C(56)-C(55)	118.1(5)
C(52)-C(56)-H(56A)	121.0
C(55)-C(56)-H(56A)	121.0
C(21)-C(19)-C(17)	109.4(5)
C(21)-C(19)-C(22)	108.8(5)
C(17)-C(19)-C(22)	111.6(5)
C(21)-C(19)-C(20)	109.9(5)
C(17)-C(19)-C(20)	108.7(5)
C(22)-C(19)-C(20)	108.4(5)
C(44)-C(45)-C(46)	122.8(5)
C(44)-C(45)-H(45A)	118.6
C(46)-C(45)-H(45A)	118.6
C(51)-C(48)-C(50)	115.1(7)
C(51)-C(48)-C(49)	104.5(7)
C(50)-C(48)-C(49)	105.3(8)
C(51)-C(48)-C(46)	112.9(5)
C(50)-C(48)-C(46)	109.3(5)
C(49)-C(48)-C(46)	109.2(5)
C(53)-C(54)-C(57)	120.5(5)
C(53)-C(54)-H(54A)	119.7
C(57)-C(54)-H(54A)	119.7
C(43)-C(44)-C(45)	118.5(5)
C(43)-C(44)-H(44A)	120.7
C(45)-C(44)-H(44A)	120.7
C(17)-C(18)-C(13)	120.4(5)
C(17)-C(18)-H(18A)	119.8
C(13)-C(18)-H(18A)	119.8
C(25)-C(23)-C(24)	108.4(5)
C(25)-C(23)-C(26)	109.3(6)
C(24)-C(23)-C(26)	108.0(5)
C(25)-C(23)-C(10)	109.2(5)
C(24)-C(23)-C(10)	113.5(5)
C(26)-C(23)-C(10)	108.3(5)
C(34)-C(35)-C(36)	119.7(5)
C(34)-C(35)-H(35A)	120.1
C(36)-C(35)-H(35A)	120.1
C(41)-C(38)-C(40)	109.8(6)
C(41)-C(38)-C(36)	112.9(5)
C(40)-C(38)-C(36)	110.0(5)
C(41)-C(38)-C(39)	107.7(6)

C(40)-C(38)-C(39)	108.9(5)
C(36)-C(38)-C(39)	107.4(5)
C(68)-C(70)-C(69)	108.1(6)
C(68)-C(70)-C(57)	111.1(5)
C(69)-C(70)-C(57)	109.8(5)
C(68)-C(70)-C(71)	108.2(5)
C(69)-C(70)-C(71)	108.1(5)
C(57)-C(70)-C(71)	111.4(5)
C(63)-C(62)-C(61)	117.0(5)
C(63)-C(62)-C(64)	121.2(5)
C(61)-C(62)-C(64)	121.8(6)
C(62)-C(63)-C(58)	121.3(5)
C(62)-C(63)-H(63A)	119.4
C(58)-C(63)-H(63A)	119.4
C(59)-C(60)-C(61)	117.8(6)
C(59)-C(60)-H(60A)	121.1
C(61)-C(60)-H(60A)	121.1
C(3)-C(2)-C(1)	123.3(6)
C(3)-C(2)-H(2A)	118.3
C(1)-C(2)-H(2A)	118.3
C(60)-C(61)-C(62)	123.2(6)
C(60)-C(61)-H(61A)	118.4
C(62)-C(61)-H(61A)	118.4
C(4)-C(3)-C(2)	120.7(6)
C(4)-C(3)-H(3A)	119.7
C(2)-C(3)-H(3A)	119.7
C(66)-C(64)-C(67)	110.5(9)
C(66)-C(64)-C(65)	116.7(8)
C(67)-C(64)-C(65)	97.9(7)
C(66)-C(64)-C(62)	109.9(6)
C(67)-C(64)-C(62)	110.8(6)
C(65)-C(64)-C(62)	110.5(6)
C(23)-C(24)-H(24A)	109.5
C(23)-C(24)-H(24B)	109.5
H(24A)-C(24)-H(24B)	109.5
C(23)-C(24)-H(24C)	109.5
H(24A)-C(24)-H(24C)	109.5
H(24B)-C(24)-H(24C)	109.5
C(19)-C(21)-H(21A)	109.5
C(19)-C(21)-H(21B)	109.5
H(21A)-C(21)-H(21B)	109.5
C(19)-C(21)-H(21C)	109.5
H(21A)-C(21)-H(21C)	109.5
H(21B)-C(21)-H(21C)	109.5

C(70)-C(68)-H(68A)	109.5
C(70)-C(68)-H(68B)	109.5
H(68A)-C(68)-H(68B)	109.5
C(70)-C(68)-H(68C)	109.5
H(68A)-C(68)-H(68C)	109.5
H(68B)-C(68)-H(68C)	109.5
C(19)-C(22)-H(22A)	109.5
C(19)-C(22)-H(22B)	109.5
H(22A)-C(22)-H(22B)	109.5
C(19)-C(22)-H(22C)	109.5
H(22A)-C(22)-H(22C)	109.5
H(22B)-C(22)-H(22C)	109.5
C(23)-C(25)-H(25A)	109.5
C(23)-C(25)-H(25B)	109.5
H(25A)-C(25)-H(25B)	109.5
C(23)-C(25)-H(25C)	109.5
H(25A)-C(25)-H(25C)	109.5
H(25B)-C(25)-H(25C)	109.5
C(5)-C(4)-C(3)	118.5(6)
C(5)-C(4)-H(4A)	120.7
C(3)-C(4)-H(4A)	120.7
C(19)-C(20)-H(20A)	109.5
C(19)-C(20)-H(20B)	109.5
H(20A)-C(20)-H(20B)	109.5
C(19)-C(20)-H(20C)	109.5
H(20A)-C(20)-H(20C)	109.5
H(20B)-C(20)-H(20C)	109.5
C(7)-B(1)-C(32)	115.4(5)
C(7)-B(1)-C(1)	112.0(5)
C(32)-B(1)-C(1)	112.8(4)
C(7)-B(1)-N(2)	106.2(4)
C(32)-B(1)-N(2)	103.6(4)
C(1)-B(1)-N(2)	105.7(4)
C(38)-C(40)-H(40A)	109.5
C(38)-C(40)-H(40B)	109.5
H(40A)-C(40)-H(40B)	109.5
C(38)-C(40)-H(40C)	109.5
H(40A)-C(40)-H(40C)	109.5
H(40B)-C(40)-H(40C)	109.5
C(70)-C(69)-H(69A)	109.5
C(70)-C(69)-H(69B)	109.5
H(69A)-C(69)-H(69B)	109.5
C(70)-C(69)-H(69C)	109.5
H(69A)-C(69)-H(69C)	109.5

H(69B)-C(69)-H(69C)	109.5
C(70)-C(71)-H(71A)	109.5
C(70)-C(71)-H(71B)	109.5
H(71A)-C(71)-H(71B)	109.5
C(70)-C(71)-H(71C)	109.5
H(71A)-C(71)-H(71C)	109.5
H(71B)-C(71)-H(71C)	109.5
C(38)-C(39)-H(39A)	109.5
C(38)-C(39)-H(39B)	109.5
H(39A)-C(39)-H(39B)	109.5
C(38)-C(39)-H(39C)	109.5
H(39A)-C(39)-H(39C)	109.5
H(39B)-C(39)-H(39C)	109.5
C(38)-C(41)-H(41A)	109.5
C(38)-C(41)-H(41B)	109.5
H(41A)-C(41)-H(41B)	109.5
C(38)-C(41)-H(41C)	109.5
H(41A)-C(41)-H(41C)	109.5
H(41B)-C(41)-H(41C)	109.5
C(1)-C(6)-C(5)	122.6(6)
C(1)-C(6)-H(6A)	118.7
C(5)-C(6)-H(6A)	118.7
C(23)-C(26)-H(26A)	109.5
C(23)-C(26)-H(26B)	109.5
H(26A)-C(26)-H(26B)	109.5
C(23)-C(26)-H(26C)	109.5
H(26A)-C(26)-H(26C)	109.5
H(26B)-C(26)-H(26C)	109.5
C(4)-C(5)-C(6)	120.3(7)
C(4)-C(5)-H(5A)	119.9
C(6)-C(5)-H(5A)	119.9
C(64)-C(65)-H(65A)	109.5
C(64)-C(65)-H(65B)	109.5
H(65A)-C(65)-H(65B)	109.5
C(64)-C(65)-H(65C)	109.5
H(65A)-C(65)-H(65C)	109.5
H(65B)-C(65)-H(65C)	109.5
C(48)-C(51)-H(51A)	109.5
C(48)-C(51)-H(51B)	109.5
H(51A)-C(51)-H(51B)	109.5
C(48)-C(51)-H(51C)	109.5
H(51A)-C(51)-H(51C)	109.5
H(51B)-C(51)-H(51C)	109.5
C(48)-C(50)-H(50A)	109.5

C(48)-C(50)-H(50B)	109.5
H(50A)-C(50)-H(50B)	109.5
C(48)-C(50)-H(50C)	109.5
H(50A)-C(50)-H(50C)	109.5
H(50B)-C(50)-H(50C)	109.5
C(48)-C(49)-H(49A)	109.5
C(48)-C(49)-H(49B)	109.5
H(49A)-C(49)-H(49B)	109.5
C(48)-C(49)-H(49C)	109.5
H(49A)-C(49)-H(49C)	109.5
H(49B)-C(49)-H(49C)	109.5
C(64)-C(67)-H(67A)	109.5
C(64)-C(67)-H(67B)	109.5
H(67A)-C(67)-H(67B)	109.5
C(64)-C(67)-H(67C)	109.5
H(67A)-C(67)-H(67C)	109.5
H(67B)-C(67)-H(67C)	109.5
C(64)-C(66)-H(66A)	109.5
C(64)-C(66)-H(66B)	109.5
H(66A)-C(66)-H(66B)	109.5
C(64)-C(66)-H(66C)	109.5
H(66A)-C(66)-H(66C)	109.5
H(66B)-C(66)-H(66C)	109.5
Cl(2)-C(76)-Cl(1)	112.8(11)
Cl(2)-C(76)-H(76A)	109.0
Cl(1)-C(76)-H(76A)	109.0
Cl(2)-C(76)-H(76B)	109.0
Cl(1)-C(76)-H(76B)	109.0
H(76A)-C(76)-H(76B)	107.8

Symmetry transformations used to generate equivalent atoms: #1 -x+1, -y+1, -z+1

Table S7.9 Anisotropic displacement parameters (Å^2 x 10^3) for **TCz-BN1**; the anisotropic displacement factor exponent takes the form: $-2\pi^2$ [h^2 $a^{*2}U^{11}$ + ... + 2hka* b* U^{12}]

	U11	U22	U33	U23	U13	U12
N(3)	24(2)	37(3)	24(2)	2(2)	6(2)	-4(2)
N(1)	27(2)	36(3)	28(3)	5(2)	10(2)	-5(2)

N(4)	38(3)	39(3)	25(3)	0(2)	11(2)	-11(2)
N(2)	24(2)	34(3)	23(2)	0(2)	7(2)	-2(2)
C(14)	24(3)	31(3)	32(3)	0(2)	10(2)	-2(2)
C(31)	23(3)	31(3)	27(3)	1(2)	5(2)	-1(2)
C(27)	23(3)	28(3)	26(3)	0(2)	8(2)	-1(2)
C(8)	28(3)	26(3)	29(3)	3(2)	8(2)	4(2)
C(12)	25(3)	32(3)	30(3)	0(2)	10(2)	1(2)
C(28)	29(3)	33(3)	30(3)	-1(2)	12(2)	-4(2)
C(7)	31(3)	29(3)	26(3)	-4(2)	5(2)	-3(2)
C(30)	28(3)	37(3)	29(3)	-1(2)	6(2)	-5(2)
C(43)	29(3)	32(3)	34(3)	-2(2)	15(2)	-4(2)
C(42)	27(3)	39(3)	28(3)	0(2)	12(2)	2(2)
C(33)	27(3)	34(3)	29(3)	3(2)	7(2)	1(2)
C(52)	30(3)	42(4)	23(3)	-1(3)	9(2)	-4(2)
C(11)	25(3)	35(3)	34(3)	0(2)	9(2)	-5(2)
C(46)	29(3)	35(3)	39(3)	2(3)	12(3)	-2(2)
C(29)	32(3)	32(3)	22(3)	0(2)	6(2)	-5(2)
C(16)	39(3)	38(3)	28(3)	7(3)	12(3)	0(3)
C(9)	32(3)	44(3)	22(3)	0(2)	11(2)	-6(3)
C(34)	28(3)	39(3)	30(3)	-2(3)	8(2)	-4(2)
C(10)	28(3)	35(3)	29(3)	-5(2)	7(2)	-6(2)
C(59)	30(3)	37(3)	31(3)	-4(3)	9(2)	-13(2)
C(17)	30(3)	42(3)	30(3)	5(3)	11(2)	3(3)
C(15)	30(3)	36(3)	27(3)	6(2)	-1(2)	-1(2)
C(13)	27(3)	38(3)	32(3)	2(2)	13(2)	2(2)
C(32)	25(3)	39(3)	29(3)	-1(2)	10(2)	-1(2)
C(1)	32(3)	41(3)	23(3)	-2(2)	6(2)	-1(3)
C(53)	33(3)	39(3)	27(3)	-3(3)	7(2)	-5(2)
C(58)	36(3)	35(3)	28(3)	1(3)	8(2)	-4(2)
C(47)	34(3)	34(3)	38(3)	6(3)	17(3)	-2(2)
C(55)	37(3)	41(3)	27(3)	2(3)	7(2)	-6(3)
C(36)	34(3)	42(3)	33(3)	2(3)	10(3)	-1(3)
C(37)	29(3)	42(3)	31(3)	3(3)	8(2)	-4(2)
C(57)	34(3)	42(4)	29(3)	0(3)	5(2)	1(3)
C(56)	36(3)	38(4)	34(3)	-5(3)	10(3)	-2(3)
C(19)	27(3)	56(4)	43(4)	12(3)	16(3)	-1(3)
C(45)	30(3)	41(3)	39(4)	-1(3)	10(3)	-9(3)
C(48)	40(1)	40(1)	40(1)	0(1)	10(1)	0(1)
C(54)	39(3)	45(4)	26(3)	-8(3)	9(2)	-8(3)
C(44)	33(3)	48(4)	25(3)	-2(3)	9(2)	-7(3)
C(18)	25(3)	36(3)	32(3)	4(3)	6(2)	0(2)
C(23)	30(3)	51(4)	35(3)	-1(3)	5(3)	-7(3)
C(35)	31(3)	48(4)	31(3)	4(3)	16(3)	-2(3)
C(38)	33(3)	56(4)	34(3)	9(3)	2(3)	-8(3)

C(70)	44(3)	54(4)	28(3)	6(3)	11(3)	-5(3)
C(62)	53(4)	33(3)	42(4)	-8(3)	7(3)	-4(3)
C(63)	45(4)	45(4)	34(3)	-5(3)	3(3)	-6(3)
C(60)	49(4)	45(4)	34(3)	-2(3)	6(3)	-13(3)
C(2)	33(3)	44(4)	47(4)	6(3)	9(3)	1(3)
C(61)	58(4)	38(4)	45(4)	4(3)	9(3)	-9(3)
C(3)	42(4)	53(4)	51(4)	1(3)	17(3)	8(3)
C(64)	51(1)	51(1)	51(1)	0(1)	13(1)	0(1)
C(24)	37(4)	97(6)	33(4)	2(3)	1(3)	-11(3)
C(21)	41(4)	63(5)	63(5)	29(4)	21(3)	-3(3)
C(68)	81(5)	57(4)	38(4)	6(3)	19(3)	-3(4)
C(22)	29(3)	88(5)	49(4)	18(4)	14(3)	-13(3)
C(25)	49(4)	100(6)	47(4)	-34(4)	1(3)	1(4)
C(4)	56(4)	43(4)	76(5)	-6(4)	23(4)	11(3)
C(20)	47(4)	92(6)	56(5)	-8(4)	28(3)	4(4)
B(1)	26(3)	43(4)	22(3)	-4(3)	5(3)	-5(3)
C(40)	81(5)	77(5)	33(4)	2(4)	10(3)	1(4)
C(69)	66(5)	103(6)	39(4)	7(4)	20(3)	-23(4)
C(71)	77(5)	75(5)	28(4)	0(3)	20(3)	-7(4)
C(39)	49(4)	64(5)	55(4)	12(3)	8(3)	10(3)
C(41)	59(4)	91(6)	53(5)	37(4)	0(4)	-17(4)
C(6)	60(4)	46(4)	117(7)	-1(4)	55(4)	3(4)
C(26)	58(4)	81(5)	53(5)	23(4)	-13(3)	-16(4)
C(5)	82(6)	48(5)	162(9)	1(5)	81(6)	-5(4)
C(65)	161(9)	47(5)	91(7)	0(5)	-1(6)	-21(5)
C(51)	27(4)	166(10)	152(9)	106(8)	3(5)	-15(5)
C(50)	45(5)	67(6)	275(15)	41(7)	44(7)	-11(4)
C(49)	90(7)	202(12)	111(8)	-29(8)	55(6)	-81(8)
C(67)	293(16)	65(6)	86(7)	-40(5)	82(9)	-66(8)
C(66)	68(6)	123(9)	306(14)	-127(9)	16(8)	-18(6)
Cl(2)	274(8)	443(14)	386(11)	-68(10)	14(8)	-118(9)
Cl(1)	332(3)	332(3)	332(3)	0(1)	83(1)	0(1)
C(76)	238(11)	232(12)	202(11)	13(9)	80(9)	-25(9)

Table S7.10 Hydrogen coordinates (x 10^4) and isotropic displacement parameters (Å^2 x 10^3) for **TCz-BN1**.

	x	y	z	U(eq)

H(28A)	6021	6453	1483	36
H(30A)	2763	5951	2004	38
H(11A)	11166	6886	4158	37
H(16A)	8773	7175	831	41
H(9A)	8144	6656	5095	39
H(15A)	6988	6961	1270	39
H(47A)	954	5397	4384	41
H(55A)	4565	7028	-613	42
H(37A)	6814	6202	5285	41
H(56A)	4611	6827	553	43
H(45A)	-915	5768	2387	44
H(54A)	3255	5824	-1330	44
H(44A)	1026	6116	2256	42
H(18A)	11311	7092	2772	37
H(35A)	3516	5498	5325	43
H(63A)	2530	5069	-763	51
H(60A)	3414	5264	1710	52
H(2A)	3477	6910	4155	49
H(61A)	2575	4602	1185	57
H(3A)	2536	7584	4192	57
H(24A)	12512	6620	5171	86
H(24B)	12285	7129	5302	86
H(24C)	12780	6806	5966	86
H(21A)	11690	7848	836	81
H(21B)	10136	7726	686	81
H(21C)	10870	7967	1411	81
H(68A)	2224	6910	-2091	87
H(68B)	3454	7227	-1755	87
H(68C)	3208	7066	-2563	87
H(22A)	12937	7069	2197	82
H(22B)	13383	7444	1728	82
H(22C)	12615	7571	2323	82
H(25A)	9399	7014	5971	101
H(25B)	10904	7044	6448	101
H(25C)	10408	7367	5784	101
H(4A)	3622	8211	3971	68
H(20A)	11731	6683	1080	94
H(20B)	10649	6941	483	94
H(20C)	12203	7060	624	94
H(40A)	6937	6246	6450	96
H(40B)	5614	6168	6722	96
H(40C)	6978	5926	7109	96
H(69A)	6041	6463	-1677	102

H(69B)	5494	6800	-2316	102
H(69C)	5739	6961	-1508	102
H(71A)	4143	5965	-2235	88
H(71B)	2646	6151	-2416	88
H(71C)	3675	6322	-2851	88
H(39A)	7877	5631	5859	85
H(39B)	7871	5321	6525	85
H(39C)	7117	5170	5736	85
H(41A)	5031	5063	6117	105
H(41B)	5816	5205	6906	105
H(41C)	4452	5446	6519	105
H(6A)	6707	7465	3758	82
H(26A)	10983	6064	5470	103
H(26B)	11301	6261	6260	103
H(26C)	9784	6215	5804	103
H(5A)	5730	8150	3771	106
H(65A)	2196	3914	672	158
H(65B)	3485	3999	372	158
H(65C)	2319	3682	-49	158
H(51A)	-2524	5318	2438	178
H(51B)	-2768	5733	2896	178
H(51C)	-3429	5270	2995	178
H(50A)	-1884	4671	3700	192
H(50B)	-313	4771	3901	192
H(50C)	-1096	4695	3087	192
H(49A)	-1597	5749	4222	193
H(49B)	-1225	5269	4544	193
H(49C)	-2725	5377	4112	193
H(67A)	1998	3944	-1011	212
H(67B)	3197	4288	-778	212
H(67C)	1760	4449	-1231	212
H(66A)	17	4590	-653	257
H(66B)	113	4401	128	257
H(66C)	-15	4072	-529	257
H(76A)	-516	3987	1357	263
H(76B)	1083	3993	1611	263

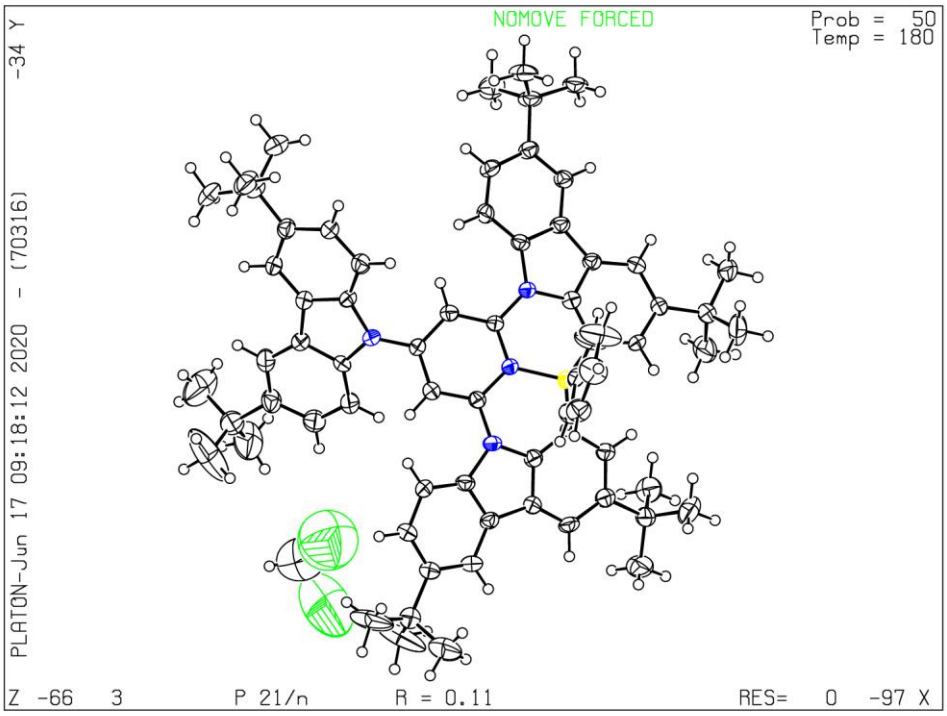

The ORTEP diagram showing the structure of **TCz-BN1** with labeling schemes

Table S7.11 Atomic coordinates (x 10^4) and equivalent isotropic displacement parameters ($Å^2$ x 10^3) for **BN2**. U(eq) is defined as one third of the trace of the orthogonalized U^{ij} tensor.

	x	y	z	U(eq)
N(1)	4726(2)	706(1)	3739(1)	35(1)
N(3)	3323(2)	608(1)	3100(2)	35(1)
N(4)	3712(2)	1316(1)	3603(1)	35(1)
C(1)	1704(5)	3003(2)	4076(5)	145(3)
C(2)	1239(3)	2594(1)	3868(2)	58(1)
C(3)	1879(3)	2253(1)	3815(2)	48(1)
C(4)	1598(3)	1843(1)	3835(2)	45(1)
C(5)	2150(2)	1516(1)	3767(2)	40(1)
C(6)	3036(2)	1591(1)	3681(2)	37(1)
C(7)	2590(2)	640(1)	2646(2)	36(1)
C(8)	2248(2)	986(1)	2313(2)	39(1)
C(9)	1564(2)	934(1)	1851(2)	43(1)
C(10)	1174(2)	555(1)	1700(2)	42(1)

C(11)	779(3)	615(1)	375(2)	67(1)
C(12)	2554(4)	-1172(2)	4101(3)	99(2)
C(13)	3376(3)	-1091(1)	3613(2)	54(1)
C(14)	4128(4)	-1240(1)	4078(3)	83(2)
C(15)	3422(3)	-628(1)	3448(2)	44(1)
C(16)	2783(2)	-453(1)	3018(2)	40(1)
C(17)	2772(2)	-36(1)	2886(2)	38(1)
C(18)	2244(2)	257(1)	2502(2)	38(1)
C(19)	1536(2)	219(1)	2030(2)	42(1)
C(20)	-277(3)	848(1)	1341(2)	70(1)
C(21)	-7(3)	119(1)	1169(2)	64(1)
C(22)	4839(2)	294(1)	3803(2)	38(1)
C(23)	4104(2)	31(1)	3619(2)	38(1)
C(24)	4078(2)	-388(1)	3731(2)	41(1)
C(25)	3433(2)	196(1)	3219(2)	35(1)
C(26)	5646(2)	139(1)	3970(2)	47(1)
C(27)	6333(3)	395(1)	4044(2)	50(1)
C(28)	6229(2)	799(1)	3937(2)	45(1)
C(29)	5428(2)	963(1)	3761(2)	38(1)
C(30)	5295(2)	1391(1)	3651(2)	37(1)
C(31)	5963(2)	1681(1)	3645(2)	41(1)
C(32)	5808(3)	2098(1)	3610(2)	47(1)
C(33)	4957(3)	2236(1)	3602(2)	44(1)
C(34)	4264(2)	1962(1)	3591(2)	38(1)
C(35)	4462(2)	1542(1)	3586(2)	35(1)
C(36)	625(5)	2543(2)	4515(3)	123(2)
C(37)	827(4)	2658(2)	3163(3)	104(1)
C(38)	2767(3)	2316(1)	3729(2)	43(1)
C(39)	3338(2)	1996(1)	3654(2)	38(1)
C(40)	6540(3)	2412(1)	3642(2)	51(1)
C(41)	6497(3)	2622(2)	4404(2)	79(2)
C(42)	6412(3)	2735(1)	3040(3)	73(1)
C(43)	7426(3)	2223(1)	3541(3)	80(2)
C(44)	3662(2)	931(1)	5156(2)	42(1)
C(45)	3317(3)	870(1)	5852(2)	49(1)
C(46)	2595(3)	636(1)	5943(2)	57(1)
C(47)	2219(3)	467(1)	5346(2)	56(1)
C(48)	2566(2)	525(1)	4640(2)	43(1)
C(49)	3277(5)	-1320(1)	2921(3)	95(2)
C(99)	3295(2)	758(1)	4530(2)	32(1)
C(100)	432(2)	533(1)	1153(2)	52(1)
B(5)	3713(2)	860(1)	3721(2)	33(1)

Table S7.12 Bond lengths [Å] and angles [°] for **BN2**.

N(1)-C(22)	1.376(4)
N(1)-C(29)	1.380(4)
N(1)-B(5)	1.650(5)
N(3)-C(25)	1.387(4)
N(3)-C(7)	1.437(5)
N(3)-B(5)	1.546(5)
N(4)-C(35)	1.379(4)
N(4)-C(6)	1.386(4)
N(4)-B(5)	1.518(4)
C(1)-C(2)	1.585(7)
C(1)-H(1A)	0.9800
C(1)-H(1B)	0.9800
C(1)-H(1C)	0.9800
C(2)-C(37)	1.468(7)
C(2)-C(36)	1.477(6)
C(2)-C(3)	1.497(5)
C(3)-C(38)	1.391(6)
C(3)-C(4)	1.420(5)
C(4)-C(5)	1.377(5)
C(4)-H(4)	0.9500
C(5)-C(6)	1.393(5)
C(5)-H(5)	0.9500
C(6)-C(39)	1.418(4)
C(7)-C(18)	1.401(4)
C(7)-C(8)	1.406(5)
C(8)-C(9)	1.393(5)
C(8)-H(8)	0.9500
C(9)-C(10)	1.419(5)
C(9)-H(9)	0.9500
C(10)-C(19)	1.390(5)
C(10)-C(100)	1.554(5)
C(11)-C(100)	1.503(6)
C(11)-H(11A)	0.9800
C(11)-H(11B)	0.9800
C(11)-H(11C)	0.9800
C(12)-C(13)	1.531(7)
C(12)-H(12A)	0.9800
C(12)-H(12B)	0.9800
C(12)-H(12C)	0.9800
C(13)-C(49)	1.476(6)

C(13)-C(14)	1.549(7)
C(13)-C(15)	1.558(5)
C(14)-H(14A)	0.9800
C(14)-H(14B)	0.9800
C(14)-H(14C)	0.9800
C(15)-C(24)	1.404(5)
C(15)-C(16)	1.413(5)
C(16)-C(17)	1.396(5)
C(16)-H(16)	0.9500
C(17)-C(25)	1.436(5)
C(17)-C(18)	1.465(5)
C(18)-C(19)	1.428(5)
C(19)-H(19)	0.9500
C(20)-C(100)	1.537(5)
C(20)-H(20A)	0.9800
C(20)-H(20B)	0.9800
C(20)-H(20C)	0.9800
C(21)-C(100)	1.525(6)
C(21)-H(21A)	0.9800
C(21)-H(21B)	0.9800
C(21)-H(21C)	0.9800
C(22)-C(26)	1.398(5)
C(22)-C(23)	1.482(5)
C(23)-C(24)	1.397(5)
C(23)-C(25)	1.405(5)
C(24)-H(24)	0.9500
C(26)-C(27)	1.371(5)
C(26)-H(26)	0.9500
C(27)-C(28)	1.360(5)
C(27)-H(27)	0.9500
C(28)-C(29)	1.404(5)
C(28)-H(28)	0.9500
C(29)-C(30)	1.441(5)
C(30)-C(35)	1.394(5)
C(30)-C(31)	1.409(5)
C(31)-C(32)	1.398(5)
C(31)-H(31)	0.9500
C(32)-C(33)	1.395(6)
C(32)-C(40)	1.538(5)
C(33)-C(34)	1.403(5)
C(33)-H(33)	0.9500
C(34)-C(35)	1.419(5)
C(34)-C(39)	1.435(5)
C(36)-H(36A)	0.9800

C(36)-H(36B)	0.9800
C(36)-H(36C)	0.9800
C(37)-H(37A)	0.9800
C(37)-H(37B)	0.9800
C(37)-H(37C)	0.9800
C(38)-C(39)	1.377(5)
C(38)-H(38)	0.9500
C(40)-C(43)	1.510(7)
C(40)-C(41)	1.538(5)
C(40)-C(42)	1.543(6)
C(41)-H(41A)	0.9800
C(41)-H(41B)	0.9800
C(41)-H(41C)	0.9800
C(42)-H(42A)	0.9800
C(42)-H(42B)	0.9800
C(42)-H(42C)	0.9800
C(43)-H(43A)	0.9800
C(43)-H(43B)	0.9800
C(43)-H(43C)	0.9800
C(44)-C(45)	1.356(5)
C(44)-C(99)	1.411(5)
C(44)-H(44)	0.9500
C(45)-C(46)	1.360(6)
C(45)-H(45)	0.9500
C(46)-C(47)	1.370(6)
C(46)-H(46)	0.9500
C(47)-C(48)	1.370(5)
C(47)-H(47)	0.9500
C(48)-C(99)	1.372(5)
C(48)-H(48)	0.9500
C(49)-H(49A)	0.9500
C(49)-H(49B)	0.9500
C(99)-B(5)	1.603(5)

C(22)-N(1)-C(29)	120.1(3)
C(22)-N(1)-B(5)	115.5(2)
C(29)-N(1)-B(5)	124.1(2)
C(25)-N(3)-C(7)	105.5(2)
C(25)-N(3)-B(5)	111.0(3)
C(7)-N(3)-B(5)	136.4(3)
C(35)-N(4)-C(6)	106.2(2)
C(35)-N(4)-B(5)	122.2(3)
C(6)-N(4)-B(5)	129.8(3)
C(2)-C(1)-H(1A)	109.5

C(2)-C(1)-H(1B)	109.5
H(1A)-C(1)-H(1B)	109.5
C(2)-C(1)-H(1C)	109.5
H(1A)-C(1)-H(1C)	109.5
H(1B)-C(1)-H(1C)	109.5
C(37)-C(2)-C(36)	114.4(5)
C(37)-C(2)-C(3)	111.7(4)
C(36)-C(2)-C(3)	111.1(4)
C(37)-C(2)-C(1)	107.8(5)
C(36)-C(2)-C(1)	101.0(5)
C(3)-C(2)-C(1)	110.3(4)
C(38)-C(3)-C(4)	116.3(3)
C(38)-C(3)-C(2)	122.9(3)
C(4)-C(3)-C(2)	120.8(4)
C(5)-C(4)-C(3)	123.8(4)
C(5)-C(4)-H(4)	118.1
C(3)-C(4)-H(4)	118.1
C(4)-C(5)-C(6)	118.3(3)
C(4)-C(5)-H(5)	120.9
C(6)-C(5)-H(5)	120.9
N(4)-C(6)-C(5)	129.0(3)
N(4)-C(6)-C(39)	111.5(3)
C(5)-C(6)-C(39)	119.4(3)
C(18)-C(7)-C(8)	119.7(3)
C(18)-C(7)-N(3)	111.1(3)
C(8)-C(7)-N(3)	129.0(3)
C(9)-C(8)-C(7)	118.1(3)
C(9)-C(8)-H(8)	120.9
C(7)-C(8)-H(8)	120.9
C(8)-C(9)-C(10)	124.6(3)
C(8)-C(9)-H(9)	117.7
C(10)-C(9)-H(9)	117.7
C(19)-C(10)-C(9)	115.7(3)
C(19)-C(10)-C(100)	124.2(3)
C(9)-C(10)-C(100)	119.9(3)
C(100)-C(11)-H(11A)	109.5
C(100)-C(11)-H(11B)	109.5
H(11A)-C(11)-H(11B)	109.5
C(100)-C(11)-H(11C)	109.5
H(11A)-C(11)-H(11C)	109.5
H(11B)-C(11)-H(11C)	109.5
C(13)-C(12)-H(12A)	109.5
C(13)-C(12)-H(12B)	109.5
H(12A)-C(12)-H(12B)	109.5

C(13)-C(12)-H(12C)	109.5
H(12A)-C(12)-H(12C)	109.5
H(12B)-C(12)-H(12C)	109.5
C(49)-C(13)-C(12)	106.2(4)
C(49)-C(13)-C(14)	114.3(4)
C(12)-C(13)-C(14)	105.0(4)
C(49)-C(13)-C(15)	110.2(3)
C(12)-C(13)-C(15)	107.9(3)
C(14)-C(13)-C(15)	112.7(3)
C(13)-C(14)-H(14A)	109.5
C(13)-C(14)-H(14B)	109.5
H(14A)-C(14)-H(14B)	109.5
C(13)-C(14)-H(14C)	109.5
H(14A)-C(14)-H(14C)	109.5
H(14B)-C(14)-H(14C)	109.5
C(24)-C(15)-C(16)	120.7(3)
C(24)-C(15)-C(13)	120.7(3)
C(16)-C(15)-C(13)	118.7(3)
C(17)-C(16)-C(15)	120.7(3)
C(17)-C(16)-H(16)	119.6
C(15)-C(16)-H(16)	119.6
C(16)-C(17)-C(25)	116.1(3)
C(16)-C(17)-C(18)	138.1(3)
C(25)-C(17)-C(18)	105.8(3)
C(7)-C(18)-C(19)	120.1(3)
C(7)-C(18)-C(17)	106.2(3)
C(19)-C(18)-C(17)	133.6(3)
C(10)-C(19)-C(18)	121.7(3)
C(10)-C(19)-H(19)	119.2
C(18)-C(19)-H(19)	119.2
C(100)-C(20)-H(20A)	109.5
C(100)-C(20)-H(20B)	109.5
H(20A)-C(20)-H(20B)	109.5
C(100)-C(20)-H(20C)	109.5
H(20A)-C(20)-H(20C)	109.5
H(20B)-C(20)-H(20C)	109.5
C(100)-C(21)-H(21A)	109.5
C(100)-C(21)-H(21B)	109.5
H(21A)-C(21)-H(21B)	109.5
C(100)-C(21)-H(21C)	109.5
H(21A)-C(21)-H(21C)	109.5
H(21B)-C(21)-H(21C)	109.5
N(1)-C(22)-C(26)	119.9(3)
N(1)-C(22)-C(23)	117.3(3)

C(26)-C(22)-C(23)	122.5(3)
C(24)-C(23)-C(25)	116.2(3)
C(24)-C(23)-C(22)	124.3(3)
C(25)-C(23)-C(22)	118.9(3)
C(23)-C(24)-C(15)	121.4(3)
C(23)-C(24)-H(24)	119.3
C(15)-C(24)-H(24)	119.3
N(3)-C(25)-C(23)	124.1(3)
N(3)-C(25)-C(17)	111.2(3)
C(23)-C(25)-C(17)	124.7(3)
C(27)-C(26)-C(22)	120.1(3)
C(27)-C(26)-H(26)	119.9
C(22)-C(26)-H(26)	119.9
C(28)-C(27)-C(26)	119.5(4)
C(28)-C(27)-H(27)	120.2
C(26)-C(27)-H(27)	120.2
C(27)-C(28)-C(29)	121.5(3)
C(27)-C(28)-H(28)	119.3
C(29)-C(28)-H(28)	119.3
N(1)-C(29)-C(28)	118.4(3)
N(1)-C(29)-C(30)	118.7(3)
C(28)-C(29)-C(30)	122.8(3)
C(35)-C(30)-C(31)	115.9(3)
C(35)-C(30)-C(29)	120.1(3)
C(31)-C(30)-C(29)	123.9(3)
C(32)-C(31)-C(30)	122.9(4)
C(32)-C(31)-H(31)	118.6
C(30)-C(31)-H(31)	118.6
C(33)-C(32)-C(31)	119.0(3)
C(33)-C(32)-C(40)	118.5(3)
C(31)-C(32)-C(40)	122.2(4)
C(32)-C(33)-C(34)	120.9(3)
C(32)-C(33)-H(33)	119.6
C(34)-C(33)-H(33)	119.6
C(33)-C(34)-C(35)	117.6(3)
C(33)-C(34)-C(39)	135.1(3)
C(35)-C(34)-C(39)	107.0(3)
N(4)-C(35)-C(30)	125.8(3)
N(4)-C(35)-C(34)	110.3(3)
C(30)-C(35)-C(34)	123.4(3)
C(2)-C(36)-H(36A)	109.5
C(2)-C(36)-H(36B)	109.5
H(36A)-C(36)-H(36B)	109.5
C(2)-C(36)-H(36C)	109.5

H(36A)-C(36)-H(36C)	109.5
H(36B)-C(36)-H(36C)	109.5
C(2)-C(37)-H(37A)	109.5
C(2)-C(37)-H(37B)	109.5
H(37A)-C(37)-H(37B)	109.5
C(2)-C(37)-H(37C)	109.5
H(37A)-C(37)-H(37C)	109.5
H(37B)-C(37)-H(37C)	109.5
C(39)-C(38)-C(3)	121.6(3)
C(39)-C(38)-H(38)	119.2
C(3)-C(38)-H(38)	119.2
C(38)-C(39)-C(6)	120.6(3)
C(38)-C(39)-C(34)	134.5(3)
C(6)-C(39)-C(34)	104.8(3)
C(43)-C(40)-C(41)	106.9(4)
C(43)-C(40)-C(32)	112.4(3)
C(41)-C(40)-C(32)	109.9(3)
C(43)-C(40)-C(42)	110.4(4)
C(41)-C(40)-C(42)	108.6(4)
C(32)-C(40)-C(42)	108.5(3)
C(40)-C(41)-H(41A)	109.5
C(40)-C(41)-H(41B)	109.5
H(41A)-C(41)-H(41B)	109.5
C(40)-C(41)-H(41C)	109.5
H(41A)-C(41)-H(41C)	109.5
H(41B)-C(41)-H(41C)	109.5
C(40)-C(42)-H(42A)	109.5
C(40)-C(42)-H(42B)	109.5
H(42A)-C(42)-H(42B)	109.5
C(40)-C(42)-H(42C)	109.5
H(42A)-C(42)-H(42C)	109.5
H(42B)-C(42)-H(42C)	109.5
C(40)-C(43)-H(43A)	109.5
C(40)-C(43)-H(43B)	109.5
H(43A)-C(43)-H(43B)	109.5
C(40)-C(43)-H(43C)	109.5
H(43A)-C(43)-H(43C)	109.5
H(43B)-C(43)-H(43C)	109.5
C(45)-C(44)-C(99)	121.8(3)
C(45)-C(44)-H(44)	119.1
C(99)-C(44)-H(44)	119.1
C(44)-C(45)-C(46)	118.5(4)
C(44)-C(45)-H(45)	120.7
C(46)-C(45)-H(45)	120.7

C(45)-C(46)-C(47)	121.1(3)
C(45)-C(46)-H(46)	119.4
C(47)-C(46)-H(46)	119.4
C(48)-C(47)-C(46)	120.9(4)
C(48)-C(47)-H(47)	119.6
C(46)-C(47)-H(47)	119.6
C(47)-C(48)-C(99)	119.4(3)
C(47)-C(48)-H(48)	120.3
C(99)-C(48)-H(48)	120.3
C(13)-C(49)-H(49A)	120.0
C(13)-C(49)-H(49B)	120.0
H(49A)-C(49)-H(49B)	120.0
C(48)-C(99)-C(44)	118.3(3)
C(48)-C(99)-B(5)	122.6(3)
C(44)-C(99)-B(5)	119.0(3)
C(11)-C(100)-C(21)	108.4(3)
C(11)-C(100)-C(20)	107.5(4)
C(21)-C(100)-C(20)	106.9(3)
C(11)-C(100)-C(10)	110.2(3)
C(21)-C(100)-C(10)	111.9(3)
C(20)-C(100)-C(10)	111.8(3)
N(4)-B(5)-N(3)	115.4(3)
N(4)-B(5)-C(99)	109.4(2)
N(3)-B(5)-C(99)	113.2(3)
N(4)-B(5)-N(1)	108.4(3)
N(3)-B(5)-N(1)	104.8(2)
C(99)-B(5)-N(1)	104.8(3)

Symmetry transformations used to generate equivalent atoms: #1 -x+1, -y+1, -z+1

Table S7.13 Anisotropic displacement parameters (Å^2 x 10^3) for **BN2**; the anisotropic displacement factor exponent takes the form: -2 π^2 [$h^2 a^{*2}U^{11}$ + ... + 2hka* b* U^{12}]

	U11	U22	U33	U23	U13	U12
N(1)	34(2)	32(1)	40(2)	-2(1)	1(1)	-5(1)
N(3)	33(2)	29(1)	43(2)	-4(1)	-4(1)	-2(1)
N(4)	34(2)	28(1)	42(2)	3(1)	-1(1)	-3(1)
C(1)	115(5)	73(4)	251(9)	-70(5)	-44(6)	32(4)

C(2)	59(1)	53(1)	62(1)	-2(1)	2(1)	5(1)
C(3)	59(3)	38(2)	44(2)	0(2)	9(2)	-1(1)
C(4)	41(2)	41(2)	51(2)	-3(2)	4(2)	-1(2)
C(5)	39(2)	30(2)	50(2)	3(1)	5(2)	-6(2)
C(6)	43(2)	31(2)	36(2)	1(1)	0(2)	1(2)
C(7)	37(2)	36(2)	33(2)	-3(1)	-1(2)	5(2)
C(8)	41(2)	39(2)	35(2)	-3(1)	3(2)	1(2)
C(9)	44(2)	48(2)	38(2)	1(2)	-4(2)	13(2)
C(10)	36(2)	50(2)	40(2)	-10(2)	1(2)	9(2)
C(11)	64(3)	88(3)	50(2)	-9(2)	-15(2)	9(2)
C(12)	99(4)	70(3)	125(5)	25(3)	15(4)	-6(3)
C(13)	66(3)	29(2)	68(2)	-4(2)	-12(2)	-10(2)
C(14)	105(4)	47(2)	100(4)	13(2)	-29(3)	-7(3)
C(15)	48(2)	32(2)	53(2)	-10(2)	-5(2)	1(2)
C(16)	39(2)	31(2)	50(2)	-9(1)	-5(2)	0(2)
C(17)	41(2)	32(2)	41(2)	-10(1)	-1(2)	-3(2)
C(18)	40(2)	39(2)	33(2)	-7(1)	-1(2)	-1(2)
C(19)	39(2)	49(2)	38(2)	-13(2)	-1(2)	0(2)
C(20)	56(3)	84(3)	71(3)	-23(2)	-22(2)	25(2)
C(21)	40(2)	78(3)	76(3)	-22(2)	-20(2)	6(2)
C(22)	38(2)	36(2)	41(2)	-3(1)	0(2)	4(2)
C(23)	41(2)	29(2)	44(2)	-6(1)	-2(2)	-1(2)
C(24)	41(2)	34(2)	49(2)	-2(2)	-6(2)	5(2)
C(25)	34(2)	30(2)	40(2)	-4(1)	-1(2)	-1(2)
C(26)	40(2)	40(2)	62(2)	-3(2)	-5(2)	4(2)
C(27)	34(2)	52(2)	65(2)	-3(2)	-4(2)	5(2)
C(28)	34(2)	49(2)	50(2)	0(2)	4(2)	-6(2)
C(29)	30(2)	48(2)	33(2)	-8(1)	7(1)	-9(2)
C(30)	42(2)	39(2)	31(2)	3(1)	2(2)	-7(2)
C(31)	41(2)	43(2)	40(2)	-1(1)	0(2)	-14(2)
C(32)	55(3)	46(2)	41(2)	-2(2)	7(2)	-17(2)
C(33)	53(3)	36(2)	42(2)	4(1)	0(2)	-10(2)
C(34)	50(2)	30(2)	35(2)	1(1)	1(2)	-12(2)
C(35)	40(2)	29(2)	34(2)	0(1)	1(2)	-10(2)
C(36)	130(5)	125(4)	109(2)	19(4)	62(3)	54(4)
C(37)	109(2)	114(2)	91(2)	2(2)	-28(2)	24(2)
C(38)	63(3)	26(2)	39(2)	3(1)	4(2)	1(2)
C(39)	46(2)	32(2)	35(2)	1(1)	0(2)	-5(2)
C(40)	53(3)	49(2)	50(2)	-3(2)	-2(2)	-28(2)
C(41)	77(3)	88(3)	71(3)	-21(2)	2(3)	-45(3)
C(42)	87(3)	55(3)	76(3)	5(2)	8(3)	-41(2)
C(43)	59(3)	74(3)	104(4)	3(3)	8(3)	-38(3)
C(44)	37(2)	44(2)	46(2)	-3(2)	0(2)	-7(2)
C(45)	62(3)	48(2)	36(2)	0(2)	-2(2)	-5(2)

C(46)	64(3)	57(2)	48(2)	7(2)	14(2)	1(2)
C(47)	54(3)	56(2)	57(3)	7(2)	13(2)	-14(2)
C(48)	37(2)	45(2)	46(2)	2(2)	-1(2)	-8(2)
C(49)	153(6)	40(2)	92(4)	-13(2)	-12(4)	-20(3)
C(99)	32(2)	24(2)	40(2)	4(1)	-5(1)	1(1)
C(100)	39(2)	70(3)	47(2)	-22(2)	-10(2)	14(2)
B(5)	27(2)	29(2)	41(2)	0(2)	1(2)	-1(2)

Table S7.14 Hydrogen coordinates (x 10^4) and isotropic displacement parameters (Å^2 x 10^3) for **BN2**.

	x	y	z	U(eq)
H(1A)	1267	3211	4199	218
H(1B)	2076	3094	3653	218
H(1C)	2055	2957	4503	218
H(4)	995	1792	3899	53
H(5)	1932	1247	3779	48
H(8)	2477	1247	2401	46
H(9)	1343	1168	1621	52
H(11A)	314	584	32	100
H(11B)	1245	423	243	100
H(11C)	1006	892	342	100
H(12A)	2046	-1102	3826	148
H(12B)	2562	-1005	4550	148
H(12C)	2529	-1459	4238	148
H(14A)	4681	-1190	3803	125
H(14B)	4063	-1531	4174	125
H(14C)	4116	-1093	4550	125
H(16)	2355	-621	2817	48
H(19)	1306	-42	1940	50
H(20A)	-727	829	981	105
H(20B)	-24	1121	1322	105
H(20C)	-532	796	1840	105
H(21A)	-504	125	855	97
H(21B)	-204	53	1679	97
H(21C)	406	-87	983	97
H(24)	4513	-513	4005	49

H(26)	5718	-145	4032	57
H(27)	6879	290	4169	60
H(28)	6709	975	3982	53
H(31)	6543	1590	3666	50
H(33)	4845	2519	3605	53
H(36A)	207	2330	4412	185
H(36B)	318	2798	4615	185
H(36C)	944	2467	4949	185
H(37A)	545	2406	3016	156
H(37B)	1267	2735	2779	156
H(37C)	394	2874	3223	156
H(38)	2985	2585	3722	51
H(41A)	6959	2825	4417	118
H(41B)	6572	2420	4793	118
H(41C)	5935	2756	4486	118
H(42A)	6889	2930	3037	110
H(42B)	5863	2877	3145	110
H(42C)	6403	2603	2554	110
H(43A)	7869	2434	3568	119
H(43B)	7483	2089	3056	119
H(43C)	7501	2022	3932	119
H(44)	4165	1095	5086	51
H(45)	3574	988	6267	58
H(46)	2348	590	6428	68
H(47)	1711	308	5423	67
H(48)	2303	405	4230	51
H(49A)	2769	-1476	2858	114
H(49B)	3720	-1312	2537	114

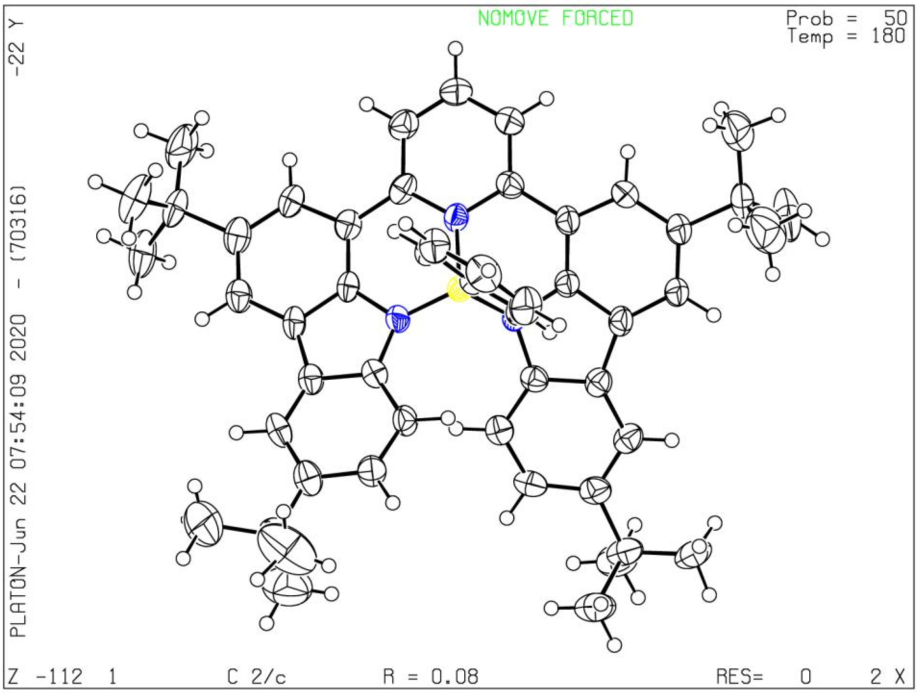

The ORTEP diagram showing the structure of **BN2** with labeling schemes

Table S7.15 Atomic coordinates (x 10^4) and equivalent isotropic displacement parameters ($Å^2$ x 10^3) for **BN3**. U(eq) is defined as one third of the trace of the orthogonalized U^{ij} tensor.

	x	y	z	U(eq)
C(1)	-2443(1)	8840(1)	1246(1)	20(1)
B(1)	-757(2)	8308(1)	63(1)	17(1)
N(2)	-1909(1)	9053(1)	346(1)	18(1)
C(2)	-1852(1)	8050(1)	1954(1)	20(1)
N(3)	-43(1)	7966(1)	854(1)	18(1)
C(3)	-667(1)	7702(1)	1724(1)	18(1)
N(4)	16(1)	9030(1)	-757(1)	18(1)
C(4)	1107(1)	7491(1)	900(1)	16(1)
C(5)	2093(1)	7521(1)	194(1)	16(1)
C(6)	2069(1)	8062(1)	-808(1)	17(1)
C(7)	1142(1)	8770(1)	-1187(1)	18(1)
C(8)	-579(1)	9707(1)	-1423(1)	20(1)

C(9)	-1774(1)	10077(1)	-1277(1)	20(1)
C(10)	-2409(1)	9827(1)	-331(1)	19(1)
C(11)	-1265(1)	7299(1)	-148(1)	19(1)
C(12)	-1107(2)	6262(1)	416(1)	30(1)
C(13)	-1485(2)	5378(2)	228(2)	39(1)
C(14)	-2032(2)	5505(2)	-531(2)	35(1)
C(15)	-2220(2)	6520(2)	-1094(2)	40(1)
C(16)	-1849(2)	7397(2)	-905(1)	35(1)
C(17)	-3472(1)	10372(1)	-107(1)	23(1)
C(18)	-4017(1)	10155(1)	794(1)	24(1)
C(19)	-3501(1)	9400(1)	1470(1)	22(1)
C(20)	-2351(2)	7658(1)	2875(1)	24(1)
C(21)	-1704(2)	6961(1)	3539(1)	24(1)
C(22)	-508(2)	6665(1)	3279(1)	23(1)
C(23)	29(1)	7043(1)	2366(1)	20(1)
C(24)	1202(1)	6927(1)	1835(1)	18(1)
C(25)	2294(1)	6440(1)	2088(1)	19(1)
C(26)	3305(1)	6514(1)	1424(1)	18(1)
C(27)	3168(1)	7040(1)	504(1)	18(1)
C(28)	3055(1)	7927(1)	-1457(1)	21(1)
C(29)	3210(1)	8489(1)	-2397(1)	22(1)
C(30)	2296(1)	9213(1)	-2735(1)	22(1)
C(31)	1257(1)	9333(1)	-2142(1)	19(1)
C(32)	127(1)	9935(1)	-2294(1)	20(1)
C(33)	-401(2)	10521(1)	-3056(1)	23(1)
C(34)	-1621(2)	10864(1)	-2951(1)	23(1)
C(35)	-2278(2)	10656(1)	-2064(1)	23(1)
C(36)	-2344(2)	6564(2)	4537(1)	33(1)
C(37)	-2641(2)	7523(2)	4914(2)	54(1)
C(38)	-3507(2)	6019(2)	4564(2)	54(1)
C(39)	-1550(2)	5759(2)	5163(1)	45(1)
C(40)	4564(1)	6070(1)	1652(1)	21(1)
C(41)	4538(2)	5492(2)	2678(1)	32(1)
C(42)	5436(2)	6989(2)	1391(2)	35(1)
C(43)	5053(2)	5278(2)	1114(1)	31(1)
C(44)	4338(2)	8262(1)	-3033(1)	28(1)
C(45)	4087(2)	7294(2)	-3328(2)	59(1)
C(46)	4606(2)	9200(2)	-3899(1)	34(1)
C(47)	5460(2)	8046(3)	-2558(2)	66(1)
C(48)	-2212(2)	11408(1)	-3815(1)	28(1)
C(49)	-1613(2)	12467(2)	-4349(1)	41(1)
C(50)	-2055(2)	10664(2)	-4430(2)	42(1)
C(51)	-3570(2)	11644(2)	-3589(1)	42(1)
Cl(4)	1015(1)	5724(1)	-2879(1)	110(1)

Cl(5)	-329(1)	7757(1)	-3346(1)	97(1)
C(52)	554(3)	6882(2)	-2578(2)	71(1)

Table S7.16 Bond lengths [Å] and angles [°] for **BN3**.

C(1)-N(2)	1.372(2)
C(1)-C(2)	1.465(2)
C(1)-C(19)	1.393(2)
B(1)-N(2)	1.636(2)
B(1)-N(3)	1.503(2)
B(1)-N(4)	1.517(2)
B(1)-C(11)	1.624(2)
N(2)-C(10)	1.376(2)
C(2)-C(3)	1.392(2)
C(2)-C(20)	1.393(2)
N(3)-C(3)	1.3642(19)
N(3)-C(4)	1.3878(19)
C(3)-C(23)	1.400(2)
N(4)-C(7)	1.3914(19)
N(4)-C(8)	1.367(2)
C(4)-C(5)	1.411(2)
C(4)-C(24)	1.425(2)
C(5)-C(6)	1.497(2)
C(5)-C(27)	1.400(2)
C(6)-C(7)	1.413(2)
C(6)-C(28)	1.401(2)
C(7)-C(31)	1.424(2)
C(8)-C(9)	1.391(2)
C(8)-C(32)	1.402(2)
C(9)-C(10)	1.467(2)
C(9)-C(35)	1.402(2)
C(10)-C(17)	1.386(2)
C(11)-C(12)	1.393(2)
C(11)-C(16)	1.398(2)
C(12)-C(13)	1.393(3)
C(13)-C(14)	1.371(3)
C(14)-C(15)	1.375(3)
C(15)-C(16)	1.384(3)
C(17)-C(18)	1.377(2)
C(18)-C(19)	1.372(2)
C(20)-C(21)	1.407(2)

C(21)-C(22)	1.393(2)
C(21)-C(36)	1.540(2)
C(22)-C(23)	1.393(2)
C(23)-C(24)	1.452(2)
C(24)-C(25)	1.391(2)
C(25)-C(26)	1.384(2)
C(26)-C(27)	1.406(2)
C(26)-C(40)	1.535(2)
C(28)-C(29)	1.403(2)
C(29)-C(30)	1.392(2)
C(29)-C(44)	1.534(2)
C(30)-C(31)	1.387(2)
C(31)-C(32)	1.450(2)
C(32)-C(33)	1.390(2)
C(33)-C(34)	1.402(2)
C(34)-C(35)	1.401(2)
C(34)-C(48)	1.537(2)
C(36)-C(37)	1.532(3)
C(36)-C(38)	1.528(3)
C(36)-C(39)	1.533(3)
C(40)-C(41)	1.531(2)
C(40)-C(42)	1.534(2)
C(40)-C(43)	1.532(2)
C(44)-C(45)	1.536(3)
C(44)-C(46)	1.526(2)
C(44)-C(47)	1.523(3)
C(48)-C(49)	1.533(3)
C(48)-C(50)	1.537(3)
C(48)-C(51)	1.531(3)
Cl(4)-C(52)	1.736(3)
Cl(5)-C(52)	1.754(3)
N(2)-C(1)-C(2)	118.64(13)
N(2)-C(1)-C(19)	119.74(15)
C(19)-C(1)-C(2)	121.57(14)
N(3)-B(1)-N(2)	107.17(12)
N(3)-B(1)-N(4)	108.28(13)
N(3)-B(1)-C(11)	112.34(13)
N(4)-B(1)-N(2)	106.14(12)
N(4)-B(1)-C(11)	113.90(13)
C(11)-B(1)-N(2)	108.63(12)
C(1)-N(2)-B(1)	119.92(12)
C(1)-N(2)-C(10)	120.12(13)
C(10)-N(2)-B(1)	119.69(12)

C(3)-C(2)-C(1)	118.95(14)
C(3)-C(2)-C(20)	115.77(15)
C(20)-C(2)-C(1)	125.13(14)
C(3)-N(3)-B(1)	118.05(12)
C(3)-N(3)-C(4)	107.60(12)
C(4)-N(3)-B(1)	129.83(13)
C(2)-C(3)-C(23)	123.72(14)
N(3)-C(3)-C(2)	124.26(14)
N(3)-C(3)-C(23)	112.02(13)
C(7)-N(4)-B(1)	126.83(13)
C(8)-N(4)-B(1)	116.83(12)
C(8)-N(4)-C(7)	107.21(13)
N(3)-C(4)-C(5)	129.88(14)
N(3)-C(4)-C(24)	108.76(13)
C(5)-C(4)-C(24)	121.35(14)
C(4)-C(5)-C(6)	125.22(14)
C(27)-C(5)-C(4)	114.41(14)
C(27)-C(5)-C(6)	120.29(13)
C(7)-C(6)-C(5)	125.76(14)
C(28)-C(6)-C(5)	120.01(14)
C(28)-C(6)-C(7)	114.15(14)
N(4)-C(7)-C(6)	129.31(14)
N(4)-C(7)-C(31)	109.00(13)
C(6)-C(7)-C(31)	121.68(14)
N(4)-C(8)-C(9)	124.55(14)
N(4)-C(8)-C(32)	112.10(13)
C(9)-C(8)-C(32)	123.22(15)
C(8)-C(9)-C(10)	118.47(14)
C(8)-C(9)-C(35)	116.27(15)
C(35)-C(9)-C(10)	125.26(14)
N(2)-C(10)-C(9)	118.56(13)
N(2)-C(10)-C(17)	120.00(14)
C(17)-C(10)-C(9)	121.42(15)
C(12)-C(11)-B(1)	120.99(15)
C(12)-C(11)-C(16)	115.64(16)
C(16)-C(11)-B(1)	123.34(15)
C(13)-C(12)-C(11)	122.22(18)
C(14)-C(13)-C(12)	120.44(18)
C(13)-C(14)-C(15)	118.85(18)
C(14)-C(15)-C(16)	120.61(19)
C(15)-C(16)-C(11)	122.22(18)
C(18)-C(17)-C(10)	120.09(15)
C(19)-C(18)-C(17)	119.79(15)
C(18)-C(19)-C(1)	120.23(15)

C(2)-C(20)-C(21)	122.58(15)
C(20)-C(21)-C(36)	118.50(14)
C(22)-C(21)-C(20)	119.32(15)
C(22)-C(21)-C(36)	122.18(15)
C(21)-C(22)-C(23)	119.96(15)
C(3)-C(23)-C(24)	104.79(13)
C(22)-C(23)-C(3)	118.54(14)
C(22)-C(23)-C(24)	136.65(15)
C(4)-C(24)-C(23)	106.78(13)
C(25)-C(24)-C(4)	120.83(14)
C(25)-C(24)-C(23)	132.33(15)
C(26)-C(25)-C(24)	119.54(15)
C(25)-C(26)-C(27)	118.11(14)
C(25)-C(26)-C(40)	122.94(14)
C(27)-C(26)-C(40)	118.94(13)
C(5)-C(27)-C(26)	125.53(14)
C(6)-C(28)-C(29)	125.43(15)
C(28)-C(29)-C(44)	120.03(14)
C(30)-C(29)-C(28)	118.38(14)
C(30)-C(29)-C(44)	121.53(14)
C(31)-C(30)-C(29)	119.18(15)
C(7)-C(31)-C(32)	106.78(13)
C(30)-C(31)-C(7)	120.94(14)
C(30)-C(31)-C(32)	132.23(15)
C(8)-C(32)-C(31)	104.87(13)
C(33)-C(32)-C(8)	118.77(14)
C(33)-C(32)-C(31)	135.81(15)
C(32)-C(33)-C(34)	120.18(15)
C(33)-C(34)-C(48)	118.88(15)
C(35)-C(34)-C(33)	119.04(15)
C(35)-C(34)-C(48)	122.02(15)
C(34)-C(35)-C(9)	122.38(15)
C(37)-C(36)-C(21)	108.64(16)
C(37)-C(36)-C(39)	108.07(18)
C(38)-C(36)-C(21)	109.85(16)
C(38)-C(36)-C(37)	110.04(18)
C(38)-C(36)-C(39)	108.01(17)
C(39)-C(36)-C(21)	112.22(15)
C(41)-C(40)-C(26)	111.88(13)
C(41)-C(40)-C(42)	107.92(15)
C(41)-C(40)-C(43)	108.18(14)
C(42)-C(40)-C(26)	109.64(13)
C(43)-C(40)-C(26)	110.17(13)
C(43)-C(40)-C(42)	108.97(14)

C(29)-C(44)-C(45)	107.57(15)
C(46)-C(44)-C(29)	111.93(14)
C(46)-C(44)-C(45)	107.97(16)
C(47)-C(44)-C(29)	111.61(15)
C(47)-C(44)-C(45)	110.65(19)
C(47)-C(44)-C(46)	107.07(17)
C(49)-C(48)-C(34)	110.22(14)
C(49)-C(48)-C(50)	109.41(17)
C(50)-C(48)-C(34)	108.94(14)
C(51)-C(48)-C(34)	112.58(15)
C(51)-C(48)-C(49)	107.94(16)
C(51)-C(48)-C(50)	107.68(16)
Cl(4)-C(52)-Cl(5)	112.75(16)

Symmetry transformations used to generate equivalent atoms: #1 -x+1, -y+1, -z+1

Table S7.17 Anisotropic displacement parameters ($Å^2 \times 10^3$) for **BN3**; the anisotropic displacement factor exponent takes the form: $-2\pi^2 [h^2 a^{*2}U^{11} + ... + 2hka^* b^* U^{12}]$

	U11	U22	U33	U23	U13	U12
C(1)	17(1)	21(1)	22(1)	-8(1)	-2(1)	-3(1)
B(1)	16(1)	19(1)	16(1)	-4(1)	-3(1)	2(1)
N(2)	17(1)	17(1)	20(1)	-5(1)	-4(1)	0(1)
C(2)	18(1)	23(1)	20(1)	-8(1)	-3(1)	1(1)
N(3)	16(1)	20(1)	16(1)	-5(1)	-2(1)	1(1)
C(3)	19(1)	20(1)	16(1)	-6(1)	-1(1)	-2(1)
N(4)	16(1)	19(1)	18(1)	-3(1)	-3(1)	1(1)
C(4)	16(1)	17(1)	17(1)	-5(1)	-4(1)	0(1)
C(5)	17(1)	16(1)	17(1)	-5(1)	-4(1)	-2(1)
C(6)	17(1)	17(1)	18(1)	-4(1)	-3(1)	-1(1)
C(7)	18(1)	18(1)	18(1)	-6(1)	-2(1)	-2(1)
C(8)	22(1)	16(1)	20(1)	-4(1)	-5(1)	-1(1)
C(9)	20(1)	16(1)	23(1)	-4(1)	-6(1)	-1(1)
C(10)	18(1)	18(1)	24(1)	-7(1)	-6(1)	-1(1)
C(11)	14(1)	22(1)	21(1)	-8(1)	0(1)	0(1)
C(12)	35(1)	24(1)	34(1)	-7(1)	-13(1)	-1(1)
C(13)	44(1)	21(1)	52(1)	-7(1)	-15(1)	-2(1)

C(14)	32(1)	29(1)	53(1)	-21(1)	-8(1)	-6(1)
C(15)	49(1)	36(1)	43(1)	-14(1)	-23(1)	-5(1)
C(16)	45(1)	25(1)	37(1)	-7(1)	-19(1)	-3(1)
C(17)	21(1)	20(1)	29(1)	-7(1)	-7(1)	1(1)
C(18)	18(1)	24(1)	33(1)	-13(1)	-4(1)	2(1)
C(19)	19(1)	23(1)	24(1)	-10(1)	0(1)	-2(1)
C(20)	19(1)	28(1)	22(1)	-8(1)	-1(1)	1(1)
C(21)	23(1)	30(1)	18(1)	-6(1)	1(1)	-1(1)
C(22)	22(1)	26(1)	18(1)	-6(1)	-3(1)	1(1)
C(23)	19(1)	22(1)	18(1)	-7(1)	-2(1)	0(1)
C(24)	17(1)	20(1)	16(1)	-6(1)	-1(1)	-1(1)
C(25)	21(1)	20(1)	15(1)	-4(1)	-4(1)	1(1)
C(26)	18(1)	17(1)	19(1)	-6(1)	-5(1)	0(1)
C(27)	16(1)	19(1)	18(1)	-5(1)	0(1)	-1(1)
C(28)	20(1)	22(1)	19(1)	-4(1)	-3(1)	1(1)
C(29)	22(1)	24(1)	17(1)	-5(1)	-1(1)	0(1)
C(30)	25(1)	24(1)	15(1)	-2(1)	-3(1)	1(1)
C(31)	21(1)	18(1)	18(1)	-4(1)	-5(1)	-2(1)
C(32)	22(1)	18(1)	21(1)	-4(1)	-4(1)	0(1)
C(33)	26(1)	21(1)	21(1)	-3(1)	-5(1)	-1(1)
C(34)	27(1)	19(1)	24(1)	-4(1)	-11(1)	2(1)
C(35)	22(1)	19(1)	28(1)	-7(1)	-7(1)	2(1)
C(36)	25(1)	46(1)	19(1)	-3(1)	2(1)	5(1)
C(37)	63(2)	68(2)	26(1)	-17(1)	4(1)	18(1)
C(38)	34(1)	78(2)	34(1)	7(1)	3(1)	-15(1)
C(39)	37(1)	60(1)	23(1)	4(1)	4(1)	6(1)
C(40)	19(1)	24(1)	19(1)	-6(1)	-5(1)	2(1)
C(41)	27(1)	41(1)	24(1)	-6(1)	-9(1)	11(1)
C(42)	24(1)	34(1)	48(1)	-8(1)	-12(1)	-4(1)
C(43)	28(1)	35(1)	31(1)	-13(1)	-7(1)	9(1)
C(44)	27(1)	33(1)	18(1)	-1(1)	2(1)	7(1)
C(45)	70(2)	40(1)	59(2)	-22(1)	31(1)	-3(1)
C(46)	28(1)	39(1)	25(1)	-1(1)	4(1)	-1(1)
C(47)	29(1)	122(2)	26(1)	1(1)	1(1)	27(1)
C(48)	29(1)	29(1)	26(1)	-5(1)	-12(1)	4(1)
C(49)	50(1)	37(1)	31(1)	4(1)	-18(1)	-1(1)
C(50)	45(1)	50(1)	39(1)	-19(1)	-22(1)	9(1)
C(51)	34(1)	54(1)	36(1)	-8(1)	-18(1)	12(1)
Cl(4)	203(1)	52(1)	76(1)	-26(1)	-7(1)	-17(1)
Cl(5)	149(1)	80(1)	59(1)	-20(1)	-4(1)	-7(1)
C(52)	76(2)	73(2)	75(2)	-42(2)	4(2)	-22(2)

Table S7.18 Hydrogen coordinates (x 10^4) and isotropic displacement parameters (Å^2 x 10^3) for **BN3**.

	x	y	z	U(eq)
H(12)	-729	6154	946	36
H(13)	-1362	4683	630	47
H(14)	-2278	4902	-666	42
H(15)	-2608	6619	-1618	48
H(16)	-1995	8090	-1302	42
H(17)	-3826	10896	-577	27
H(18)	-4747	10526	947	29
H(19)	-3867	9260	2094	26
H(20)	-3160	7870	3062	28
H(22)	-58	6205	3724	27
H(25)	2346	6060	2712	23
H(27)	3865	7072	54	22
H(28)	3670	7412	-1243	25
H(30)	2384	9620	-3365	27
H(33)	67	10689	-3651	28
H(35)	-3094	10916	-1993	27
H(37A)	-1890	7869	4891	81
H(37B)	-3042	7280	5551	81
H(37C)	-3179	8034	4539	81
H(38A)	-4047	6529	4190	81
H(38B)	-3912	5769	5201	81
H(38C)	-3310	5409	4320	81
H(39A)	-1342	5140	4931	68
H(39B)	-1993	5526	5789	68
H(39C)	-807	6098	5170	68
H(41A)	4235	5988	3035	47
H(41B)	5357	5232	2798	47
H(41C)	4003	4887	2858	47
H(42A)	5478	7365	734	52
H(42B)	6244	6702	1524	52
H(42C)	5144	7486	1748	52
H(43A)	4490	4698	1265	46
H(43B)	5845	4983	1280	46
H(43C)	5137	5648	453	46

H(45A)	3902	6683	-2782	89
H(45B)	4803	7116	-3722	89
H(45C)	3397	7466	-3670	89
H(46A)	3943	9307	-4264	51
H(46B)	5364	9047	-4263	51
H(46C)	4681	9847	-3726	51
H(47A)	5566	8647	-2329	99
H(47C)	6172	7961	-2997	99
H(47B)	5364	7391	-2042	99
H(49A)	-1745	12954	-3968	61
H(49B)	-1970	12788	-4913	61
H(49C)	-742	12335	-4507	61
H(50A)	-1191	10522	-4606	63
H(50B)	-2429	11005	-4983	63
H(50C)	-2446	9989	-4091	63
H(51A)	-3972	10978	-3240	63
H(51B)	-3912	11959	-4161	63
H(51C)	-3696	12145	-3221	63
H(52A)	1278	7253	-2559	85
H(52B)	80	6688	-1953	85

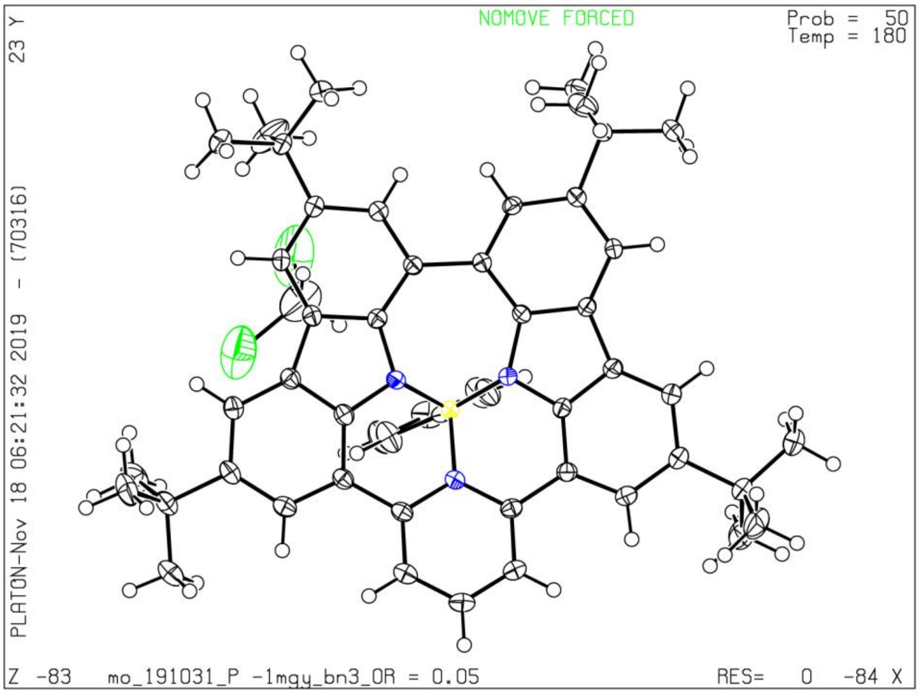

The ORTEP diagram showing the structure of **BN3** with labeling schemes

S8. MR-TADF k_{RISC} literature study

Table **S8.1.** Photophysical data of previous MR-TADF emitters with and without carbazole substitution.

Compound	ΔE_{ST} / eV	k_{RISC} / × 10^2 s^{-1}	No. Carbazoles	Ref.
**BBCz-SB**	0.13[a]	140[a]	0	28
***m*-CzBNCz**	0.08[a]	10800[b]	1	29
**BBCz-Y**	0.14[a]	1000[a]	2	28
**BBCz-G**	0.14[a]	1800[a]	3	28
**BN1**	0.11[a]	1900[c]	2	30
**CNCz-BNCz**	0.18[a]	4200[a]	2	31
**DiKTa**	0.19[a]	23[a]	0	32
**QAD-Cz**	0.17[d]	440[d]	1	33
**QAD-2Cz**	0.17[e]	840[e]	2	33
**BN1**	0.20[f]	2900[g]	0	This work
**TCz-BN1**	0.16[f]	4670[g]	1	This work
**BN2**	0.19[f]	2100[h]	0	This work
**TCz-BN2**	0.17[f]	2440[h]	1	This work

[a] Dilute toluene solution, [b] 10 wt% PhCzBCz, c 1 wt% mCBP, d 1 wt% mCP, e 12 wt% mCP, f 5 wt% PMMA, g 2 wt% mCBP, h 5 wt% mCBP.

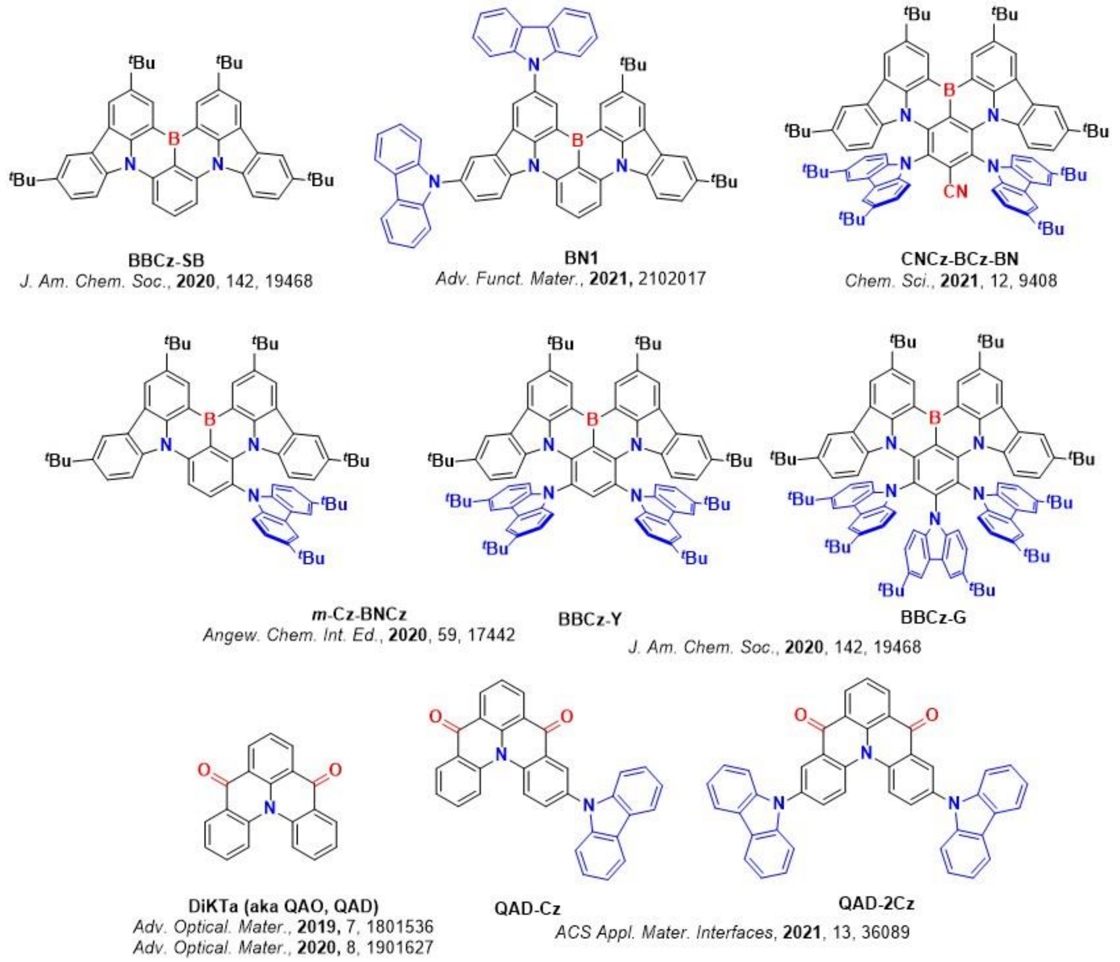

Figure **S8.1**. Structures of previously reported MR-TADF emitters with and without carbazole substitution.

S9 References

[1] M. J. Frisch, G. W. Trucks, H. B. Schlegel, G. E. Scuseria, M. A. Robb, J. R. Cheeseman, G. Scalmani, V. Barone, G. A. Petersson, H. Nakatsuji, X. Li, M. Caricato, A. V. Marenich, J. Bloino, B. G. Janesko, R. Gomperts, B. Mennucci, H. P. Hratchian, J. V. Ortiz, A. F. Izmaylov, J. L. Sonnenberg, Williams, F. Ding, F. Lipparini, F. Egidi, J. Goings, B. Peng, A. Petrone, T. Henderson, D. Ranasinghe, V. G. Zakrzewski, J. Gao, N. Rega, G. Zheng, W. Liang, M. Hada, M. Ehara, K. Toyota, R. Fukuda, J. Hasegawa, M. Ishida, T. Nakajima, Y. Honda, O. Kitao, H. Nakai, T. Vreven, K. Throssell, J. A. Montgomery Jr., J. E. Peralta, F. Ogliaro, M. J. Bearpark, J. J. Heyd, E. N. Brothers, K. N. Kudin, V. N. Staroverov, T. A. Keith, R. Kobayashi, J. Normand, K. Raghavachari, A. P. Rendell, J. C. Burant, S. S. Iyengar, J. Tomasi, M. Cossi, J. M. Millam, M. Klene, C. Adamo, R. Cammi, J. W. Ochterski, R. L. Martin, K. Morokuma, O. Farkas, J. B. Foresman, D. J. Fox, Wallingford, CT, 2016.

[2] TURBOMOLE V7.4 2017, a development of University of Karlsruhe and Forschungszentrum Karlsruhe GmbH, 1989-2007, TURBOMOLE GmbH, since

2007; available from http://www.turbomole.com.
[3] A. D. Becke, *J. Chem. Phys.* 1993, **98**, 5648.
[4] C. Adamo; V. Barone, *J. Chem. Phys.* 1999, **110**, 6158.
[5] O. A. Vydrov; G. E. Scuseria, *J. Chem. Phys.* 2006, **125**, 234109
[6] T. Yanai; D. P. Tew; N. C. Handy, *Chem. Phys. Lett.* 2004, **393**, 51
[7] Y. Zhao; D. G. Truhlar, *Theor. Chem. Acc.* 2008, **120**, 215.
[8] T. H. Dunning, *J. Chem. Phys* 1989, **90**, 1007.
[9] S. Hirata and M. Head-Gordon, *Chem. Phys. Lett.* 1999, **314** (3-4), 291
[10] T. Etienne, X. Assfeld, A. Monari, *J. Chem. Theory Comput.* 2014, **10**, 3896-3905
[11] R. K. Dennington, T.; Millam, J, KS, Semichem Inc.: Shawnee Mission, 2019
[12] A. Hellweg, S. A. Grün and C. Hättig, *Phys. Chem. Chem. Phys*., 2008, **10,** 4119-4127
[13] G. A. Petersson and M. A. Al-Laham, *J. Chem. Phys.* 1991, **94** (9), 6081
[14] C. Hättig and F. Weigend; *J. Chem. Phys.*, 2000, **113**, 5154-5161.
[15] C. Hättig and K. Hald, *Phys. Chem. Chem. Phys.*2002, **4**, 2111-2118.
[16] K. Momma and F. Izumi, *J. Appl. Crystallogr.*, 2011, **44**, 1272-1276.
[17] C. Maeda, T. Todaka, T. Ueda, T. Ema, *Chem. Eur. J.* 2016, **22**, 7508-7513.
[18] S. M. Kim, S. Y. Byeon, S.-H. Hwang and J. Y. Lee. *Chem. Commun.,* 2015, **51**, 10672-10675.
[19] C. Yin, D. Zhang, Y. Zhang, Y. Lu, R. Wang, G. Li, and L. Duan. *CCS Chem.* 2020, **2**, 1268-1277.
[20] H. Kaji, H. Suzuki, T. Fukushima, K. Shizu, K. Suzuki, S. Kubo, T. Komino, H. Oiwa, F. Suzuki, A. Wakamiya, Y. Murata and C. Adachi, *Nat Commun* 2015, **6**, 8476.
[21] Y. J. Shiu, Y. C. Cheng, W. L. Tsai, C. C. Wu, C. T. Chao, C. W. Lu, Y. Chi, Y. T. Chen, S. H. Liu and P. T. Chou, *Angew. Chem. Int. Ed.*, 2016, **55**, 3017-3021.
[22] K. Matsuo and T. Yasuda, *Chem. Commun.*, 2017, **53**, 8723-8726.
[23] Y.-J. Shiu, Y.-T. Chen, W.-K. Lee, C.-C. Wu, T.-C. Lin, S.-H. Liu, P.-T. Chou, C.-W. Lu, I. C. Cheng, Y.-J. Lien and Y. Chi, *Journal of Materials Chemistry C*, 2017, **5**, 1452-1462.
[24] B. M. Bell, T. P. Clark, T. S. De Vries, Y. Lai, D. S. Laitar, T. J. Gallagher, J.-H. Jeon, K. L. Kearns, T. McIntire, S. Mukhopadhyay, H.-Y. Na, T. D. Paine and A. A. Rachford, *Dyes Pigm.*, 2017, **141**, 83-92.
[25] M. Mamada, G. Tian, H. Nakanotani, J. Su and C. Adachi, *Angew. Chem. Int. Ed.*, 2018, **57**, 12380-12384.
[26] P. Li, H. Chan, S. L. Lai, M. Ng, M. Y. Chan and V. W. Yam, *Angew. Chem. Int. Ed.*, 2019, **58**, 9088-9094.
[27] D.-H. Kim, A. D'Aléo, X.-K. Chen, A. D. S. Sandanayaka, D. Yao, L. Zhao, T. Komino, E. Zaborova, G. Canard, Y. Tsuchiya, E. Choi, J. W. Wu, F. Fages, J.-L. Brédas, J.-C. Ribierre and C. Adachi, *Nat. Photonics*, 2018, 12, 98-104.
[28] M. Yang, I. S. Park and T. Yasuda, *J. Am. Chem. Soc.*, 2020, **142**, 19468-19472.
[29] Y. Xu, C. Li, Z. Li, Q. Wang, X. Cai, J. Wei and Y. Wang, *Angew. Chem. Int. Ed.*, 2020, **59**, 17442-17446.

[30] Y. Qi, W. Ning, Y. Zou, X. Cao, S. Gong and C. Yang, *Adv. Funct. Mater.*, 2021, **31**, 2102017.

[31] Y. Liu, X. Xiao, Y. Ran, Z. Bin and J. You, *Chem. Sci.*, 2021, **12**, 9408-9412.

[32] D. Hall, S. M. Suresh, P. L. dos Santos, E. Duda, S. Bagnich, A. Pershin, P. Rajamalli, D. B. Cordes, A. M. Z. Slawin, D. Beljonne, A. Köhler, I. D. W. Samuel, Y. Olivier and E. Zysman-Colman, *Adv. Opt. Mater.*, 2020, **8**, 1901627.

[33] F. Huang, K. Wang, Y.-Z. Shi, X.-C. Fan, X. Zhang, J. Yu, C.-S. Lee and X.-H. Zhang, *ACS Appl. Mater. Interfaces*, 2021, **13**, 36089-36097.
